# Improved deep reinforcement learning controls anti-lock braking via electro-hydraulic composite systems in in-wheel motor-driven electric vehicles

**DOI:** 10.1038/s41598-026-59739-6

**Published:** 2026-07-06

**Authors:** Yalei Liu, Xiang Zhang, Xinyu Qi, Tianshi Jin, Mingliang Yang, Weiping Ding

**Affiliations:** 1 School of Mechanical Engineering, Southwest Jiaotong University, Chengdu, 610031 China; 2 Chongqing Seres Phoenix Intelligent Innovation Technology Co., Ltd., Chongqing, 400041 China

**Keywords:** In-wheel motor-driven electric vehicle, Electro-hydraulic composite braking, Anti-lock braking system, Deep reinforcement learning, Soft actor-critic, Engineering, Mathematics and computing

## Abstract

Anti-lock braking control for in-wheel motor-driven electric vehicles is challenging because electro-hydraulic composite braking combines fast and accurately controllable motor regenerative braking with slower hydraulic pressure regulation. Existing ABS strategies often disable regenerative braking during ABS intervention, which limits both braking performance and energy recovery. This paper proposes a delay-aware action-space-grouped Soft Actor-Critic controller, termed SAC-DA-ASG, for coordinated ABS control of the motor regenerative braking system and the hydraulic braking system. The proposed controller addresses two implementation issues. First, an action-space grouping scheme maps coordinated solenoid-valve operations into a single hydraulic mode, reducing invalid actuator combinations and the policy search space. Second, a delay-aware augmented state incorporates recent system states to compensate for heterogeneous actuator response delays and improve the Markov representation. The controller is trained under randomized road-adhesion and actuator conditions and is evaluated against baseline SAC variants and a Bosch-type ABS strategy. Simulation results show that SAC-DA-ASG keeps the wheel slip ratio closer to the target value of 0.15, reduces slip fluctuation and peak slip, and shortens braking distance under uniform and split- *μ* road conditions. Sensitivity tests under solenoid-valve delay and vehicle-mass perturbations further indicate robust closed-loop behavior. Real-time hardware-in-the-loop tests on a Speedgoat platform show close agreement with simulation in braking time, stopping distance, and slip-ratio regulation. These results demonstrate the feasibility of DRL-based coordinated electro-hydraulic ABS control for in-wheel motor-driven electric vehicles.

## Introduction

The braking system of in-wheel motor-driven electric vehicles adopts an electro-hydraulic composite braking architecture, which integrates a motor regenerative braking system (MRBS) and a hydraulic braking system (HBS). Braking torque can be independently controlled via each in-wheel motor, enabling millisecond-level response times that are well-suited to the rapid adjustments required during braking. HBS acts as a complementary source of braking force, providing critical support for enhancing overall braking reliability^1,2^.

Numerous studies have employed rule-based or fuzzy logic control, optimization strategies, model predictive control, and sliding mode control to enable in-wheel motor-driven electric vehicles to achieve reliable braking performance under non-emergency conditions while maximizing braking energy recovery^3–11^. However, these approaches are limited to non-emergency braking scenarios. During emergency braking, the ABS is activated to prevent wheel lock-up and suppress braking instability. By regulating wheel slip ratios, ABS enhances braking effectiveness and ensures braking safety. For in-wheel motor-driven electric vehicles, however, a key challenge remains: how to fully exploit the advantages of electro-hydraulic composite braking—particularly the rapid responsiveness and energy recovery capabilities of the MRBS—to achieve efficient ABS control.

In current research and practical applications of ABS electro-hydraulic composite braking systems, the braking torque generated by in-wheel motors is often affected by factors such as motor speed and temperature. As a result, most existing control strategies deactivate the MRBS when the ABS is engaged, relying solely on the opening and closing of solenoid valves in the HBS to modulate brake pressure^12–14^. While this approach simplifies control logic, it neglects the inherent advantages of MRBS, including rapid response, high controllability. Consequently, the dynamic performance of ABS is limited. Moreover, frequent transitions of wheel states across slip control thresholds can further degrade overall braking effectiveness. In studies focusing on the coordinated control of MRBS and HBS to enable electro-hydraulic composite braking for ABS. Yang, et al^15^. to automatically adjust the regenerative braking intensity. This approach was shown to support smooth and efficient braking performance. However, their work targeted centrally driven electric vehicles rather than in-wheel motor architectures. Basrah, et al^16^. developed a hybrid braking torque allocation algorithm that distributes torque between MRBS and HBS based on predefined energy recovery priorities. Lv, et al^17^. proposed a sliding mode control algorithm aimed at enhancing ABS performance in in-wheel motor-driven electric vehicles during emergency braking scenarios. Jin, et al^18^. introduced a braking torque distribution method based on sliding mode control, enabling coordinated allocation of four-wheel hydraulic braking and regenerative torque under critical conditions. While these approaches have contributed to improving the ABS performance of electro-hydraulic composite braking systems to some extent, none have explicitly addressed the distinct response delays between MRBS and HBS in in-wheel motor vehicle architectures.

Compared with the motor torque response of MRBS, the hydraulic response of HBS exhibits significant differences, characterized by larger time delays, overshoot, and reduced accuracy. Therefore, achieving high-quality coordination in Electro-Hydraulic Composite Braking is crucial for ensuring a consistent braking response^19^. To mitigate torque fluctuations arising from the inconsistent responses of the two braking subsystems, Numasato et al. intentionally delayed the motor braking response to align their dynamics and thereby improve consistency^20^, but this strategy inevitably introduces overall response lag. Ko et al. designed distinct filtering algorithms for the motor and hydraulic subsystems in an attempt to attenuate the inconsistency without sacrificing system responsiveness^4^, however, their effectiveness in transient regimes remains insufficient, especially during ABS control. Todeschini et al. employed a compensator to accelerate the hydraulic braking response^21^, yet it is difficult to effectively eliminate the delay in the pressurization phase. R Zhang proposed an optimization strategy for the torque distribution coefficient and a personalized MPC control strategy to address the inconsistency in braking feel caused by response delays under different driving behaviors^22^. Overall, these methods largely rely on static or preset compensation and filtering mechanisms and, from a control-method perspective, still struggle to fundamentally cope with the uncertainties introduced by time-varying delays, strong nonlinear couplings, and complex operating conditions. In light of the foregoing—namely, the pronounced dynamic-response discrepancy between MRBS and HBS in Electro-Hydraulic Composite Braking and the limitations of conventional compensation/filtering strategies under time-varying delay and strongly nonlinear conditions—it is particularly necessary to introduce machine learning methods with online learning and adaptive capabilities.

**Fig. 1:**
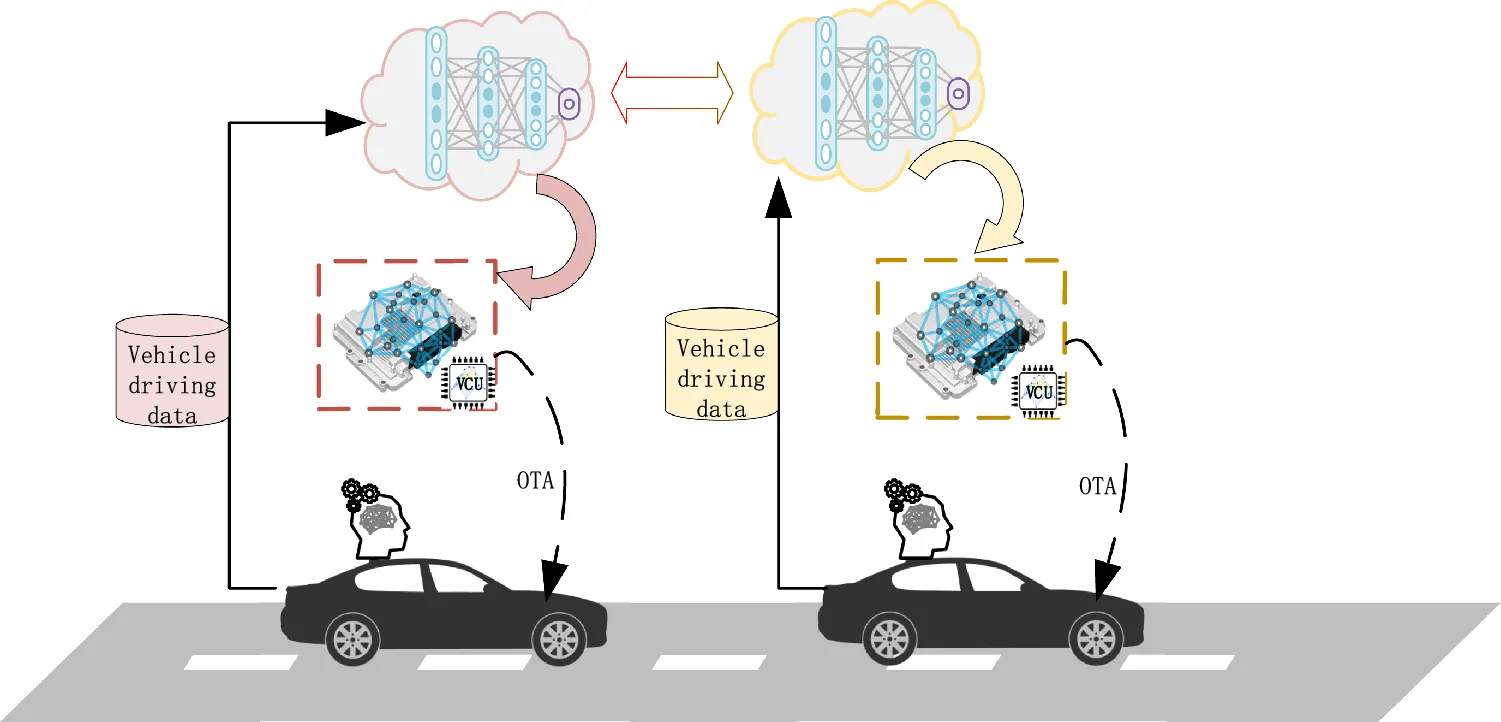
Intelligent and connected transportation ecosystem.

Deep reinforcement learning (DRL), as a major branch of machine learning, has demonstrated significant potential for controlling nonlinear systems and has seen growing adoption in the nonlinear systems control domain in recent years^23–27^. By enabling agents to learn near-optimal control policies through interaction with complex and uncertain environments, DRL reduces the dependence on high-fidelity vehicle models and computationally intensive solvers. This capability offers efficient decision-making and control execution, positioning DRL as a promising approach for the development and deployment of control strategies in intelligent and connected vehicles. By leveraging the flexible control capabilities provided by in-wheel drive systems and the intelligent decision-making and execution offered by DRL, it becomes possible to establish a truly intelligent and connected transportation ecosystem. When combined with cloud-based training and evolutionary learning paradigms, this framework enables continuous adaptation and optimization across vehicles and environments (as illustrated in Fig. 1). Among the many algorithmic implementations of DRL, the Soft Actor-Critic (SAC) algorithm has demonstrated strong capabilities in exploring complex policy spaces for multi-objective optimization tasks and has been widely adopted in control research^28–30^. In the field of conventional fuel vehicle control, researchers have explored the application of DRL to ABS scenarios involving only hydraulic braking and have achieved moderate success^31^. However, to the best of the authors’ knowledge, applying DRL to ABS control in in-wheel motor-driven electric vehicles presents two key challenges, no existing study has comprehensively addressed these two challenges. First, the braking system involves a large number of actuators; this multi-actuator configuration leads to a high-dimensional action space, which significantly increases the complexity of policy learning. Second, MRBS and HBS exhibit distinct response characteristics^32^, making it difficult for DRL algorithms to achieve stable convergence. Overcoming these two issues is essential for enabling the practical application of DRL in ABS control for in-wheel motor-driven electric vehicles. In addition, the demand for faster training convergence further constrains the scalability and real-world deployment of DRL-based control methods.

In summary, to address the aforementioned challenges, this study proposes an improved DRL-based ABS control strategy for in-wheel motor-driven electric vehicles with electro-hydraulic composite braking systems, featuring the following key innovations: (1) The main contribution of this paper is the development of a novel SAC-DA-ASG control strategy. By synergistically integrating Delay-Aware (DA) state space mapping to restore Markov properties and discrete Action Space Graining (ASG) for dimensionality reduction within the Soft Actor-Critic (SAC) framework, this strategy effectively solves the practical control challenges of electro-hydraulic composite braking in electric vehicles, specifically addressing the issues of high-dimensional actuator spaces and heterogeneous response delays;(2) Parallelization architecture for DRL training: A parallel training framework is constructed based on asynchronous experience replay and distributed environment interaction. This architecture significantly accelerates training.

The remainder of this paper is organized as follows: Sect. 2 introduces the proposed control strategy along with relevant parameter design. Section 3 presents the training process and analyzes the experimental results. Finally, Sect. 4 concludes the study and summarizes the key findings.

## Algorithmic framework for DRL

**Fig. 2:**
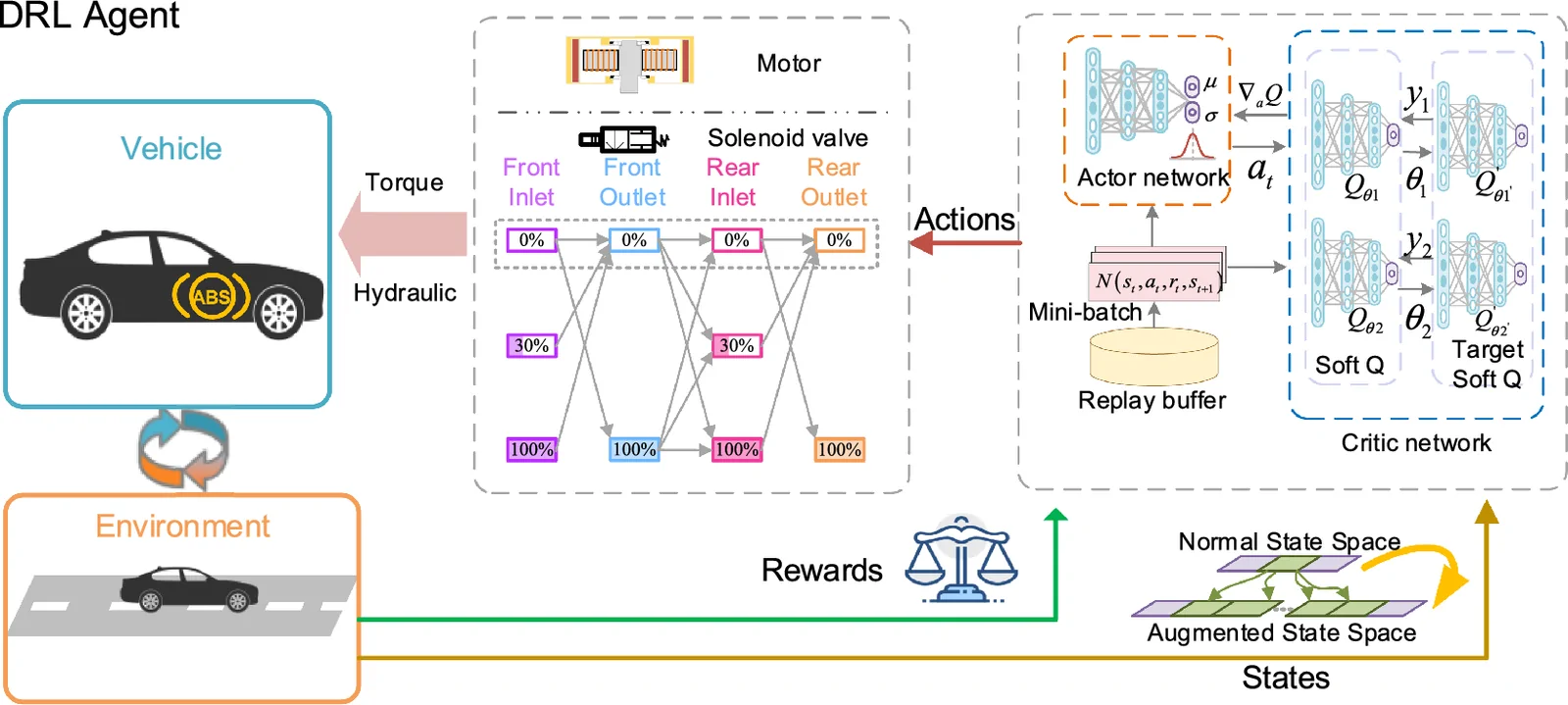
The control architecture proposed in this paper.

DRL learns optimal policies through interaction with the environment, making it particularly well-suited for controlling highly nonlinear and time-varying systems. To address the nonlinear characteristics and multi-actuator coordination challenges of ABS control in the electro-hydraulic composite braking system of in-wheel motor-driven electric vehicles, a control architecture is proposed, as illustrated in Fig. 2. The term “deep”refers to the use of artificial neural networks with more than two layers, serving as function approximators to map system states to actions and expected discounted rewards. A typical DRL framework requires an agent that interacts with the environment to generate actions and a policy that assigns rewards to those actions. In this study, the model serves as the environment for DRL, and the SAC algorithm is employed and further enhanced. The resulting method—Soft Actor-Critic with delay awareness and action space grouping (SAC-DA-ASG)—enables integrated ABS control by coordinating MRBS and HBS within the electro-hydraulic composite braking system.

The following section introduces the underlying model and presents the design of the corresponding control strategy.

### Electro-hydraulic composite braking system structure and modeling

#### Electro-hydraulic composite braking system structure

**Fig. 3:**
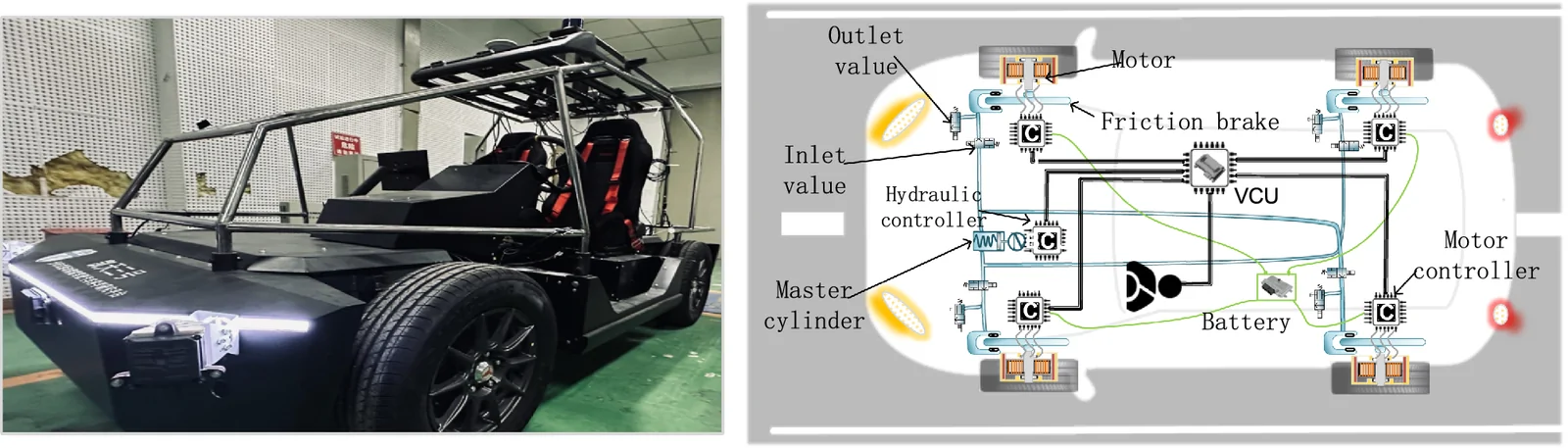
In-wheel motor vehicle experimental platform and structure of the electro-hydraulic.

This study focuses on an in-wheel motor vehicle equipped with a steer-by-wire chassis. The reference model, including its parameters, is derived from the vehicle experimental platform shown in Fig. 3. The electro-hydraulic composite braking system consists of both MRBS and HBS. When the driver presses the brake pedal, the vehicle controller calculates the braking intensity and the required total braking force based on the pedal displacement. The motor controller applies regenerative braking torque to the corresponding wheels via the in-wheel motors. Simultaneously, the hydraulic controller actuates the HBS unit to apply hydraulic braking torque to the appropriate wheels. The hydraulic circuit is equipped with solenoid valves, which modulate wheel cylinder pressure through coordinated opening and closing actions.

#### Vehicle model

**Fig. 4:**
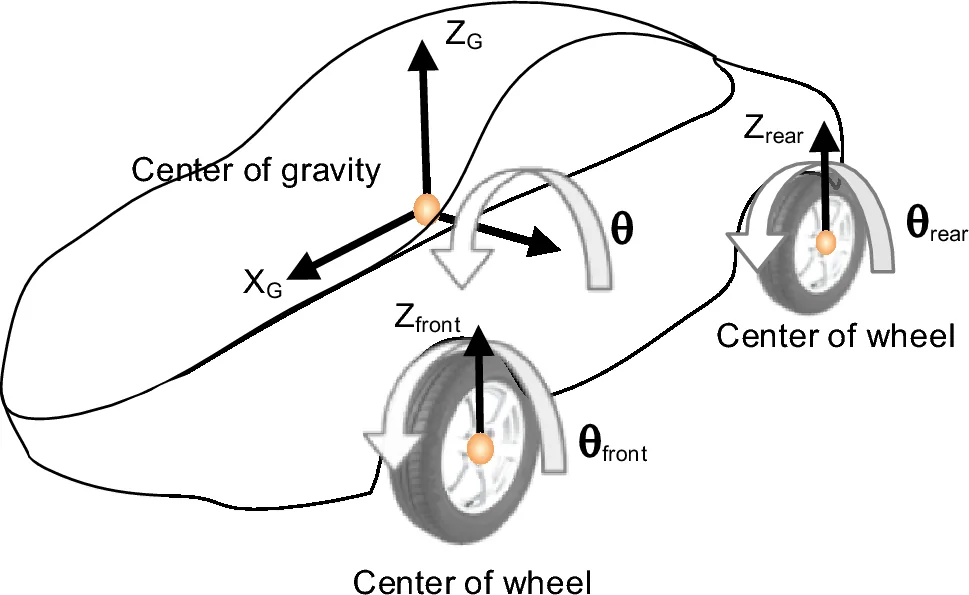
Seven-degree-of-freedom vehicle dynamic model.

Since this study primarily focuses on braking performance and wheel slip behavior—i.e., the longitudinal dynamics of the vehicle—a simplified yet representative dynamic model is constructed. Specifically, a seven-degree-of-freedom (7-DOF) vehicle model is developed, consisting of three degrees of freedom for the vehicle body and one degree of freedom for each of the four wheels, as shown in Fig. 4. The corresponding dynamic equations are formulated as follows^33,34^:

1 $$\left\{ \begin{gathered} ma_{v} = - F_{xb} - F_{aero} - F_{roll} \hfill \\ m_{spr} \ddot{z} = 2(F_{fsz} + F_{rsz} ) - m_{spr} g \hfill \\ I_{y} \ddot{\theta } = 2(L_{f} F_{fsz} - L_{r} F_{rsz} ) \hfill \\ m_{fw} \ddot{z}_{fw} = k_{fs} (z - z_{fw} ) + c_{fs} (\dot{z} - \dot{z}_{fw} ) - F_{fwz} \hfill \\ m_{rw} \ddot{z}_{rw} = k_{rs} (z - z_{rw} ) + c_{rs} (\dot{z} - \dot{z}_{rw} ) - F_{rwz} \hfill \\ I_{w} \ddot{\omega}_{fw} = F_{xbf} r - T_{bf} - T_{rollf} \hfill \\ I_{w} \ddot{\omega}_{rw} = F_{xbr} r - T_{br} - T_{rollr} \hfill \\ \end{gathered} \right.$$

In the equation,*m* represents the vehicle’s curb weight;$m_{spr}$ is the sprung mass;$m_{fw}$ and $m_{rw}$ are the front and rear wheel masses, respectively. $a_{v}$ denotes the vehicle’s longitudinal acceleration. *z*,$\dot{z}$ and $\ddot{z}$ represent the vehicle’s vertical displacement, velocity, and acceleration, respectively. $\ddot{\theta }$ is the vehicle’s pitch angular acceleration;$z_{fw}$,$\dot{z}_{fw}$ and $\ddot{z}_{fw}$ represent the vertical displacement, velocity, and acceleration of the front wheels, respectively. $z_{rw}$,$\dot{z}_{rw}$ and $\ddot{z}_{rw}$ represent the vertical displacement, velocity, and acceleration of the rear wheels, respectively. $\dot{\omega}_{fw}$ and $\dot{\omega}_{rw}$ are the angular accelerations of the front and rear wheels, respectively. $F_{xb}$ denotes the ground friction force. $F_{aero}$ is the air resistance. $F_{roll}$ is the rolling resistance. $F_{fsz}$ and $F_{rsz}$ represent the vertical forces provided by the front and rear suspension, respectively. $I_{y}$ is the moment of inertia of the sprung mass about the y-axis;$L_{f}$ and $L_{r}$ represent the front and rear wheelbase, respectively. $k_{fs}$ and $k_{rs}$ are the stiffness coefficients of the front and rear suspensions, respectively. $c_{fs}$ and $c_{rs}$ are the damping coefficients of the front and rear suspensions, respectively. $F_{fwz}$ and $F_{rwz}$ represent the vertical forces between the front and rear wheels and the ground, respectively;$I_{w}$ is the moment of inertia of the wheels. $F_{xbf}$ and $F_{xbr}$ are the longitudinal braking forces between the front and rear wheels and the ground, respectively; *r* is the rolling radius of the wheels. $T_{bf}$ and $T_{br}$ are the total braking torques acting on the front and rear wheels, respectively. $T_{rollf}$ and $T_{rollr}$ are the rolling resistance torques acting on the front and rear wheels, respectively. The parameters used in the vehicle model are based on the actual specifications of the in-wheel motor experimental platform shown in Fig. 3. A summary of the vehicle parameters is provided in Table 1.

**Table 1:** Vehicle model parameters.

Vehicle parameters	Value	Unit
*m*	800	kg
*m*_*spr*_	720	
*I*_*y*_	1500	kg∙m^2^
*L*_*f*_	1040	mm
*L*_*r*_	960	
*h*_*g*_	500	mm
*k*_*fs*_	20000	N/m
*k*_*rs*_	25000	
*c*_*fs*_	2000	N/(m/s)
*c*_*rs*_	2000	
*I*_*w*_	0.5	kg∙m^2^
*r*	320	mm
*R*_*b*_	1.25	mΩ
*Q*_*b*_	100	Ah
Rated voltage of battery	72	V
（front/rear）	1/0.625	mm^2^
（front/rear）	1/0.625	
*K*_*f*_	0.76	-
*K*_*r*_	0.76	
*D*_*f*_	48	
*R*_*f*_	96	
*D*_*r*_	38	
*R*_*r*_	94	

#### Tire model

The structural parameters and mechanical characteristics of the tires have a significant impact on the dynamic performance of the vehicle^35^. In this study, the Magic Formula model, which reflects the dynamic characteristics of the tire, is selected. Its formulation is as follows^36^:

2 $$\left\{ \begin{gathered} y(x) = D\sin \left[ {C\arctan (\varphi )} \right] \hfill \\ \varphi = Bx - E\left[ {Bx - \arctan (Bx)} \right] \hfill \\ Y(X) = y(X + S_{H} ) + S_{{_{V} }} \hfill \\ \lambda = \frac{{V_{xw} - V_{rw} }}{{V_{xw} }} \hfill \\ \alpha = arctan\frac{{V_{yw} }}{{V_{xw} }} \hfill \\ V_{rw} = \omega r_{w} \hfill \\ \end{gathered} \right.$$

The tire longitudinal force factor is:

3 $$\left\{ \begin{gathered} C_{x} = b_{0} \hfill \\ D_{x} = b_{1} F_{z}^{2} + b_{{2}} F_{z} \hfill \\ B_{x} = \frac{{(b_{3} F_{z}^{2} + b_{4} F_{z} )e^{{( - b_{5} F_{z} )}} }}{CD} \hfill \\ E_{x} = b_{{6}} F_{z}^{2} + b_{{7}} F + b_{{8}} \hfill \\ \end{gathered} \right.$$

The tire lateral force factor is:

4 $$\left\{ \begin{gathered} C_{y} = a_{0} \hfill \\ D_{y} = a_{1} F_{z}^{2} + a_{{2}} F_{z} \hfill \\ B_{y} = \frac{{a_{3} \sin \left[ {2\arctan (F_{z} /a_{4} )} \right](1 - a_{5} \left| \gamma \right|)}}{CD} \hfill \\ E_{y} = a_{{6}} F_{z}^{2} + a_{{7}} F + a_{8} \hfill \\ \end{gathered} \right.$$

In the formula,*X* represents the tire longitudinal slip ratio *λ* or the tire slip angle *α*. *Y* is the longitudinal force, lateral force, or restoring moment applied to the tire. $S_{H}$ and $S_{{_{V} }}$ are the horizontal and vertical offset constants, respectively. $V_{rw}$ is the product of wheel speed *ω* and the effective tire radius $r_{w}$. $V_{xw}$ and $V_{yw}$ are the longitudinal and lateral velocities at the wheel center, respectively; *B*is the stiffness factor. *C* is the shape factor; *D* is the peak factor. *E* is the curvature factor. $F_{z}$ is the tire vertical load. $a_{0}$ ~ $a_{8}$ and $b_{0}$ ~ $b_{8}$ are the fitting parameters. The tire fitting parameters are presented in Table 2.

**Table 2:** Fitting parameters of the tire magic formula model.

**Parameters**	**Value**	**Parameters**	**Value**
*a*_*0*_	1.6	*b*_*0*_	1.55
*a*_*1*_	−34	*b*_*1*_	0
*a*_*2*_	1250	*b*_*2*_	1000
*a*_*3*_	2320	*b*_*3*_	60
*a*_*4*_	12.8	*b*_*4*_	300
*a*_*5*_	0	*b*_*5*_	0.17
*a*_*6*_	−0.0053	*b*_*6*_	0
*a*_*7*_	0.1925	*b*_*7*_	0
*a*_*8*_	0	*b*_*8*_	0.2

#### Motor model

**Fig. 5:**
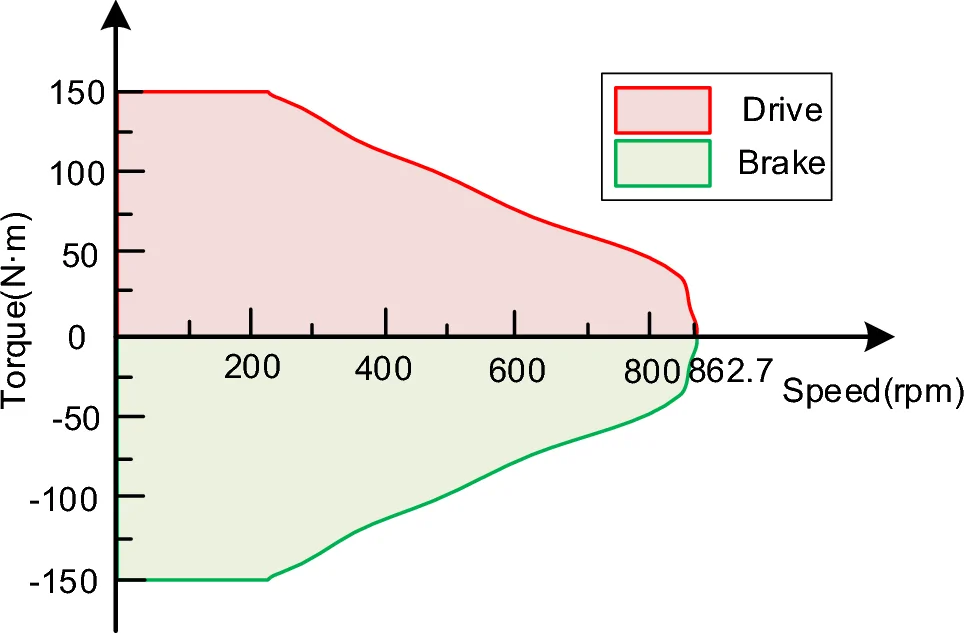
Motor’s external characteristics.

The front and rear motors of the vehicle studied in this paper are both of the same model, permanent magnet synchronous motors. The motor’s external characteristics are shown in Fig. 5. The motor power can be expressed as^37^:

5 $$P_{m} = \left\{ {\begin{array}{*{20}c} {\frac{{T_{m} n_{m} }}{{9550\eta_{m} }} \, motor} \\ {\frac{{T_{m} n_{m} \eta_{m} }}{9550} \, generator} \\ \end{array} } \right.$$

In the equation, $P_{m}$ is the motor power, $T_{m}$ is the torque, $n_{m}$ is the rotational speed, $\eta_{m}$ is the efficiency.

#### Battery model

**Fig. 6:**
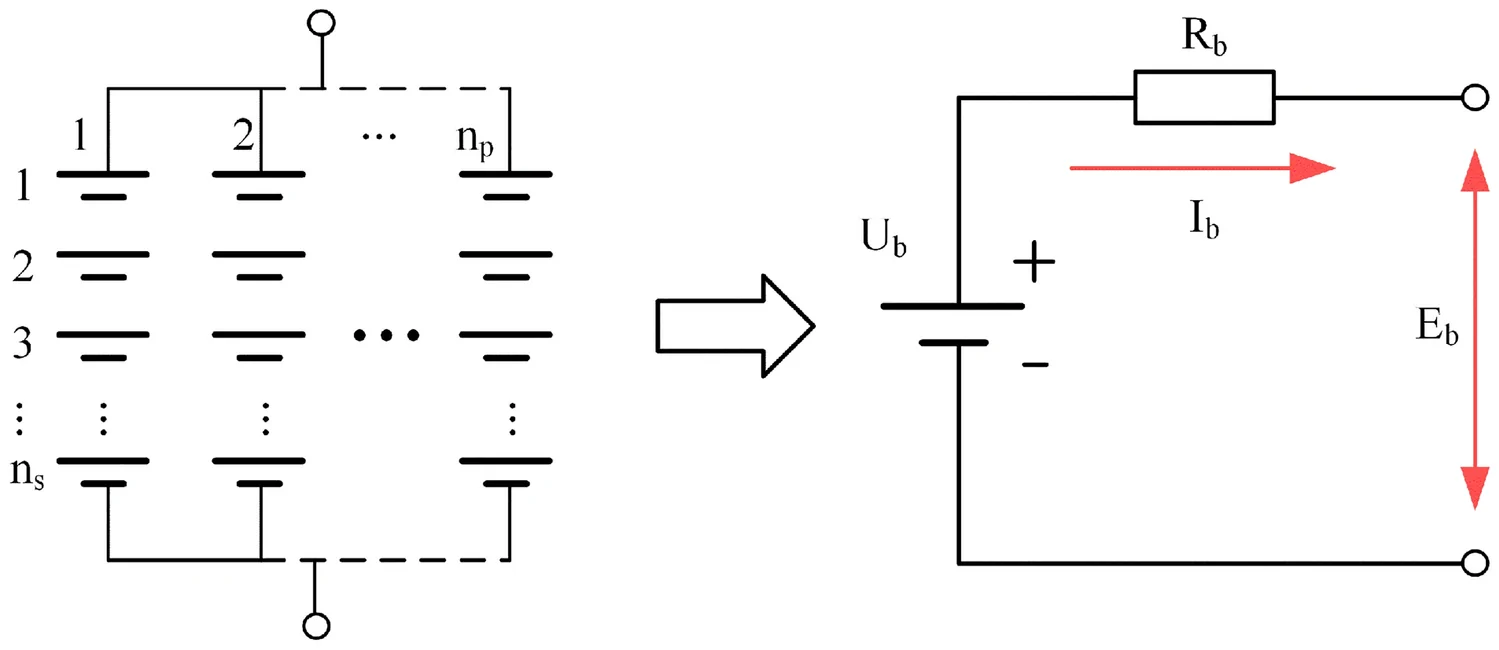
Vehicle battery system model.

As shown in Fig. 6, the battery pack used in the in-wheel motor vehicle studied in this work is composed of multiple individual cells. Since the detailed charge–discharge dynamics of the battery are not the focus of this study, a simplified and equivalent modeling approach is adopted. The battery pack is approximated as a single equivalent cell, modeled as a voltage source in series with an internal resistance. According to Kirchhoff’s law, the voltage balance equation and the state of charge (SOC) can be expressed as follows^38,39^:

6 $$\left\{ \begin{gathered} U_{b} = E_{b} - I_{b} R_{b} \hfill \\ SOC = SOC_{{\mathrm{int}}} - \frac{1}{{Q_{b} }}\int {I_{b} dt} \hfill \\ \end{gathered} \right.$$

In the equation, $E_{b}$ is the battery terminal voltage, $I_{b}$ is the battery pack line current, $R_{b}$ is the internal resistance of the battery pack, $U_{b}$ is the open-circuit voltage of the battery pack, $SOC_{{\mathrm{int}}}$ is the initial state of charge (SOC), and $Q_{b}$ is the battery capacity.

#### Hydraulic braking system model

The variation in wheel cylinder pressure under the pressure increase, hold, and decrease modes of the hydraulic braking system can be described as follows^40^:

7 $$\frac{{dP_{w} }}{dt} = \left\{ {\begin{array}{*{20}c} {\frac{{KC_{T} A_{T} }}{V}\sqrt {\frac{2}{\rho }\left( {P_{m} - P_{w} } \right)} \, increasing} \\ {0 \, holiding} \\ {\frac{{KC_{B} A_{B} }}{V}\sqrt {\frac{2}{\rho }\left( {P_{w} - P_{r} } \right)} \, decreasing} \\ \end{array} } \right.$$

In the equation, *K* and *V* are the elasticity modulus of the oil and the total volume of the wheel cylinder and oil pipe, respectively, $C_{T}$ and $C_{B}$ are the flow coefficients, $A_{T}$ and $A_{B}$ are the areas of the inlet and outlet valves, respectively, *ρ* is the density of the brake fluid, $P_{r}$ is the brake fluid chamber pressure, $P_{m}$ and $P_{w}$ are the pressures in the master cylinder and wheel cylinder, respectively.

The relationship between the hydraulic braking torque generated by the friction brake and the wheel cylinder pressure is given by^41^:

8 $$\left\{ {\begin{array}{*{20}c} {T_{hf} = 2P_{wf} \frac{{\pi D_{f}^{2} }}{4}R_{f} K_{f} } \\ {T_{hr} = 2P_{wr} \frac{{\pi D_{r}^{2} }}{4}R_{r} K_{r} } \\ \end{array} } \right.$$

In the equation, $R_{f}$ and $R_{r}$ represent the radii of the front and rear brake discs, respectively, $T_{hf}$ and $T_{hr}$ represent the braking torques of the front and rear axles, respectively, $K_{f}$ and $K_{r}$ are the braking efficiency factors of the front and rear axles, respectively, and $D_{f}^{{}}$ and $D_{r}^{{}}$ represent the diameters of the front and rear wheel cylinders, respectively. The key model parameters of the electro-hydraulic braking system are presented in Table 3.

**Table 3:** Key model parameters of the electro-hydraulic braking system.

**Parameters**	**Value**	**Unit**
Hydraulic pipeline		
Flexible hose inner diameter	3.2	mm
Flexible hose outer diameter	10	mm
Rigid pipe inner diameter	3.2	mm
Rigid pipe outer diameter	5	mm
Flexible hose length	0.4	m
Rigid pipe length	20000	N/m
Flexible hose Young’s modulus	7.8	Mpa
Rigid pipe Young’s modulus	2.06 e^5^	Mpa
Brake fluid		
Density	850	kg/m^3^
Absolute viscosity	7.17 e^−3^	kg/m/s
Bulk modulus	1700	Mpa
Brake master cylinder		
Diameter	23	mm
Brake wheel cylinder and disc		
Front brake efficiency factor	0.76	-
Rear brake efficiency factor	0.76	-
Front wheel cylinder diameter	48	mm
Front brake disc radius	96	mm
Front brake disc radius	38	mm
Rear brake disc radius	94	mm

### Control-oriented formulation of the electro-hydraulic ABS system

To bridge the physical system and the DRL controller design, we first formulate the electro-hydraulic ABS control problem in a compact, control-oriented form. This formulation explicitly shows how the agent’s actions propagate through the vehicle dynamics to affect wheel slip, and it provides a direct mapping from each physical component to the DRL elements (state, action, reward).

At each time step, after the agent outputs an action, the total braking torque on a wheel is the sum of the motor torque and the hydraulic braking torque:

9 $$T_{b,i} = T_{m,i} + T_{h,i}$$

where $T_{b,i}$ is the total braking torque, $T_{m,i}$ is the motor braking torque, and $T_{h,i}$ is the hydraulic braking torque.

According to Eq. (1), the rotational dynamics of the *i*-th wheel can be expressed as:

10 $$I_{w,i} \ddot{\omega}_{w,i} = F_{xb,i} r_{i} - T_{b,i} - T_{roll,i}$$

According to Eq. (2), the longitudinal slip ratio of wheel *i* is defined as:

11 $$\lambda_{i} = \frac{{V_{xw,i} - r_{i} \omega_{w,i} }}{\max (v,\varepsilon )}$$

where $V_{xw,i}$ is the longitudinal velocity at the wheel center, $r_{i}$ the effective rolling radius, and *\varepsilon* a small positive constant to avoid division by zero at low speeds. The longitudinal tire force $F_{xb,i}$ depends on the vertical load $F_{z,i}$ and the friction coefficient $\mu_{i}$, which is a nonlinear function of the slip ratio:

12 $$F_{xb,i} = F_{z,i} \bullet \mu_{i} \left( {\lambda_{i} } \right)$$

Equations (9)-(12) form the physical core of the ABS control problem: the agent’s actions ${a}_{t}$ directly influence the slip ratio ${\lambda}_{i}$ through the braking torque, and the slip ratio in turn determines the tire-road friction utilization.

To explicitly link the physical system to the DRL design, based on the closed-loop physical chain established above, we now explicitly map each major subsystem to its corresponding DRL elements.

1. MRBS/in-wheel motors: The motor regenerative braking torque commands serve as the continuous actions for the four wheels. The agent also observes the current motor torque and regenerative power as part of the state, enabling fast torque regulation and energy recovery.
2. HBS/solenoid valves: Instead of directly controlling valve openings, the agent selects an action from a set of predefined discrete hydraulic modes. Each mode corresponds to a specific combination of pressure increase, hold, or decrease on the front and rear axles. The wheel cylinder pressure is included in the state to inform future decisions.
3. Tire-road interface: The wheel slip ratio ${\lambda}_{i}$ is a key state variable, as it directly determines the friction utilization. The reward function includes a term that penalizes deviation of ${\lambda}_{i}$ from the optimal slip ratio ${\lambda }^{*}=0.15$, thereby guiding the agent to maintain stable braking.
4. Actuator delay dynamics: Because the MRBS responds much faster (milliseconds) than the HBS, the system violates the Markov assumption. To compensate, the state space is augmented with a sliding window of past *m* state vectors, i.e., $\overline{S}_{t} = \left[ {s_{t} ,s_{t - 1} , \ldots ,s_{t - m} } \right]$. This augmented state approximately restores Markovian properties and enables the agent to implicitly account for the heterogeneous delays.

This mapping makes transparent how the physical characteristics of each component are embedded into the DRL framework, forming the basis for the detailed controller design in Section "Design of DRL controller".

### Design of DRL controller

In DRL, the agent interacts with the environment through actions and learns iteratively via trial and error, guided by a reward signal to discover an optimal policy. The mathematical framework that describes the interaction between the agent and the environment is the Markov Decision Process (MDP). An MDP can be represented as a four-tuple of the form $\left( {S,A,P,R} \right)$. Here, *S* represents the state space, which is the set of all possible states. *A* represents the action space, which is the set of all possible actions the agent can choose from in each state. *P* is the state transition probability, which describes the probability of transitioning from the current state d to the next state *s* after the agent takes action *a*, denoted as $P\left( {s{\prime} \left| {s,a} \right.} \right)$. This probability captures the dynamic nature and uncertainty of the environment. *R* is the reward function, which defines the reward $R\left( {s,a} \right)$ the agent receives after performing action *a* in state *s*. In addition, the core assumption of MDP is the Markov property, which states that the current state and action completely determine the next state and reward, independent of the previous history. To address practical problems, the control problem needs to be formulated in the MDP framework, which requires the appropriate design of the state space, action space, and reward function.

#### Design of states and actions

The state space serves as the input to reinforcement learning and forms the basis for the agent’s decision-making process. To ensure the agent has access to sufficient and relevant information, the state space is defined as follows:

13 $$\left\{ {\begin{array}{*{20}c} {S_{V} = \left[ {\lambda ,\dot{\lambda },\omega ,v_{v} ,a_{v} } \right]^{T} } \\ {S_{E} = \left[ {A_{E} ,T_{m\max } ,P_{rec} } \right]^{T} } \\ {S_{H} = \left[ {A_{H} ,P_{w} ,\dot{P}_{w} } \right]^{T} } \\ \end{array} } \right.$$

In the equation, $S_{V}$ represents the vehicle’s dynamic state parameters, $S_{E}$ represents the motor regenerative braking state parameters, $S_{H}$ represents the HBS state parameters, *λ* is the slip ratio of each wheel, *ω* is the angular velocity of each wheel, $\ddot{x}$ is the vehicle deceleration, $\dot{x}$ is the vehicle speed, $T_{mmax}$ is the maximum torque limit based on the motor’s external characteristics, $P_{rec}$ is the MRBS energy recovery power, $P_{w}$ is the wheel cylinder pressure in the HBS, $A_{E}$ is the action space for the MRBS, and $A_{H}$ is the action space for the HBS.

**Fig. 7:**
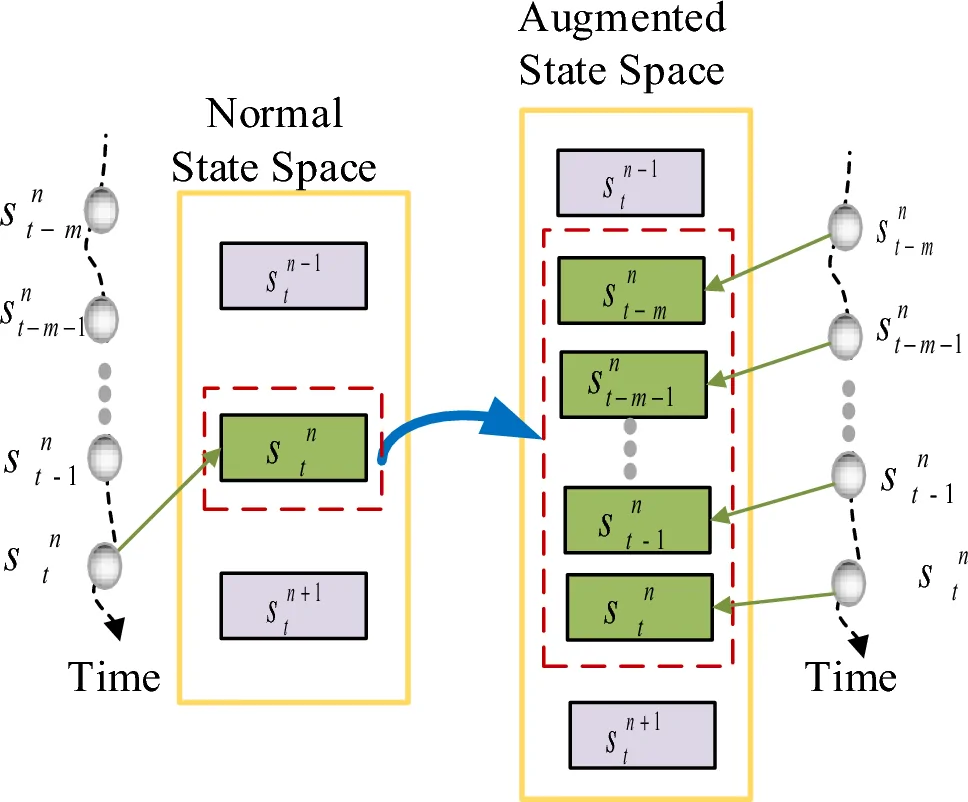
Augmented state space.

A well-defined state space and action space should satisfy the MDP, meaning that state transitions depend solely on the current state and action, and are independent of the history. However, due to the inconsistent response characteristics between MRBS and HBS, the transition to the next state is not entirely determined by the current state and action. Still, it is instead significantly influenced by previous time steps. To address this issue, an augmented state space for delay awareness is proposed, as illustrated in Fig. 7. In this framework, a sliding window method is employed to incorporate the relevant portions of the state from the past m time steps into the augmented state space.

**Fig. 8:**
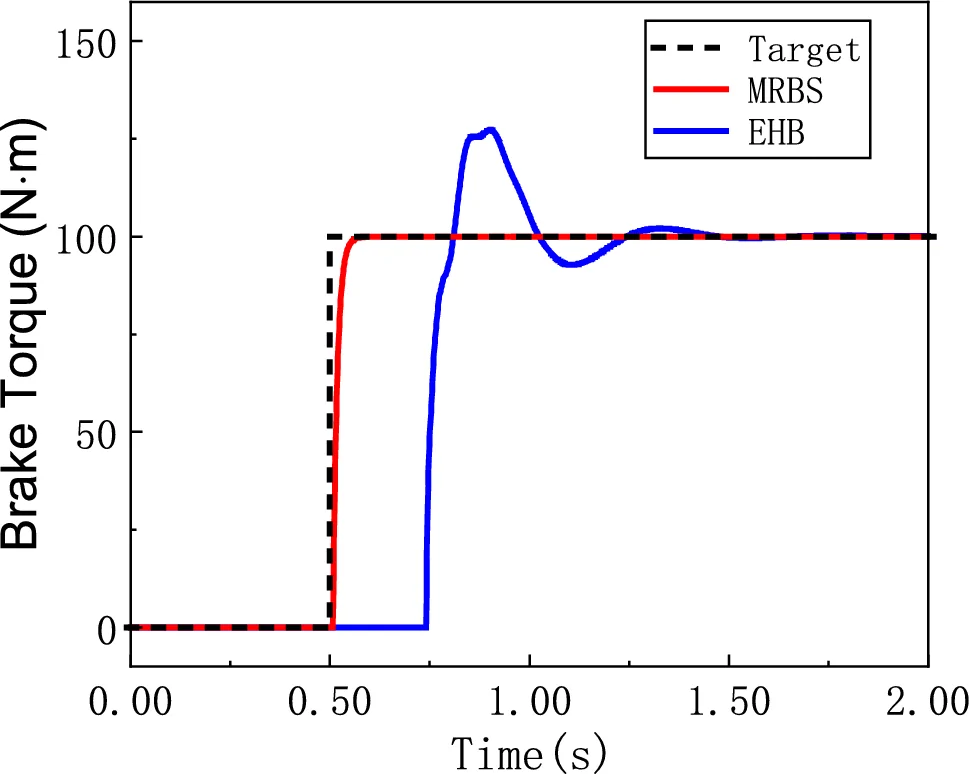
Response characteristics of MRBS and HBS on the platform.

To determine an appropriate observation window length, the response characteristics of the MRBS and HBS on the vehicle experimental platform were evaluated, as shown in Fig. 8.

The temporal span of the sliding window is jointly determined by the update cycle of the control strategy and the dynamic response characteristics of the actuators. Based on the observed response discrepancies and extensive tuning, a window length of five decision steps was ultimately selected. This configuration ensures sufficient capture of historical operational data. It also constrains dimensionality to prevent the exponential growth of the state space. As a result, it provides an appropriate observation scale for the downstream decision-making algorithm.

The final augmented state space is expressed as:

14 $$S = \left[ {S_{V} ,S_{V} \left( {t - 1} \right), \cdots ,S_{V} \left( {t - 5} \right),S_{E} ,S_{E} \left( {t - 1} \right), \cdots ,S_{E} \left( {t - 5} \right),S_{H} ,S_{H} \left( {t - 1} \right), \cdots ,S_{H} \left( {t - 5} \right),} \right]^{T}$$

In this paper, the action output of the agent is represented as:

15 $$A = \left[ {A_{E} \, A_{H} } \right]^{T}$$

In the equation, $A_{E}$ is the motor regenerative braking torque command, $A_{E} = \left[ {T_{mfl} \, T_{mfr} \, T_{mrl} T_{mrr} } \right]^{T}$, and $T_{mfl}$, $T_{mfr}$, $T_{mrl}$, $T_{mrr}$ are the regenerative braking torque commands for the left front, right front, left rear, and right rear motors, respectively.

For the action space $A_{H}$ associated with the HBS, ABS control is essentially achieved by adjusting the inlet valve opening and the outlet valve state to enable more precise pressure regulation. In this study, the solenoid valves on the same axle are assumed to operate synchronously. The opening levels available for each solenoid valve are defined as follows:

16 $$\begin{array}{*{20}c} {Inlet = \left\{ {0\% ,30\% ,100\% } \right\}} & { \, Outlet = \left\{ {0\% ,100\% } \right\}} \\ \end{array}$$

In the equation, *Inlet* is the control signal for the inlet valve, and *Outlet* is the control signal for the outlet valve.

**Fig. 9:**
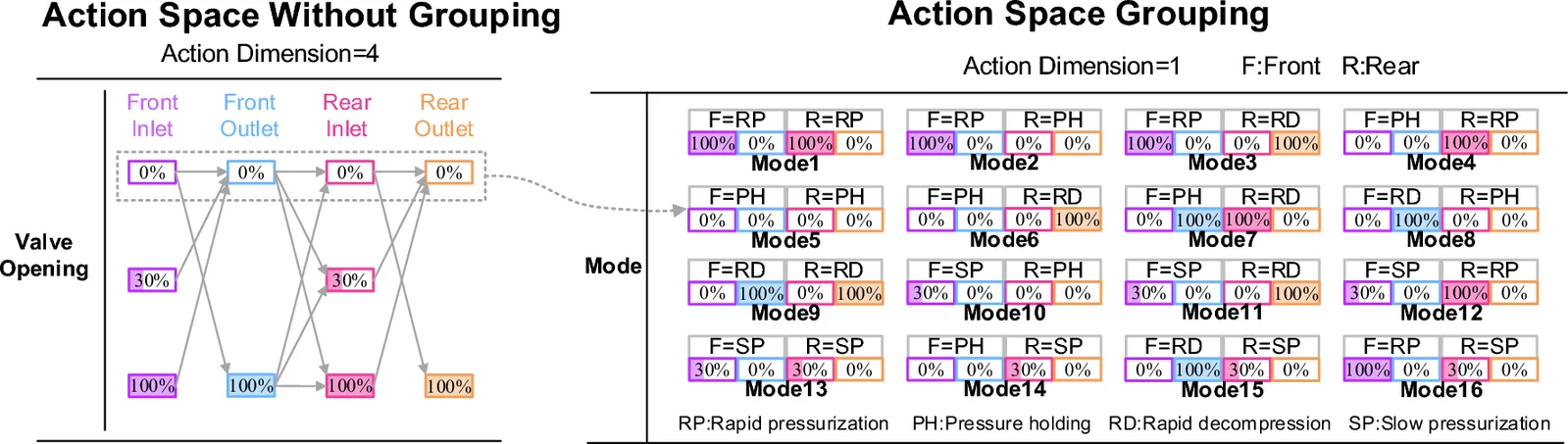
HBS action space grouping.

In this study, to simplify the action space and enhance both coordination and learning efficiency of the control policy, an action space grouping (ASG) approach is adopted, as illustrated in Fig. 9. We first define four control modes based on combinations of inlet and outlet valve actions: rapid pressure decrease (RD), pressure hold (PH), slow pressure increase (SP), and rapid pressure increase (RP). Building on this structure, the HBS action space for the agent is implemented by selecting from a predefined set of action patterns, namely:

17 $$A_{H} = mode\left( {1:16} \right)$$

These 16 modes represent different coordinated actuation strategies—for example, simultaneous rapid pressure increase on both front and rear axles, or rapid pressure increase on the front axle combined with pressure reduction on the rear axle. At each decision step, the agent simply selects one mode index from 1 to 16 as a one-dimensional action output. The system then maps this index to the corresponding action vector and transmits the control commands to the solenoid valves for execution. This design not only reduces the dimensionality of the action space and narrows the policy search space—thus accelerating training convergence—but also effectively prevents invalid or conflicting action combinations.

#### Design of rewards

The design of the reward function plays a critical role in training the agent effectively. To guide the learning process, a tailored reward function is constructed in this study, consisting of two components: a vehicle state reward and an actuation reward. On one hand, the vehicle state reward evaluates how well the objectives of integrated ABS and regenerative braking control are achieved. On the other hand, the actuation reward incorporates prior knowledge to discourage undesirable or erroneous actions, thereby improving learning stability and policy robustness.

18 $$r = r_{v} + r_{p} + r_{g}$$

In this formulation, term $r_{v}$ guides the agent to regulate the wheel slip ratio toward its optimal value, while terms $r_{p}$ and $r_{g}$ incorporate prior knowledge to discourage the agent from making inappropriate or unsafe decisions.

The formulation of term $r_{v}$ is given by Eq. (19):

19 $$\begin{array}{*{20}c} {\left\{ {\begin{array}{*{20}c} {r_{v} = r_{v\lambda f} + r_{v\lambda r} } \\ {r_{v\lambda f} = \varphi_{1} f_{v} \left( {\lambda_{f} } \right)} \\ {r_{v\lambda r} = \varphi_{2} f_{v} \left( {\lambda_{r} } \right)} \\ \end{array} } \right.} & { \, f_{v} \left( \lambda \right) = \left\{ {\begin{array}{*{20}c} {\tanh \left( { - 15 * \left| {\lambda - \Lambda_{1} } \right|} \right) \, 0 \le \lambda \le \Lambda_{1} } \\ { \, \left( {\left( {\frac{{\Lambda_{1} }}{\lambda }} \right)^{2.5} - 1} \right) \, \Lambda_{1} \le \lambda \le 1 \, } \\ \end{array} } \right.} \\ \end{array}$$

**Fig. 10:**
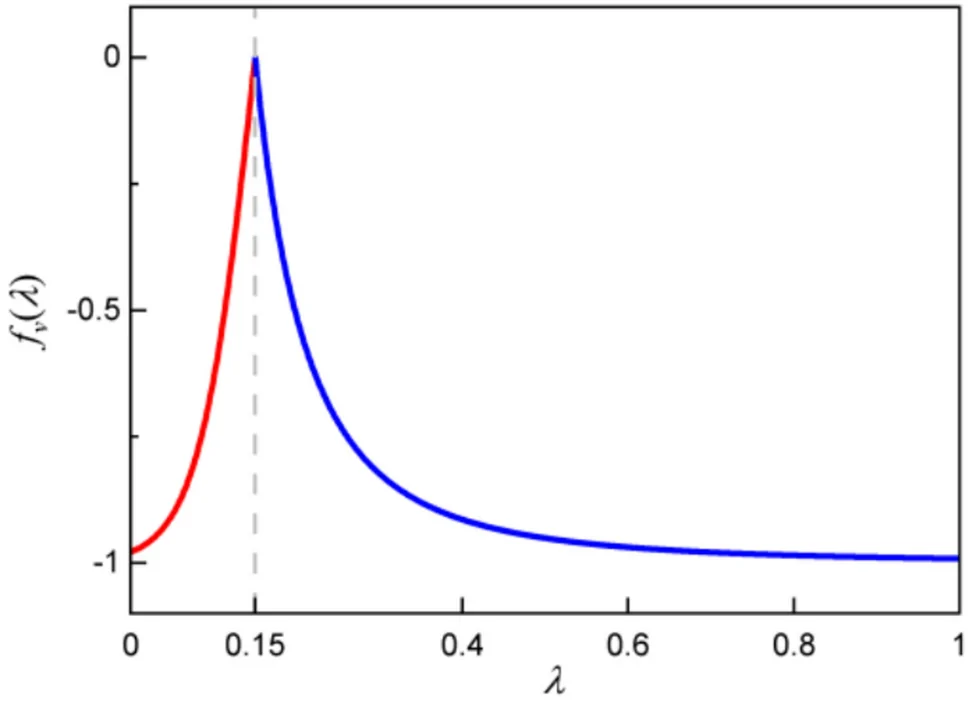
The graph of term $f_{v} \left( \lambda \right)$ in equation $r_{v}$.

In this equation, $\Lambda_{1}$ denotes the optimal slip ratio, $\lambda_{f}$ and $\lambda_{r}$ represent the slip ratios of the front and rear wheels, respectively, and $\varphi_{1,2}$ is a weighting coefficient. In various vehicle stability control systems such as ABS and traction control systems (TCS), a slip ratio of approximately 0.15 is commonly regarded as yielding optimal longitudinal traction performance^42,43^. Therefore, in this study, $\Lambda_{1} = 0.15$. The graph of term $f_{v} \left( \lambda \right)$ in Equation $r_{v}$ is shown in Fig. 10. The reward function is designed as a nearly symmetric curve centered around the axis defined by $\lambda = \Lambda_{1}$. This design serves two purposes: first, to ensure that the penalty magnitudes on either side of the axis are approximately balanced, thereby preventing the agent from developing a bias toward suboptimal actions on one side; second, to make the reward function more sensitive to deviations near the optimal slip ratio by increasing its gradient in that region, encouraging the agent to converge toward the optimal state. In addition, the range of the reward function over its domain is approximately [0,1), which facilitates subsequent weighting and integration with other components of the overall reward structure.

The reward function term $r_{p}$ is designed to discourage the agent from selecting rapid pressure decrease, pressure hold, or rapid pressure increase under inappropriate conditions. Its formulation, shown in Eq. (20), follows a piecewise function structure:

20 $$\begin{gathered} \left\{ {\begin{array}{*{20}c} {r_{p} = + r_{p\lambda f} + r_{p\lambda r} \, } \\ {r_{p\lambda f} = \left\{ {\begin{array}{*{20}c} {\varphi_{3} f_{p1} \left( {\lambda_{f} } \right) * {\rm H}\left( {p_{2} } \right) \, 0 \le \lambda_{f} \le \Lambda_{1} } \\ {\varphi_{3} f_{p2} \left( {\lambda_{f} } \right) * {\rm H}\left( {p_{1} } \right) \, \Lambda_{1} < \lambda_{f} \le 1} \\ \end{array} } \right.} \\ {r_{p\lambda r} = \left\{ {\begin{array}{*{20}c} {\varphi_{4} f_{p1} \left( {\lambda_{r} } \right) * {\rm H}\left( {p_{4} } \right) \, 0 \le \lambda_{r} \le \Lambda_{1} } \\ {\varphi_{4} f_{p2} \left( {\lambda_{r} } \right) * {\rm H}\left( {p_{3} } \right) \, \Lambda_{1} < \lambda_{r} \le 1} \\ \end{array} } \right.} \\ \end{array} } \right.\left\{ {\begin{array}{*{20}c} {f_{p1} \left( \lambda \right) = - \left( {\frac{{\lambda - \Lambda_{1} }}{{\Lambda_{1} }}} \right)^{10} \, } \\ {f_{p2} \left( \lambda \right) = - \left( {\frac{{\lambda - \Lambda_{1} }}{{1 - \Lambda_{1} }}} \right)^{4} \, } \\ \end{array} } \right. \hfill \\ \left\{ {\begin{array}{*{20}c} {{\rm H}\left( {p_{1} } \right) = \left\{ {\begin{array}{*{20}c} {1 \, if \, A_{H} = 1,2,3,4,5,6} \\ {0 \, else} \\ \end{array} } \right.} \\ {{\rm H}\left( {p_{2} } \right) = \left\{ {\begin{array}{*{20}c} {1 \, if \, A_{H} = 4,5,6,7,8,9} \\ {0 \, else} \\ \end{array} } \right.} \\ {{\rm H}\left( {p_{3} } \right) = \left\{ {\begin{array}{*{20}c} {1 \, if \, A_{H} = 1,2,4,5,7,8} \\ {0 \, else} \\ \end{array} } \right.} \\ {{\rm H}\left( {p_{4} } \right) = \left\{ {\begin{array}{*{20}c} {1 \, if \, A_{H} = 2,3,5,6,8,9} \\ {0 \, else} \\ \end{array} } \right.} \\ \end{array} } \right. \hfill \\ \end{gathered}$$

**Fig. 11:**
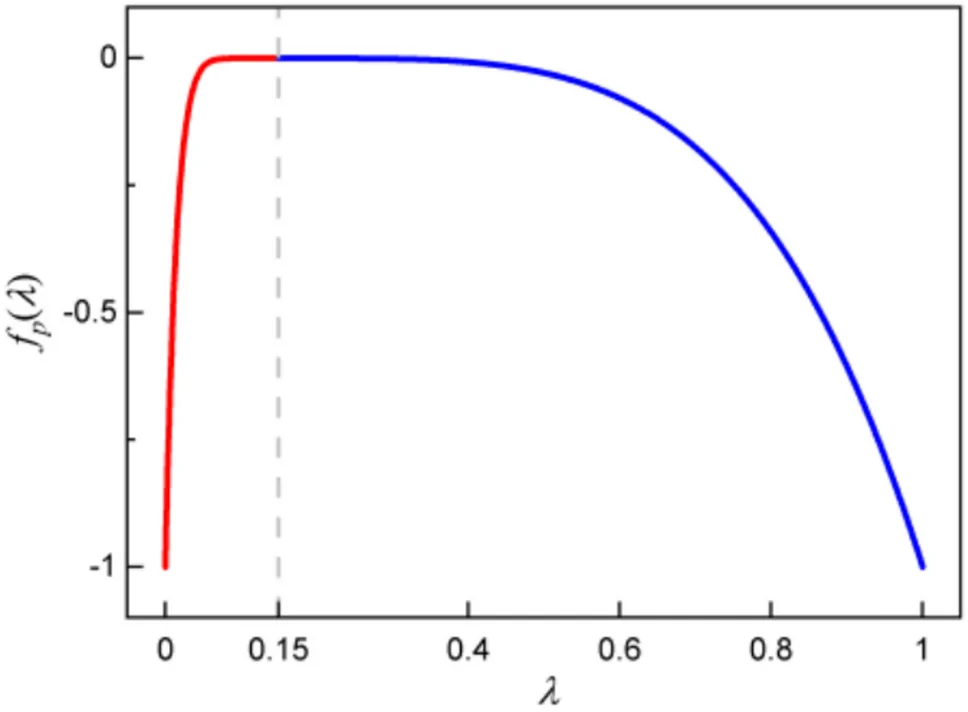
The graph of term $f_{p} \left( \lambda \right)$ in equation $r_{p}$.

In this equation,$\varphi_{3,4}$ is a weighting coefficient. ${\rm H}\left( {p_{1} } \right)$ indicates that the front wheels are in a rapid pressure increase or pressure hold state.${\rm H}\left( {p_{2} } \right)$ denotes that the front wheels are in a rapid pressure decrease or pressure hold state. ${\rm H}\left( {p_{3} } \right)$ indicates that the rear wheels are in a rapid pressure increase or pressure hold state, and ${\rm H}\left( {p_{4} } \right)$ represents that the rear wheels are in a rapid pressure decrease or pressure hold state. The graph of term $f_{p} \left( \lambda \right)$ in equation $r_{p}$ is shown in Fig. 11. It is a piecewise function defined with $\lambda = \Lambda_{1}$ as the boundary, and exhibits a convex shape: the farther the value deviates from $\Lambda_{1}$ the larger the penalty imposed; conversely, in the vicinity of $\Lambda_{1}$, the penalty is negligible. This design is grounded in prior knowledge. For the front wheels, for example, when the slip ratio is high, the HBS should avoid applying or maintaining pressure, when the slip ratio is low, it should avoid releasing or maintaining pressure. When the slip ratio is near $\Lambda_{1}$, no penalty is applied, allowing the agent to explore and learn optimal actions without undue constraint. The range of the reward function over its domain is [0,1], which simplifies subsequent adjustment of the weighting coefficient for this component.

The reward function term $r_{g}$ is designed to discourage the agent from selecting slow pressure increase actions under inappropriate conditions. Its formulation is given in Eq. (21):

21 $$\begin{aligned} \left\{ {\begin{array}{*{20}c} {r_{g} = + r_{g\lambda f} + r_{g\lambda r} \, } \\ {r_{g\lambda f} = \varphi_{5} f_{g} \left( {\lambda_{f} ,a_{v} } \right) * {\rm H}\left( {g_{1} } \right)} \\ {r_{g\lambda r} = \varphi_{6} f_{g} \left( {\lambda_{r} ,a_{v} } \right) * {\rm H}\left( {g_{2} } \right)} \\ \end{array} } \right.f_{g} \left( {\lambda ,a_{v} } \right) = & \left\{ {\begin{array}{*{20}c} { - \left( {\frac{{a_{v} }}{8}} \right)^{2} * \left( {\frac{{\lambda - \Lambda_{2} }}{{\Lambda_{2} }}} \right)^{2} \, 0 \le \lambda \le \Lambda_{2} } \\ {0 \, \Lambda_{2} < \, \lambda \le \Lambda_{1} } \\ { - \left( {\frac{{\lambda - \Lambda_{1} }}{{1 - \Lambda_{1} }}} \right)^{2} \, \Lambda_{1} < \lambda \le 1} \\ \end{array} } \right. \\ & \left\{ {\begin{array}{*{20}c} {{\rm H}\left( {g_{1} } \right) = \left\{ {\begin{array}{*{20}c} {1 \, if \, A_{H} = 10,11,12,13} \\ {0 \, else} \\ \end{array} } \right.} \\ {{\rm H}\left( {g_{2} } \right) = \left\{ {\begin{array}{*{20}c} {1 \, if \, A_{H} = 13,14,15,16} \\ {0 \, else} \\ \end{array} } \right.} \\ \end{array} } \right. \\ \end{aligned}$$

**Fig. 12:**
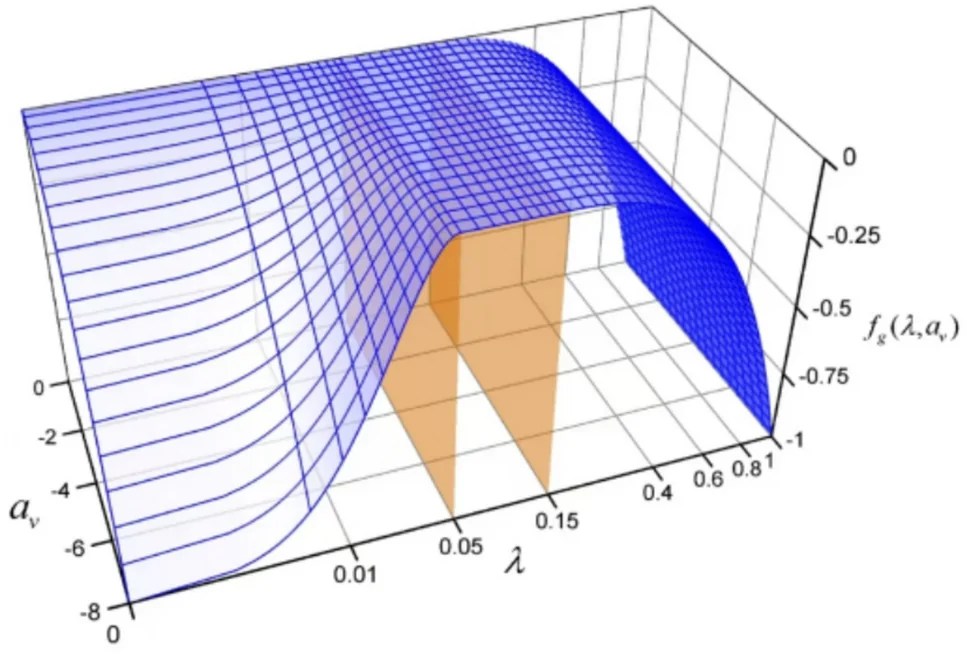
The graph of term $\, f_{g} \left( {\lambda ,a_{v} } \right)$ in equation $r_{g}$.

In this equation, $\varphi_{5,6}$ denotes a weighting coefficient, $\Lambda_{2} = 0.05$, $a_{v}$ represents the vehicle’s longitudinal acceleration. The graph of term $\, f_{g} \left( {\lambda ,a_{v} } \right)$ in equation is shown in Fig. 12. As observed when the slip ratio is very close to zero ($0 \le \lambda \le 0.05$), the vehicle’s longitudinal acceleration is introduced as a variable in the reward function. This is because acceleration can indirectly reflect road surface adhesion capability. When the longitudinal acceleration is high, it likely indicates a high-adhesion surface. In such cases, applying a slow pressure increase may prevent the slip ratio from returning quickly to its optimal value, and thus the penalty is increased. Conversely, when the longitudinal acceleration is low, indicating a low-adhesion surface, the slip ratio becomes more sensitive to the pressurization rate. In this case, a slow pressure increase is preferable to prevent wheel lock-up, and the penalty is reduced. When the slip ratio lies between near-zero and the optimal slip ratio ($0.05 < \lambda \le 0.15$), it is already close to the ideal value. Under such conditions, slow pressure increase may yield favorable results, and therefore no penalty is applied—allowing the agent to explore freely. However, when the slip ratio exceeds the optimal value ($0.15 < \lambda \le 1$), the larger it gets, the less suitable the slow pressure increase becomes, and thus the penalty increases accordingly. Additionally, the range of this reward function over its domain is normalized to [0,1], facilitating the tuning of its weight within the overall reward structure.

#### SAC and parallelization architecture

As mentioned earlier, among various DRL algorithm implementations, SAC algorithm has been widely adopted due to its strong capability to explore complex policy spaces for multi-objective optimization tasks. In SAC, the Actor component follows a policy-based approach to learn the policy π, generating actions that enable the agent to collect as much cumulative reward as possible from the environment. In contrast, the Critic component adopts a value-based approach to learn value functions, which are used to evaluate either the current state or the actions proposed by the Actor^44^.Typically, SAC involves the learning of two types of value functions:

22 $$\left\{ \begin{gathered} V(s) = E_{\pi } [G_{t} |S_{t} = s] \hfill \\ Q(s,a) = E_{\pi } [G_{t} |S_{t} = s,A_{t} = a] \hfill \\ \end{gathered} \right.$$

In the equation, the state value function *V(s)* represents the expected cumulative reward obtained by executing all subsequent actions according to policy *π* from state *s*. The state-action value function *Q(s,a)* represents the expected cumulative reward obtained by selecting action *a* from state *s* and then executing all subsequent actions according to policy *π*.

Sutton and Barto^45^ introduced the fundamental single-step Actor-Critic algorithm, with the basic process as follows:

1. Randomly initialize the Actor and Critic networks;
2. Begin an episode. In tasks with terminal states, the agent starts by outputting actions, and the entire process leading to the terminal state is considered one episode. At each time step, a single MDP trajectory, denoted as $\left\{ {s_{t} ,a_{t} ,r_{t} ,s_{t + 1} } \right\}$, is sampled from the environment. For this trajectory, the Temporal Difference (TD) error is computed by introducing the temporal difference $\delta_{t}$, as shown in the following formula:

23 $$\delta_{t} = r_{t} + \gamma V(s_{t + 1} ;\phi ) - V(s_{t} ;\phi )$$

Based on $\delta_{t}$, the objective function for updating the Critic is to minimize the mean squared error (the loss function), as shown in the following formula.

24 $$\left\{ \begin{gathered} J_{V} (\phi ) = \sum\nolimits_{t} {\delta_{t}^{2} } \hfill \\ \phi \leftarrow \phi - \lambda_{c} \nabla_{\phi } J_{V} (\phi ) \hfill \\ \end{gathered} \right.$$

The objective function for updating the Actor is to maximize the expected reward, as shown in the following formula:

25 $$\left\{ \begin{gathered} J_{V} (\phi ) = \sum\nolimits_{t} {\log \pi (a_{t} |s_{t} ;\theta )\delta_{t} } \hfill \\ \theta \leftarrow \theta + \lambda_{a} \nabla_{\theta } J_{\pi } (\theta ) \hfill \\ \end{gathered} \right.$$

3) Repeat the process in step 2 until the episode ends, then start a new episode

Building on the single-step Actor-Critic algorithm, Haarnoja, et al^46^. proposed the SAC algorithm. The main improvement of SAC is the combination of policy optimization and entropy maximization to achieve more stable and efficient policy learning. By incorporating the maximum entropy principle into the policy, SAC not only focuses on maximizing the cumulative reward but also emphasizes the exploration of the policy, thereby enhancing the robustness and generalization ability of the policy.

First, SAC formalizes the policy optimization problem as maximizing the following objective function:

26 $$\left\{ \begin{gathered} J(\pi ) = \sum\limits_{t = 0}^{T} {{\rm E}_{{(s_{t} ,a_{t} ) \sim \rho_{\pi } }} \left[ {r(s_{t} ,a_{t} ) + \alpha H(\pi ( \cdot |s_{t} ))} \right]} \hfill \\ H(\pi ( \cdot |s_{t} )) = - {\rm E}_{{a_{t} \sim \pi ( \cdot |s_{t} )}} \left[ {\log \pi (a_{t} |s_{t} ;\theta )} \right] \hfill \\ \end{gathered} \right.$$

In the equation, $H(\pi ( \cdot |s_{t} ))$ represents the entropy of the policy, and *α* is the entropy weight that controls the balance between exploration and exploitation. This objective function promotes exploration by maximizing the entropy of the policy, enabling the agent to explore the state-action space more extensively and, therefore, discover better policies.

In the Critic part, SAC uses two Q-value functions, $Q_{{\phi_{{1}} }} (s,a)$ and $Q_{{\phi_{{2}} }} (s,a)$, to estimate the value of actions. By introducing a double Q-network, SAC effectively reduces the bias in Q-value estimation and mitigates the problem of Q-value overestimation. If the bias between the two Q-values is large, it can lead to an overestimation of the model’s value, causing training divergence. The objective of the Critic network is to minimize the following objective function (loss function):

27 $$J_{Q} (\phi_{i} ) = L(\phi_{i} ) = {\rm E}_{(s,a,r,s^{\prime})} \left[ {(Q_{{\phi_{i} }} (s,a) - y)^{2} } \right]$$

In the equation, the target value *y* is calculated using the following formula:

28 $$y = r + \gamma \left( {\mathop {\min }\limits_{i = 1,2} Q_{{\overline{{\phi_{i} }} }} (s^{\prime},a^{\prime}) - \alpha \log \pi (a_{t} |s_{t} ;\theta )} \right)$$

In the equation, $\overline{\phi}_{i}$ represents the parameters of the target Q-network, and *γ* is the discount factor.

The parameters $\overline{\phi}_{i}$ of the target Q-network are updated using a soft update method, and the update formula is as follows:

29 $$\overline{\phi}_{i} \leftarrow \tau \phi_{i} + (1 - \tau )\overline{\phi}_{i}$$

In the equation, *τ* is the soft update coefficient, typically set to a small value, such as 0.01.

In the Actor part, SAC updates the policy network parameters *θ* by maximizing the following objective function:

30 $$J_{\pi } (\theta ) = {\rm E}_{s \sim D} \left[ {{\rm E}_{{a \sim \pi_{\theta } }} (\alpha \log \pi (a_{t} |s_{t} ;\theta ) - Q_{{\phi_{i} }} (s,a))} \right]$$

In the equation, *D* represents the experience replay buffer.

The experience replay buffer *D* in SAC stores the experience samples generated from the agent’s interaction with the environment. When updating the policy and value networks, the agent randomly samples experiences from the buffer. This breaks the temporal correlation between samples, reduces variance, and improves learning efficiency. Additionally, experience replay allows the agent to reuse the same experiences multiple times, enhancing data utilization.

In addition, the SAC algorithm features a mechanism for automatically adjusting the policy entropy. This mechanism dynamically adjusts the entropy weight *α* to control the magnitude of the policy entropy, thereby balancing the trade-off between exploration and exploitation. While maximizing expected rewards, it ensures that the policy’s entropy approaches a target entropy $H_{target}$. The target entropy is typically set based on the dimensionality of the action space. For example, for an action space with dimension *d*, the target entropy can be set to *- d*. The automatic adjustment mechanism fine-tunes the entropy weight by minimizing the following objective function:

31 $$J(\alpha ) = {\rm E}_{{a_{t} \sim \pi_{\theta } }} \left[ { - \alpha \log \pi (a_{t} |s_{t} ;\theta ) - \alpha H_{t\arg et} } \right]$$

The entropy weight is automatically updated using gradient descent *α*, ensuring that the actual policy entropy approaches the target entropy.

32 $$\alpha \leftarrow \alpha - \lambda_{\alpha } \nabla_{\alpha } J(\alpha )$$

In summary, the pseudocode of the SAC algorithm is shown in Table 4.

**Table 4:**
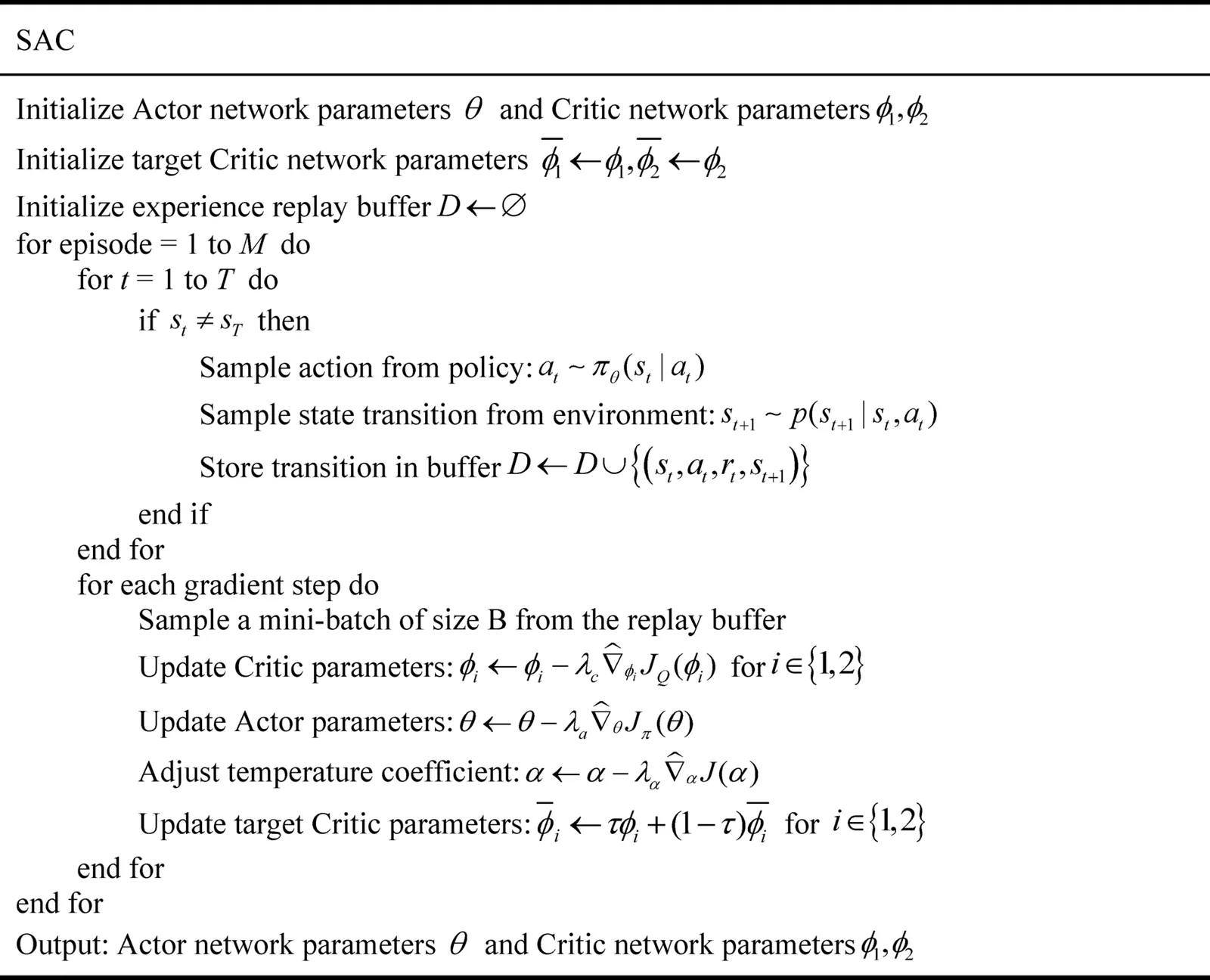
SAC Pseudocode.

**Fig. 13:**
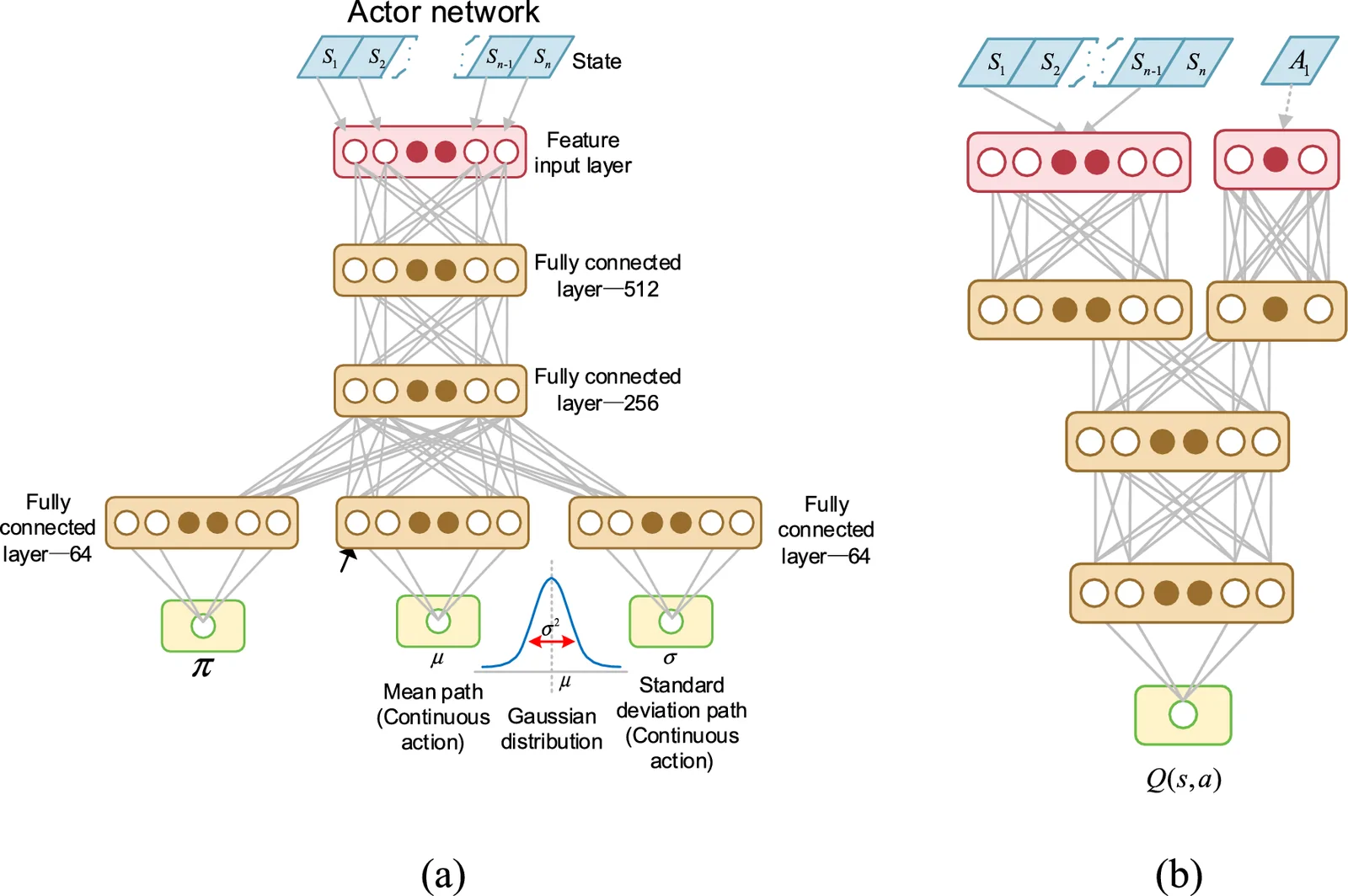
SAC network architecture. (**a**) Actor network design; (**b**) Critic network design.

In the SAC algorithm, deep neural networks (DNNs) are used to approximate both the policy and value functions. In this study, fully connected neural networks (FCNNs) are adopted for both the Actor and the Critic components. The network architecture designs for the Actor and Critic used in this work are illustrated in Fig. 13.

Reinforcement learning (RL) algorithms, agent exploration–exploitation strategies, and parallelization architectures fundamentally dictate training duration and data efficiency. Consequently, the design of the parallelization structure is pivotal for effective algorithm training and deployment. Although existing distributed deep reinforcement learning (DRL) frameworks—such as A3C and Ape-X—have demonstrated the potential of parallelization^47,48^, they were primarily engineered for game environments with discrete action spaces. Their asynchronous update mechanisms often suffer from convergence instability and policy oscillation when applied to continuous control tasks characterized by nonlinear vehicle dynamics.

**Fig. 14:**
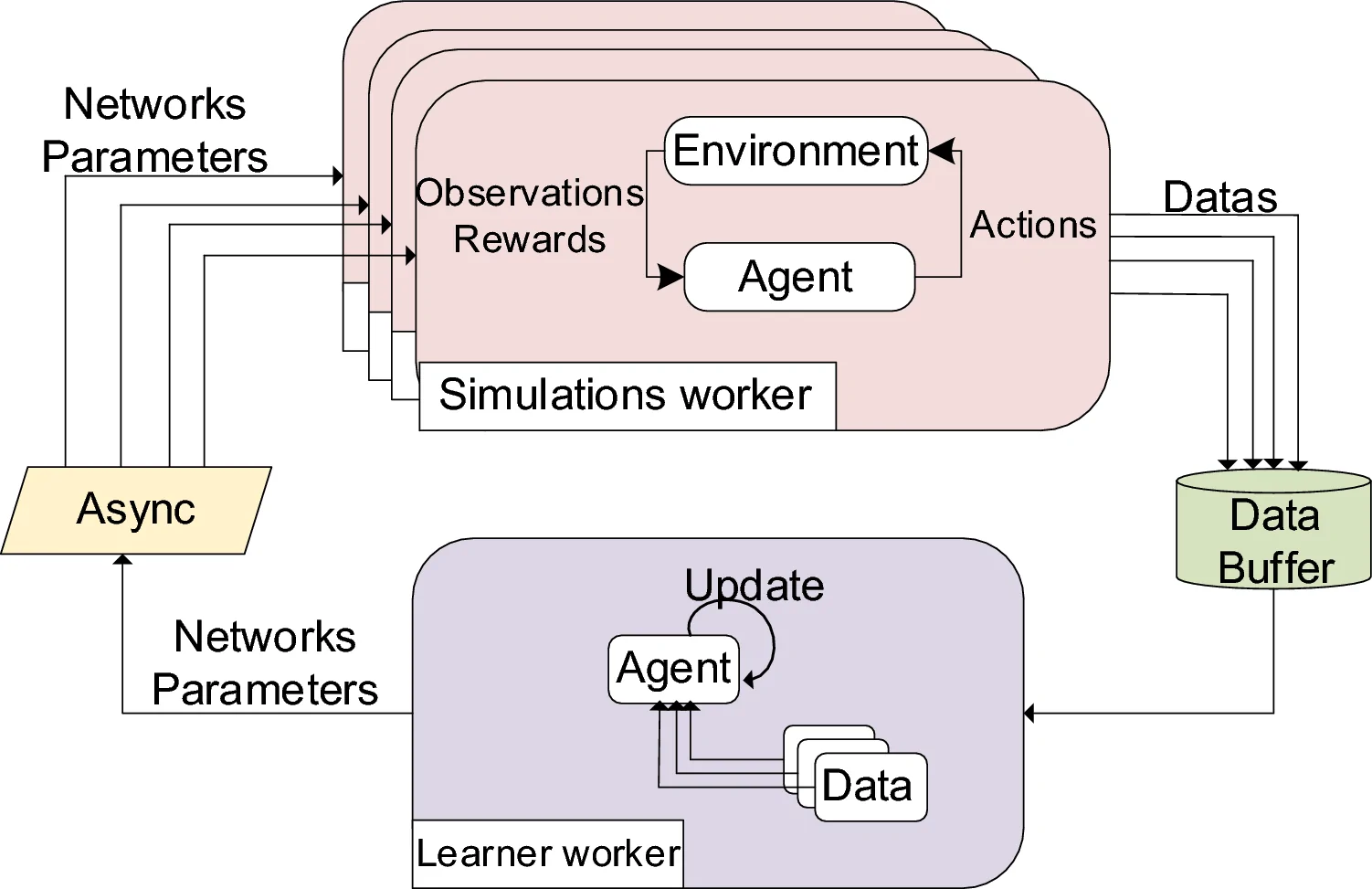
Parallelization architecture for DRL training.

To address these challenges, we leverage the off-policy nature of the SAC algorithm to propose a streamlined yet efficient architecture (Fig. 14). This framework decouples data collection from model training, ensuring that all parallel agents operate on a consistent policy version during each iteration. This synchronization minimizes gradient estimation variance and enhances training robustness in complex nonlinear systems. Distinct from cluster-based architectures reliant on network communication, our design implements a high-throughput data transmission channel via single-machine shared memory to fully exploit hardware capabilities. Furthermore, recognizing the computational intensity of high-fidelity vehicle simulation, we explicitly dedicate a greater proportion of CPU cores to environment simulation, thereby maximizing sample generation efficiency.

Figure 14 illustrates the parallelization architecture for DRL training proposed in this study, where available CPU cores are allocated to both simulation workers and learner workers. The experimental setup uses an Intel-series CPU with 12 cores, of which 11 are assigned to parallel simulation workers and 1 to a learner worker. Each simulation worker maintains an independent copy of the simulation model and the DRL agent parameters, while the learner worker holds its copy of the DRL agent parameters, including those used by the SAC algorithm and associated neural networks. During each training iteration, the 11 simulation workers run the vehicle–environment interaction models in parallel, collecting states, actions, rewards, and the current policy parameters, which are then transmitted to a data buffer. The learner worker continuously samples from the data buffer to compute gradients and optimize the neural network parameters.

### Closed-loop stability and safety considerations

The closed-loop stability of the proposed DRL-based ABS controller is first ensured by the global boundedness imposed by physical constraints. All state variables and control inputs in the system are inherently limited by their respective physical boundaries, guaranteeing that the closed-loop trajectory never leaves the feasible region.

As defined in Eq. (11) of Section "Control-oriented formulation of the electro-hydraulic ABS system", the wheel slip ratio $\lambda_{i}$ is calculated with a lower-speed protection, and its value naturally satisfies $0 \le \lambda_{i} \le 1$, where $\lambda_{i} = 0$ corresponds to free rolling and $\lambda_{i} = 1$ to full lock. The vehicle speed *v* and wheel angular velocities $\omega_{i}$ are bounded by the initial vehicle speed and the energy dissipation during braking, remaining non-negative finite variables.

On the control input side, the motor regenerative braking torque $T_{m,i}$ is limited by the motor’s external characteristics, satisfying:

33 $$0 \le T_{m,i} \le T_{\max ,i}^{m} \left( {\omega_{i} } \right)$$

The hydraulic braking torque $T_{h,i}$ is limited by the wheel cylinder pressure $P_{w,i}$, which itself is constrained by the physical limits of the hydraulic system:

34 $$0 \le P_{w,i} \le P_{\max }$$

Furthermore, the hydraulic mode action $A_{H} = mode\left( {1:16} \right)$ is selected from a finite discrete set, each mode corresponding to a permitted combination of pressure increase, hold, or decrease, thereby avoiding hazardous conditions such as simultaneous full opening of both inlet and outlet valves that would short-circuit the master cylinder to the reservoir.

Actuator saturation (torque limits, pressure boundaries) together with the finite action set constitute a safety barrier for the system. Consequently, even in the presence of modeling uncertainties and external disturbances, all states of the closed-loop system remain bounded and never diverge to physically infeasible regions.

## Results and discussion

All training processes were conducted on a computer equipped with 64 GB RAM and a 13th Gen Intel® Core™ i9-13900K processor. The implementation was based on MATLAB’s Reinforcement Learning Toolbox, and the vehicle model was developed using AMESim. To evaluate the effectiveness of the proposed control strategy for ABS and the benefits of the introduced enhancements, this study compares four approaches:

1. SAC-DA-ASG: SAC with both action space grouping (ASG) and delay awareness (DA);
2. SAC-DA: SAC with DA but without ASG;
3. SAC-ASG: SAC with ASG but without DA;
4. Bosch control algorithm: which is a widely adoptedΔ and industry-proven method.

**Fig. 15:**
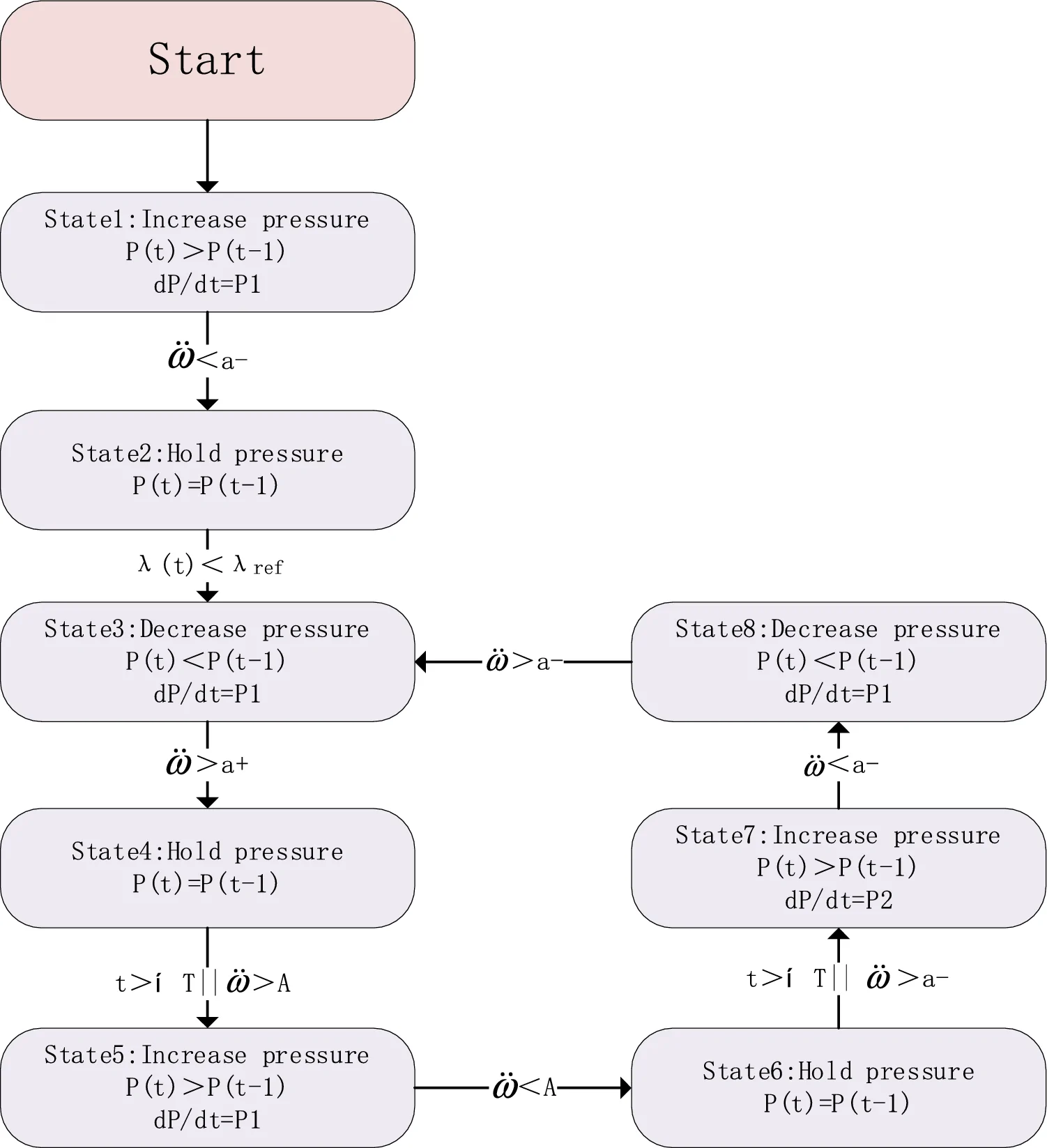
Flowchart of core of Bosch algorithm.

The purpose of this comparison is to validate the performance of SAC-DA-ASG in achieving integrated ABS control for electro-hydraulic composite braking, as well as to assess the individual contributions of ASG and DA to training performance and efficiency. The Bosch control algorithm represents a conventional control strategy in which MRBS is deactivated when ABS is engaged, thereby losing the benefits of MRBS’s rapid response and energy recuperation, has been introduced in prior studies^31,49^. Figure 15 illustrates the Bosch control flow, and Table 5 lists its control parameters. For all SAC-based comparison strategies, the neural network architecture and hyperparameter configurations were kept consistent. The detailed algorithm hyperparameters are provided in Table 6.

**Table 5:** Bosch algorithm parameters.

Front	Value	Rear	Value
*a-(rad/s* ^*2*^ *)*	−220	*a-(rad/s* ^*2*^ *)*	−175
*a+(rad/s* ^*2*^ *)*	80	*a+(rad/s* ^*2*^ *)*	50
*A(rad/s* ^*2*^ *)*	500	*A(rad/s* ^*2*^ *)*	500
*ΔT(ms)*	50	*Δ* *T(m)*	50
*P1(bar/s)*	600	*P1(bar/)*	600
*P2(bar/s)*	60	*P2(bar/s)*	60
λref	0.15	λref	0.15

**Table 6:** SAC hyperparameter settings.

**Hyperparameter**	**Value**
MiniBatchSize	256
DiscountFactor	0.95
LearnRate of Actor	1e-4
LearnRate of Critic	2e-4
ExperienceBufferLength	3e6
LearnRate of EntropyWeight	1e-4
TargetEntropy	−2
TargetSmoothFactor	2e-3
GradientThreshold	5

## Iterative training results

**Fig. 16:**
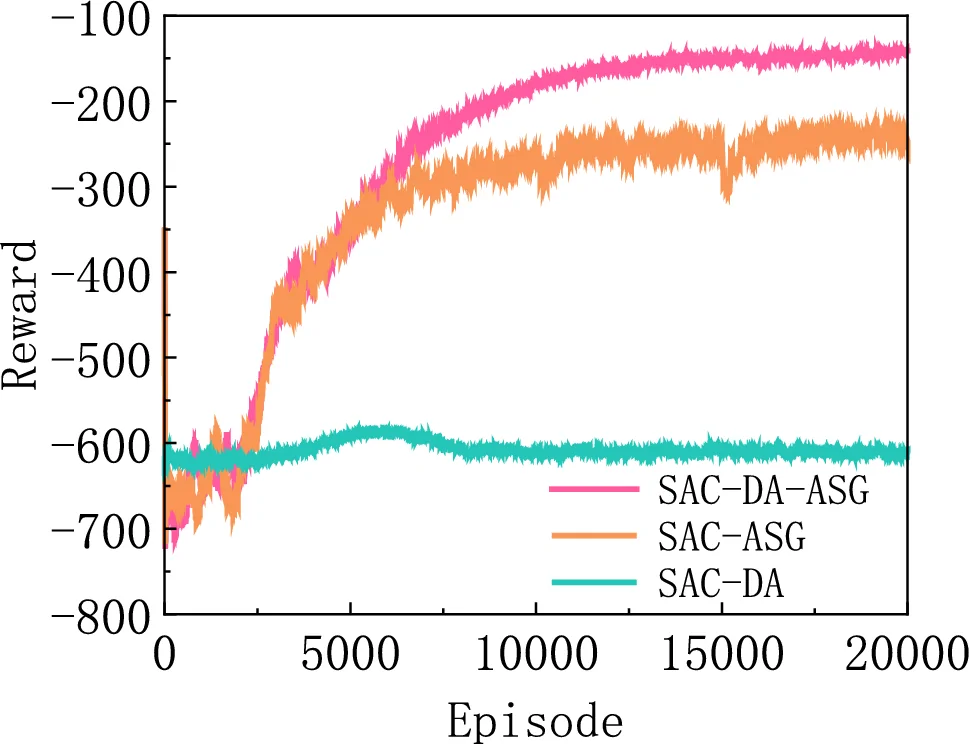
Training process.

To enhance the generalization capability of the learned policy, it is essential to incorporate random variations in environmental conditions during training. Since wheel lock-up events predominantly occur under emergency braking scenarios, each training episode in this study simulates an emergency braking condition initiated at an initial vehicle velocity of 100 km/h with maximum braking intensity. The road surface friction coefficient is randomly sampled within a predefined range, and the optimal slip ratio is set to 0.15. Figure 16 presents the learning curves observed during training, where the horizontal axis represents training episodes and the vertical axis indicates the cumulative reward obtained by the policy. As shown in Fig. 16, the proposed ASG method significantly improves training performance. The agent without ASG fails to achieve convergence, whereas the agent incorporating ASG exhibits stable learning progress. Furthermore, integrating DA on top of ASG leads to additional improvements in training efficiency and convergence behavior.

**Fig. 17:**
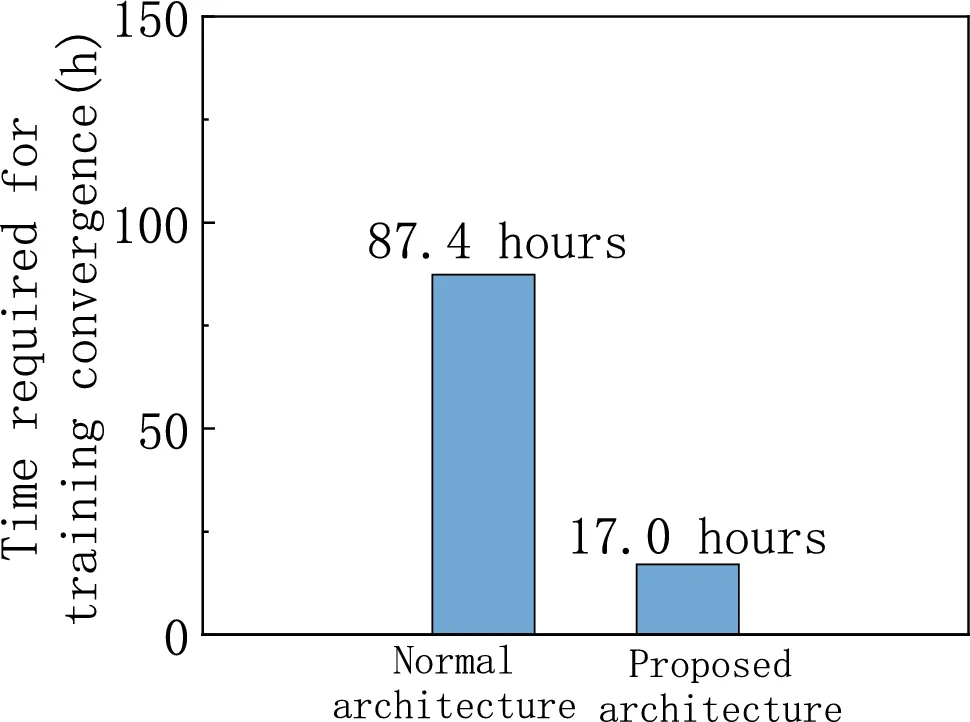
Training convergence time.

We also evaluate the impact of the parallelization architecture for DRL training on training efficiency under the SAC-DA-ASG framework. As shown in Fig. 17, with all other parameters held constant, the use of the parallelization architecture results in an approximately 5.14 × improvement in training convergence speed.

### Validation of control policy effectiveness

To thoroughly validate the effectiveness and generalization capability of the proposed control strategy, the trained agent is integrated into the control framework and tested under simulation conditions with different road surface adhesion levels: low (0.2), medium (0.5), and high (0.7). In most existing studies and engineering applications, ABS control is typically deactivated at vehicle speeds below 15 km/h^50,51^. Therefore, the results in this study focus exclusively on the braking process from an initial speed of 100 km/h down to 15 km/h. To validate the effectiveness of the proposed control strategy and to assess the benefits of coordinated MRBS and HBS control within ABS for in-wheel motor-driven electric vehicles with electro-hydraulic composite braking, this study compares three control approaches: SAC-DA-ASG, SAC-ASG, and the widely adopted Bosch control algorithm. As discussed earlier, the SAC-DA method without action space grouping failed to achieve convergence during training and is therefore excluded from the comparison due to its lack of practical applicability.

**Fig. 18:**
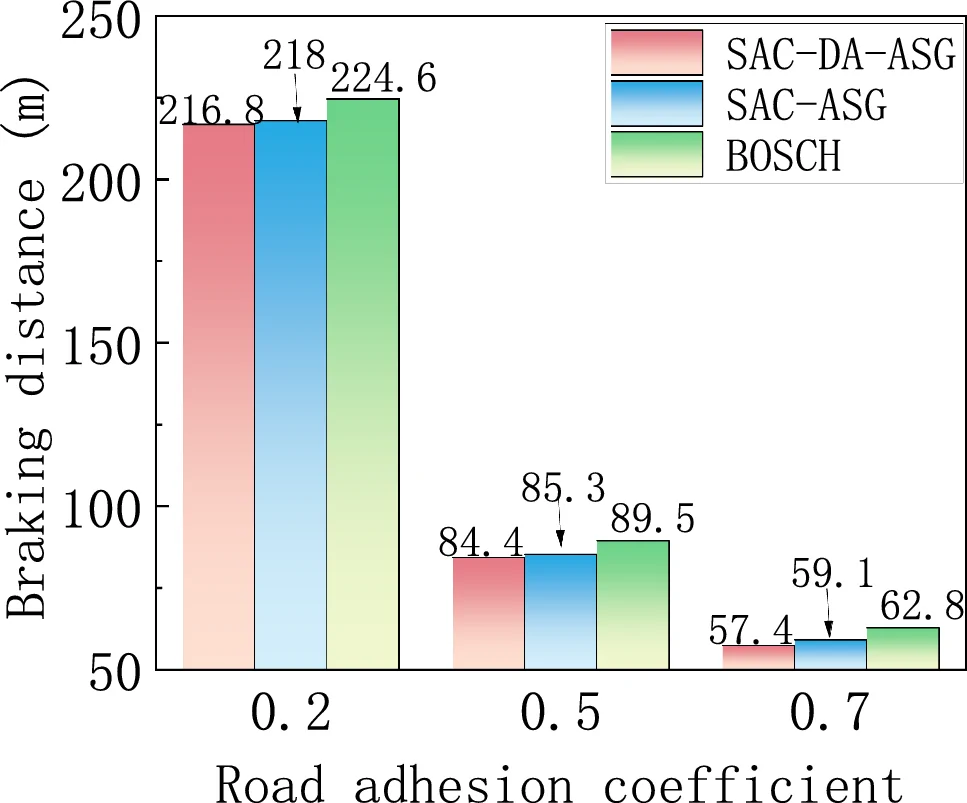
Braking distances under different road adhesion coefficients.

**Fig. 19:**
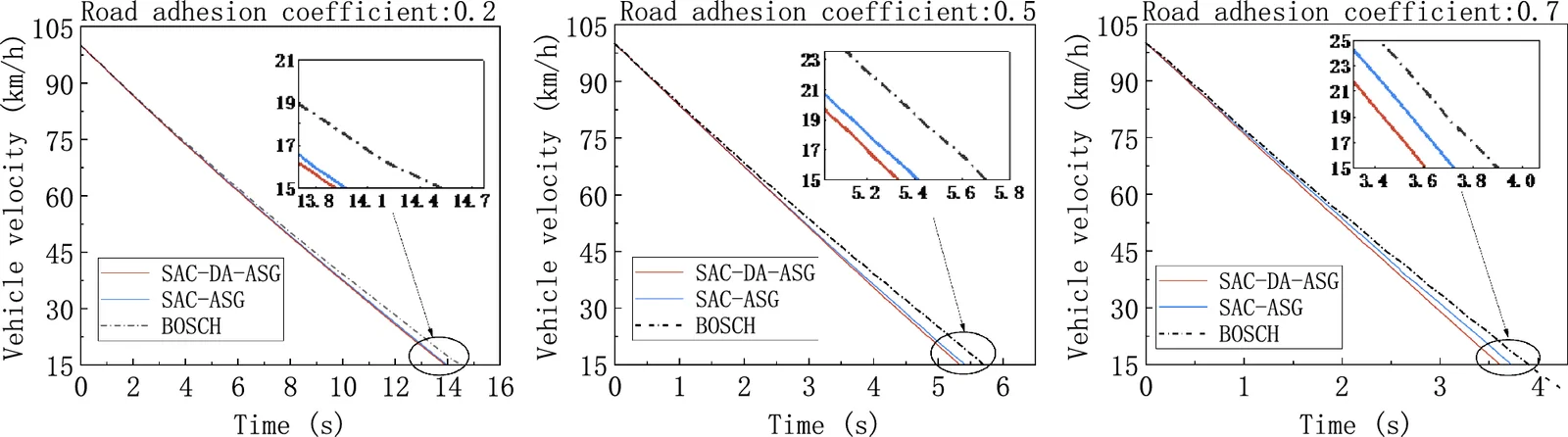
Vehicle velocity under different road adhesion coefficients.

Figures 18 and 19 show the braking distances and vehicle velocity profiles, respectively, under different road surface adhesion conditions using the compared control strategies. Compared to the Bosch controller, the proposed strategy consistently achieves shorter braking times and distances across all adhesion levels. Furthermore, relative to the SAC-ASG approach without delay awareness, incorporating delay awareness leads to additional improvements in braking performance. The specific quantitative gains for both comparisons are summarized in Table 7.

**Table 7:** Performance gains of the proposed method compared to the Bosch controller.

Road adhesion coefficients	Control Strategy	Braking Distance (m)	Dist. Reduction vs. Bosch	Braking time (s)	Time. Reduction vs. Bosch
0.2	SAC-DA-ASG	216.81	↓3.48%	13.93	↓4.20%
	SAC- ASG	218.03	↓2.93%	13.98	↓3.85%
	Bosch	224.62	—	14.54	—
*0.5*	SAC-DA-ASG	84.42	↓5.71%	5.34	↓6.32%
	SAC- ASG	85.29	↓4.74%	5.42	↓4.91%
	Bosch	89.53	—	5.70	—
*0.7*	SAC-DA-ASG	57.41	↓8.61%	3.62	↓7.65%
	SAC- ASG	59.10	↓5.92%	3.74	↓4.59%
	Bosch	62.82	—	3.92	—

**Fig. 20:**
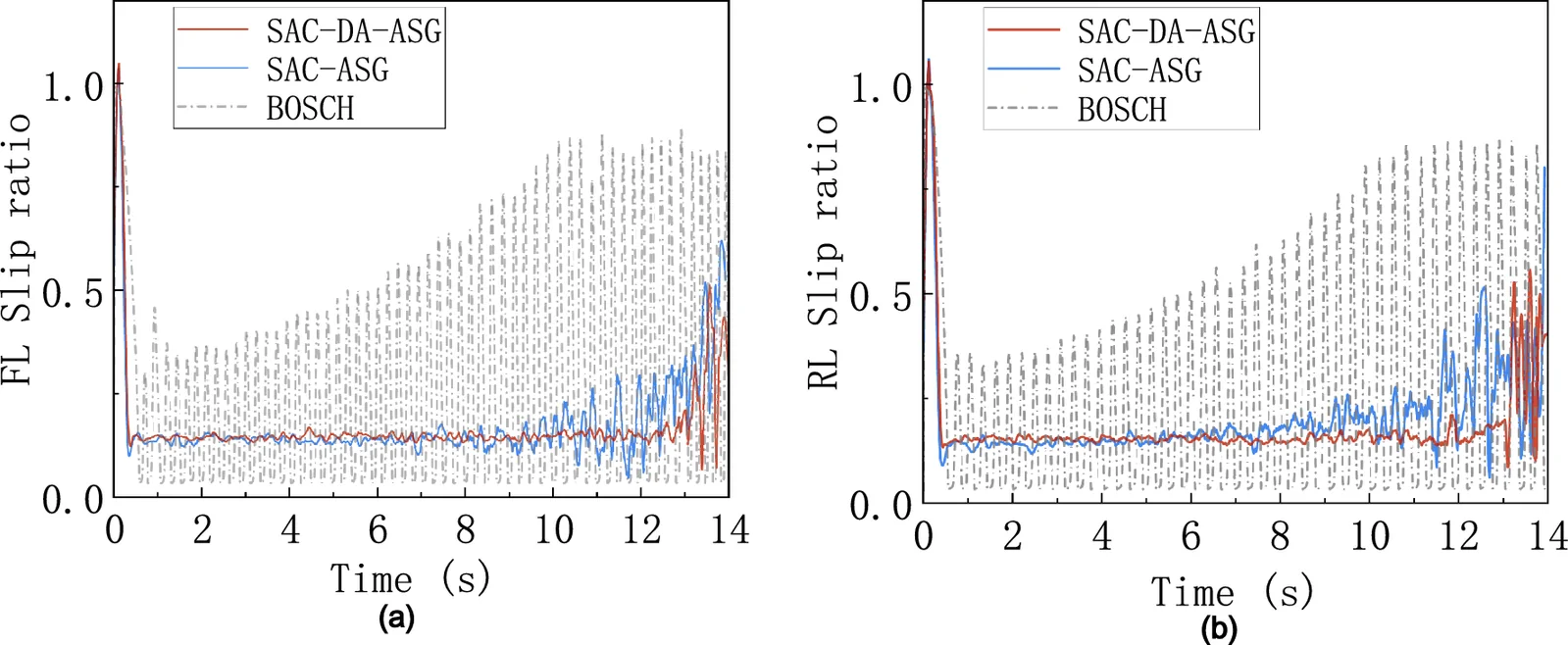
Slip ratio under different control strategies with a road adhesion coefficient of 0.2. (**a**)FL wheel slip ratio. (**b**)RL wheel slip ratio.

**Fig. 21:**
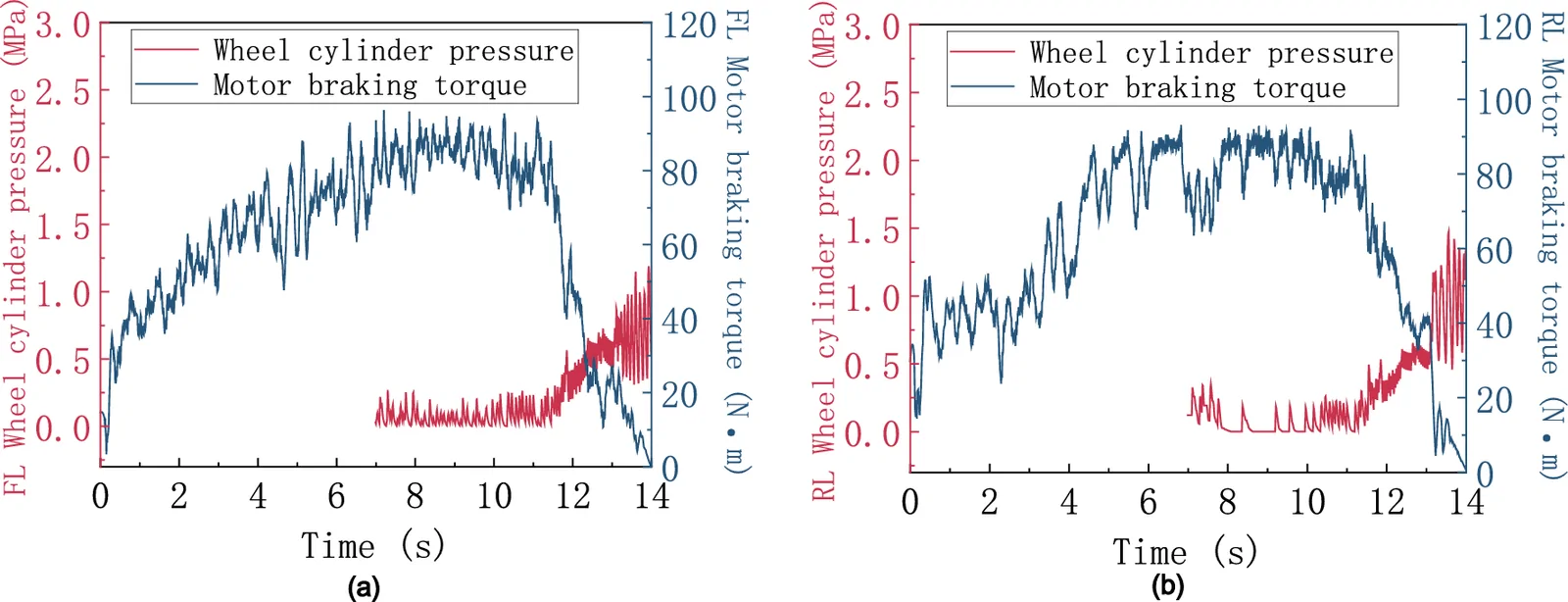
Braking actuation under SAC-DA-ASG with a road adhesion coefficient of 0.7.(**a**) FL MRBS braking torque and HBS cylinder pressure.(**b**) RL MRBS braking torque and HBS cylinder pressure.

**Fig. 22:**
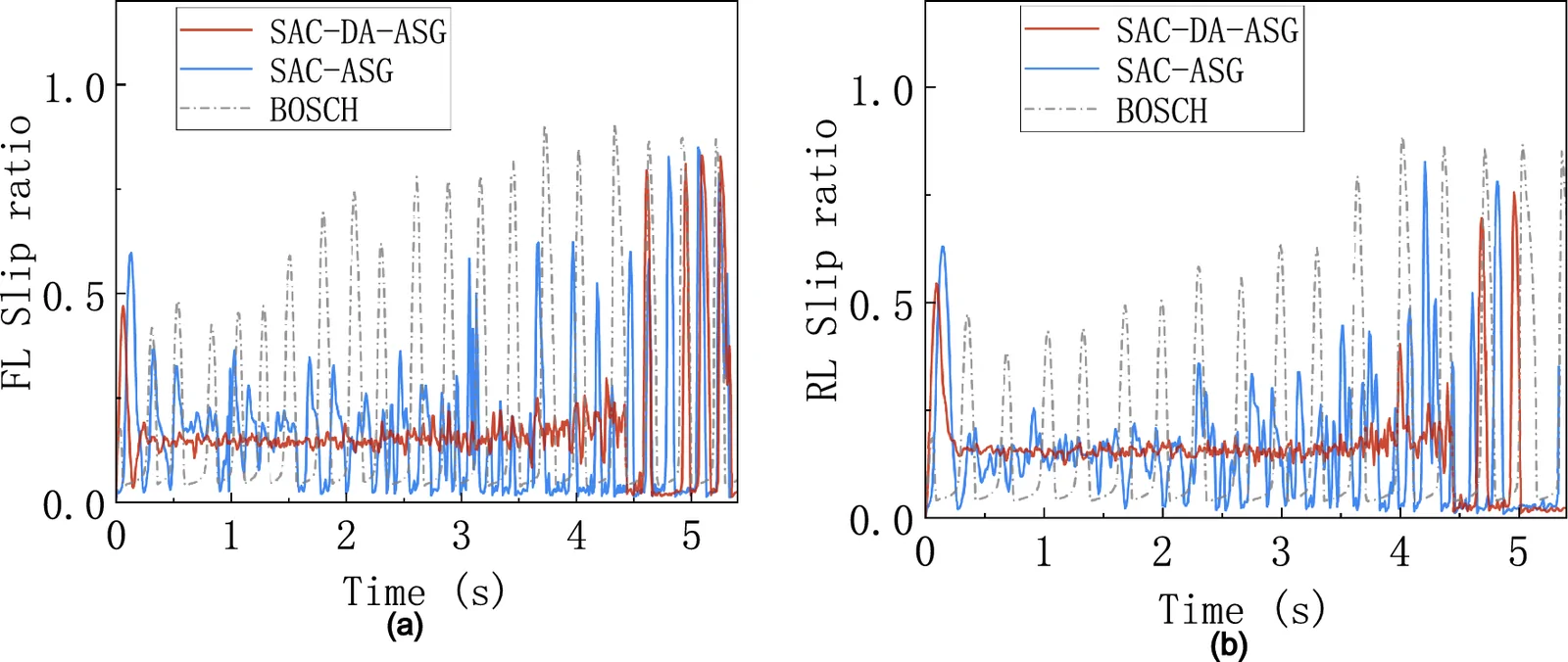
Slip ratio under different control strategies with a road adhesion coefficient of 0.5. (**a**)FL wheel slip ratio. (**b**)RL wheel slip ratio.

**Fig. 23:**
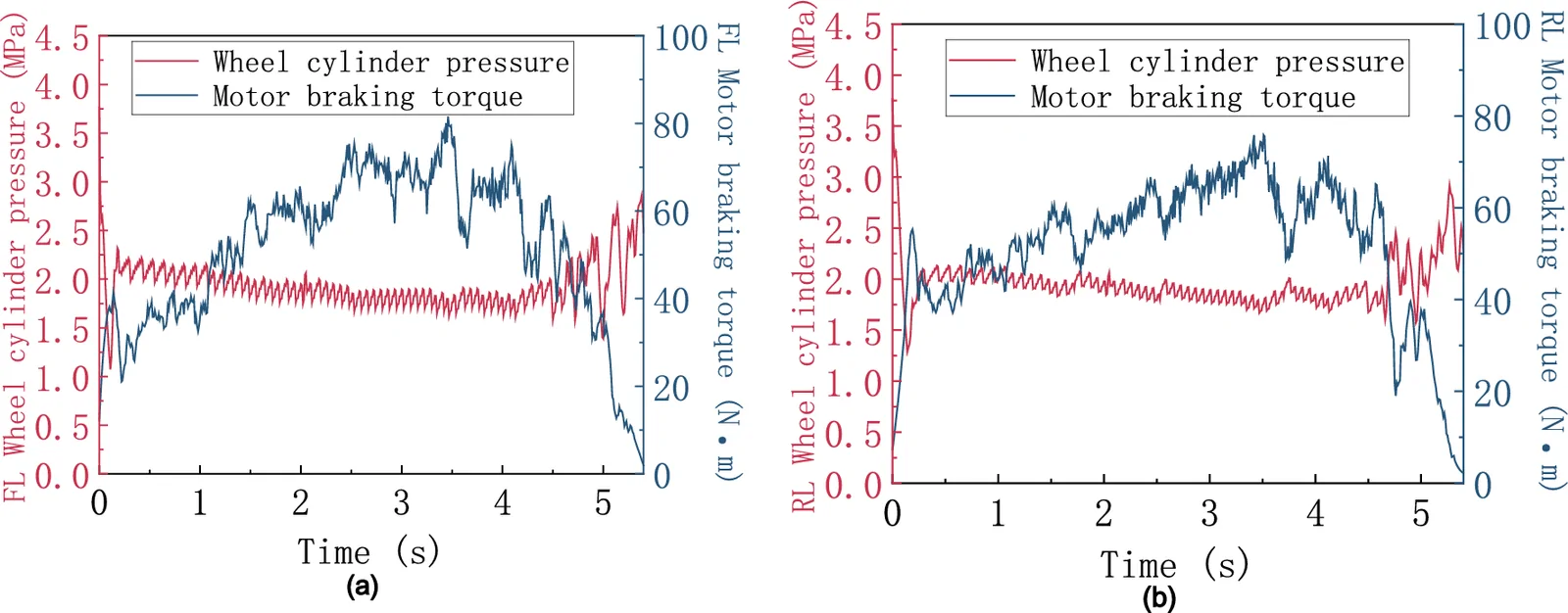
Braking actuation under SAC-DA-ASG with a road adhesion coefficient of 0.5. (**a**) FL MRBS braking torque and HBS cylinder pressure. (**b**) RL MRBS braking torque and HBS cylinder pressure.

**Fig. 24:**
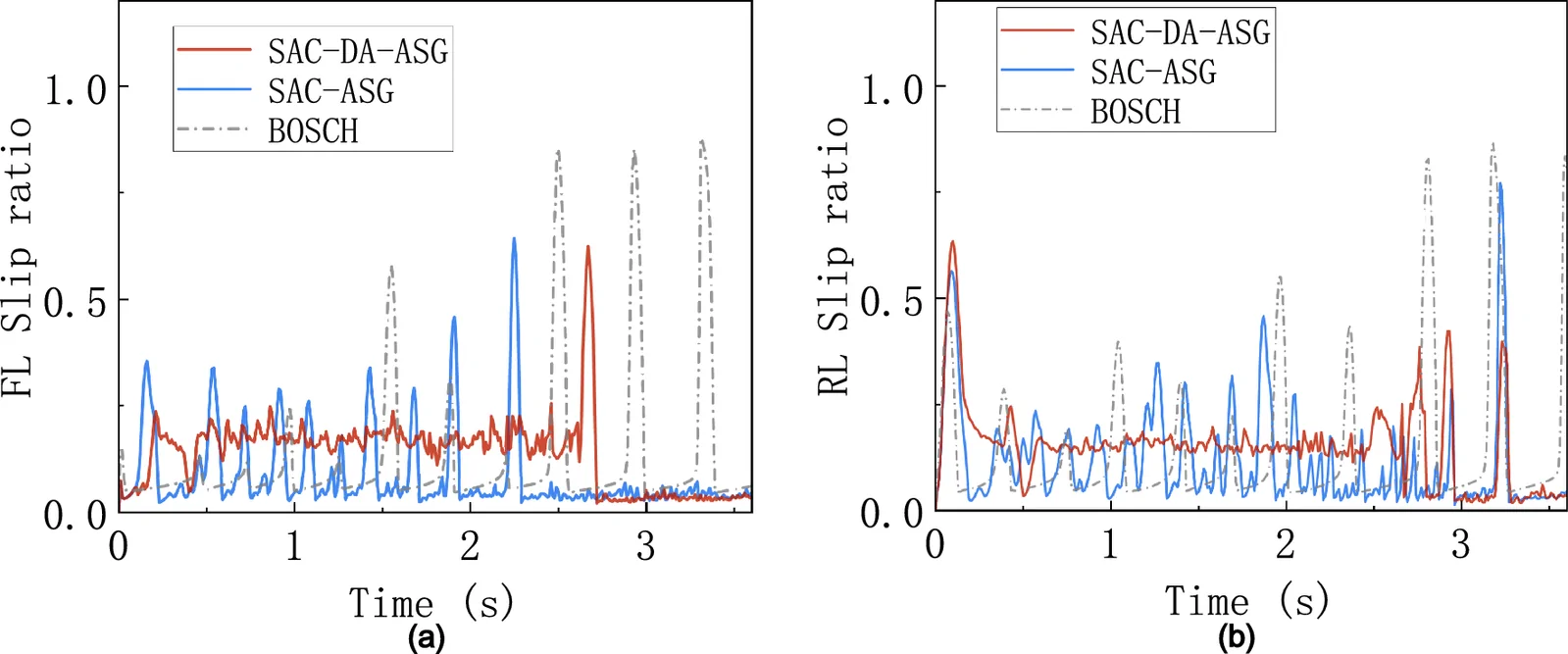
Slip ratio under different control strategies with a road adhesion coefficient of 0.7. (**a**) FL wheel slip ratio. (**b**)RL wheel slip ratio.

**Fig. 25:**
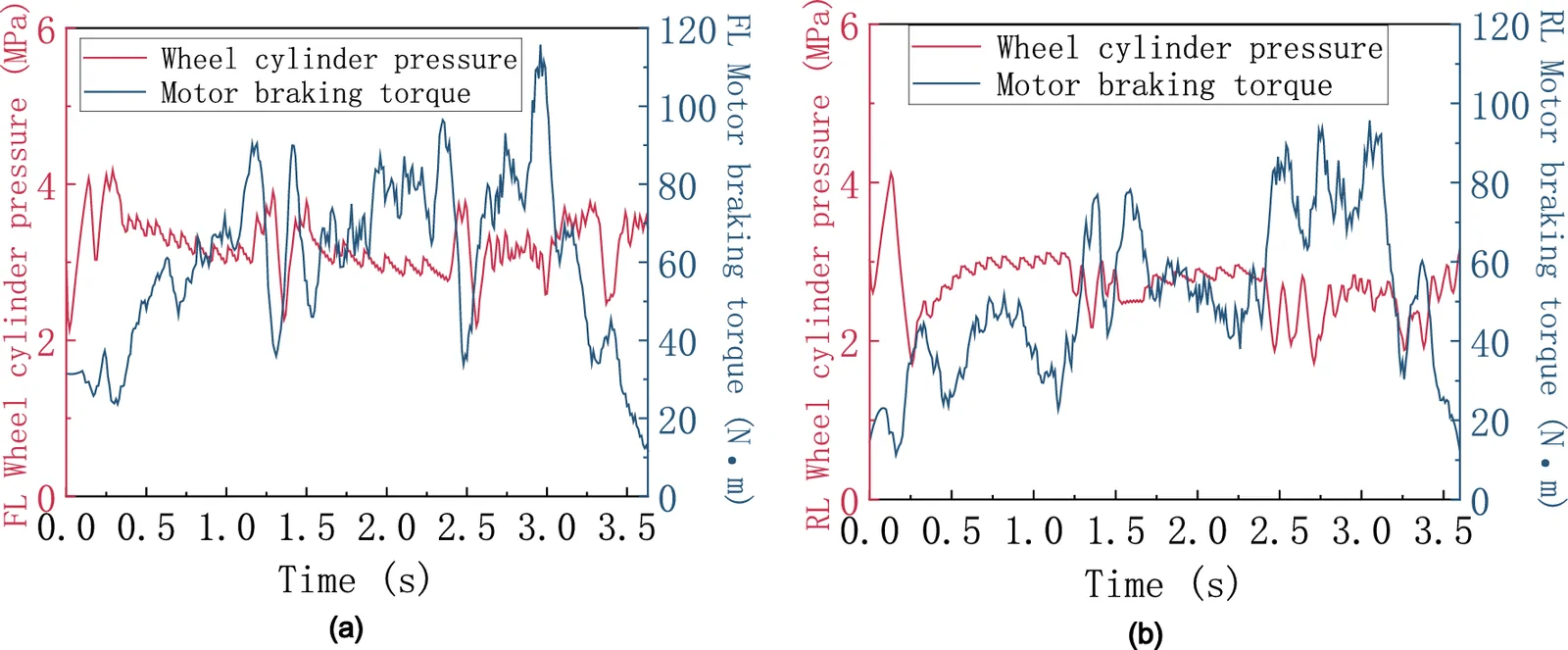
Braking actuation under SAC-DA-ASG with a road adhesion coefficient of 0.5.(**a**) FL MRBS braking torque and HBS cylinder pressure. (**b**) RL MRBS braking torque and HBS cylinder pressure.

Figures 20,21,22,23,24,25 present the braking performance (slip ratio, MRBS torque, and HBS pressure) under varying road adhesion coefficients (0.2,0.5,0.7). Across all conditions, the proposed SAC-DA-ASG strategy demonstrates rapid activation (within 0.35–0.4 s) and superior slip regulation compared to SAC-ASG and Bosch benchmarks. Specifically, it maintains the slip ratio closer to the optimal value of 0.15 with significantly reduced fluctuations.

Under low adhesion (*μ =0.2*, Figs. 20), the strategy yields the most substantial gains, reducing the peak slip ratio of the left front wheel by 0.11 and 0.37 compared to SAC-ASG and Bosch, respectively. As adhesion increases to *μ =0.5* (Fig. 22) and *μ =0.7* (Fig. 24), the relative improvement in peak slip reduction remains consistent but with smaller absolute margins (e.g., reductions of 0.03/0.08 for *μ =0.5* and 0.02/0.26 for *μ =0.7* on the front wheel), reflecting the higher friction availability that benefits all controllers.

A brief slip ratio fluctuation is consistently observed in the late braking stage across all scenarios (visible in the slip profiles of Figs 20, 22, and 24). To investigate the cause of this phenomenon, we analyzed the corresponding actuation behaviors of the MRBS and HBS shown in Figs. 21, 23 and 25. These figures reveal that toward the end of braking, the motor braking torque drops rapidly in all cases. This behavior is attributed to the continuous reduction in vehicle velocity, which limits the maximum torque output due to the motor’s external characteristics. Consequently, the MRBS gradually disengages, leading to the transient slip variations observed in the slip ratio profiles. Despite this physical limitation, the coordinated control ensures stable braking performance throughout the maneuver.

In addition, braking energy recuperation is also an important aspect to consider. The regenerative braking energy recovery rate, denoted as *η*, can be expressed as:

35 $$\eta = \frac{{E_{q} }}{{E_{b} }} \times 100\%$$

The actual energy recovered by the traction battery is:

36 $$E_{q} = \int {P_{b} dt}$$

where $P_{b}$ represents the charging power of the traction battery.

The total recoverable energy during the braking process is:

37 $$E_{b} = \frac{1}{2}m(v_{1}^{2} - v_{2}^{2} )$$

where $v_{1}$ is the initial braking speed of the vehicle and $v_{2}$ is the final braking speed.

**Fig. 26:**
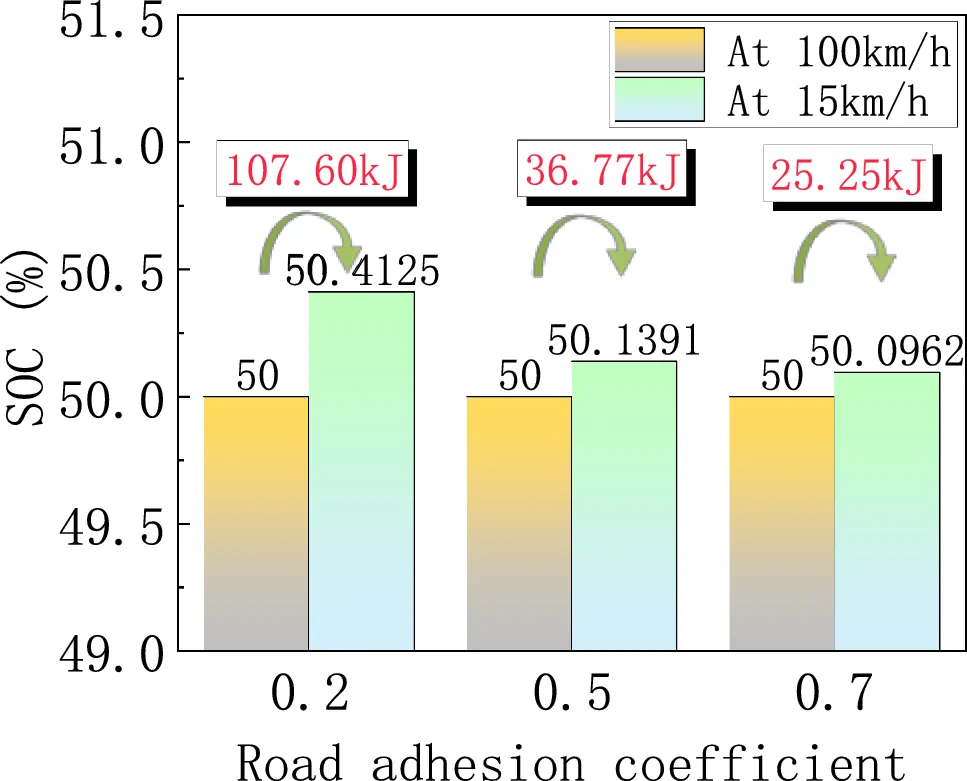
Energy recovery under the SAC-DA-ASG strategy for different road adhesion coefficients.

Figure 26 and Table 8 present the regenerative braking energy recovery results under different road adhesion conditions using the proposed SAC-DA-ASG control strategy, along with a comparative analysis. Under a road adhesion coefficient of 0.2, the regenerative braking system recovered 107.60 kJ, achieving an energy recovery rate of 35.67%. Under a road adhesion coefficient of 0.5, the recovered energy was 36.77 kJ with a recovery rate of 12.19%, and under a road adhesion coefficient of 0.7, the system recovered 25.25 kJ, corresponding to a recovery rate of 8.37%. Compared to the SAC-ASG strategy, the SAC-DA-ASG strategy demonstrates a significant improvement in energy recovery performance across all tested road adhesion levels.

**Table 8:** Comparison of energy recovery performance.

Road adhesion coefficient	Strategy	Energy recovery/kJ	
*0.2*	SAC-DA-ASG	107.60	35.67%
	SAC- ASG	99.03	32.82%
*0.5*	SAC-DA-ASG	36.77	12.19%
	SAC- ASG	29.21	9.68%
*0.7*	SAC-DA-ASG	25.25	8.37%
	SAC- ASG	19.03	6.31%

### Policy sensitivity analysis and robustness verification

To comprehensively evaluate the proposed control strategy, we conducted rigorous sensitivity analyses and robustness validations across two dimensions: internal structural parameters and external environmental uncertainties. First, in Section "Sensitivity Analysis of ASG Grouping Modes", we examine the algorithm’s sensitivity to the number of ASG grouping modes. Subsequently, we validate the strategy’s robustness against critical real-world challenges, specifically assessing its performance under split- *μ* road conditions (Section "Performance under split- road conditions"), resilience to time-varying actuator delays (Section "Resilience to Time-Varying Actuator Delays"), and stability under vehicle mass perturbations (Section "Stability under Vehicle Mass Perturbations"). Collectively, these experiments demonstrate that the proposed method possesses not only a theoretically sound architecture but also exceptional robustness and generalization capabilities under highly uncertain and nonlinear conditions.

#### Sensitivity analysis of ASG grouping modes

To deeply investigate the impact of the Action Space Grouping (ASG) mechanism on both the training process and the final control performance of the reinforcement learning agent, this section presents a sensitivity analysis regarding the number of grouping modes, *N*.

Building upon the baseline configuration of *N=16* (illustrated in Fig. 9)—where both the front and rear axles can independently execute four distinct pressure modulation forms: Rapid Decrease (RD), Pressure Hold (PH), Slow Increase (SP), and Rapid Increase (RP)—we propose two additional grouping strategies for comparative evaluation.

The first alternative strategy reduces the granularity to *N=9* by eliminating the option for the inlet solenoid valve to operate at a 30% opening degree. In this configuration, the inlet and outlet valves for both axles are restricted to discrete states that exclude simultaneous 100% opening of both valves, thereby preventing the hazardous scenario of a direct short-circuit between the master cylinder and the reservoir. Consequently, the available action space for each axle is condensed to three forms: Rapid Decrease (RD), Pressure Hold (PH), and Rapid Increase (RP), yielding a total of *N=9* global grouping modes.

The second alternative strategy expands the action space from the baseline *N=16* by introducing a 30% opening degree option for the outlet solenoid valves. In this configuration, both the inlet and outlet valves for the front and rear axles are capable of three discrete opening states: $\left\{ {0\% ,30\% ,100\% } \right\}$.To ensure operational safety, the control logic explicitly excludes the hazardous condition where both inlet and outlet valves are simultaneously fully open (100%), which would cause a direct short-circuit between the master cylinder and the reservoir. Under these constraints, each axle can execute five distinct pressure control modes: the original four—Rapid Decrease (RD), Pressure Hold (PH), Slow Increase (SP), and Rapid Increase (RP)—plus a newly introduced Slow Decrease (SD) mode, achieved by modulating the outlet valve at 30% opening. By independently combining these five modes across the front and rear axles, the system generates a total of 25 unique braking pressure control strategies, resulting in a grouping count of *N=25*.

**Fig. 27:**
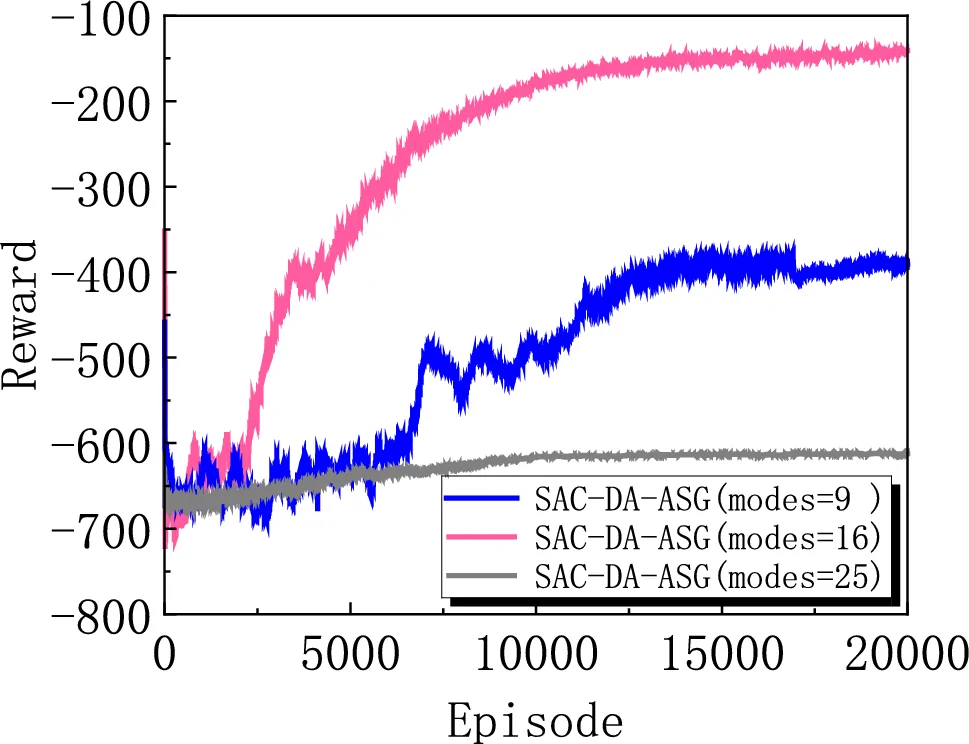
Comparison of agent training reward among action grouping modes (*N=9/16/25*).

Keeping the DA strategy, the neural network architecture (including node counts), and the agent’s hyperparameters constant, we conducted a sensitivity analysis on the impact of different ASG Grouping Modes on the policy training process. The results, illustrated in Fig. 27, reveal distinct performance characteristics across the three configurations:

The baseline configuration (*N=16*) demonstrated superior performance, exhibiting the fastest convergence speed and achieving the highest final reward value. This suggests that the original four-mode granularity per axle provides an optimal balance between control flexibility and learning efficiency.

The *N=9* mode exhibited constrained performance. By eliminating the 30% opening option for the inlet valve, the system lost the capability for Slow Pressure Increase (SP). Consequently, its convergence was significantly slower than that of the *N=16* scheme, and it stabilized at a lower reward level. This indicates that the absence of fine-grained pressure increase regulation causes the agent to frequently experience overshoot or oscillation during pressure tracking tasks, hindering the achievement of smooth pressure control.

Despite introducing the 30% opening option for the outlet valve and adding a Slow Pressure Decrease (SD) mode—which theoretically offers richer control capabilities—the *N=25* mode yielded the poorest training outcomes among the three. Its learning curve not only converged extremely slowly but also maintained low reward values throughout the entire training epoch. We attribute this degradation to two primary factors: 1. Curse of Dimensionality: Expanding the action space to 25 modes significantly enlarged the policy network’s search space, leading to reduced exploration efficiency and slowing down the learning process; 2. Task Irrelevance and Complexity: In the context of the current braking control task, the Slow Pressure Decrease (SD) mode may not be a critical requirement. Its introduction likely increased the complexity of policy selection, causing the agent to easily fall into local optima or make suboptimal decisions. This added complexity effectively offset the potential benefits of finer control granularity.

Therefore, the Sensitivity Analysis of ASG Grouping Modes demonstrates that merely increasing the number of action groups (e.g., *N=25*) does not automatically enhance control performance; on the contrary, an excessively large action space can impede the learning process. Conversely, oversimplifying the action space (e.g., *N=9*) restricts the performance ceiling due to a lack of necessary regulatory granularity.

The *N=16* configuration is proven to be the optimal solution within the current system architecture. It strikes a critical balance by retaining essential fine-grained regulation capabilities (specifically Slow Pressure Increase, SP) while avoiding unnecessary complexity. This configuration provides the most efficient learning environment for the reinforcement learning agent, maximizing both convergence speed and final control efficacy.

#### Performance under split- *μ* road conditions

To evaluate the robustness and adaptability of the proposed method under non-uniform road conditions, two split- *μ* road condition simulation scenarios were designed: Scenario A: The vehicle’s left wheels travel on a low-adhesion surface (*μ =0.5*), while the right wheels travel on a high-adhesion surface (*μ =0.8*); Scenario B: The vehicle’s left wheels travel on a high-adhesion surface (*μ =0.7*), while the right wheels travel on a low-adhesion surface (*μ =0.4*).

**Fig. 28:**
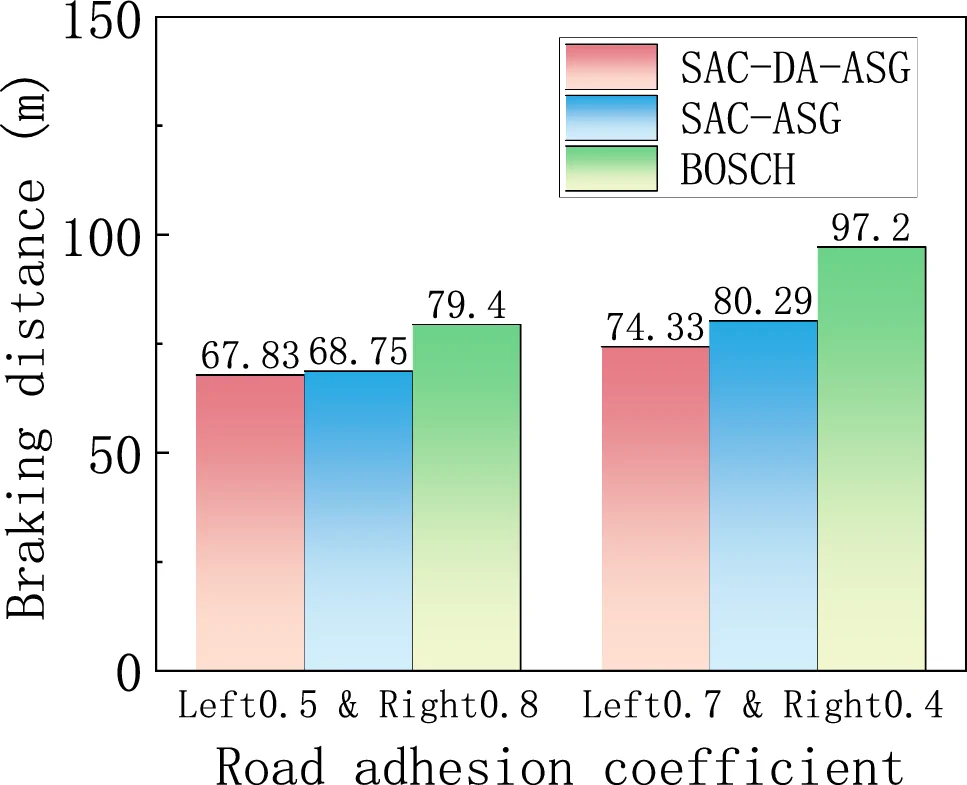
Braking distance comparison under split- *μ* road conditions.

To further quantify the comprehensive braking efficacy of various control strategies under these non-uniform conditions, the final braking distances for both typical split-µ scenarios were statistically analyzed, as illustrated in Fig. 28. The results clearly indicate that the proposed SAC-DA-ASG strategy achieved the shortest braking distance in both test cases, demonstrating superior braking performance compared to other strategies.

**Fig. 29:**
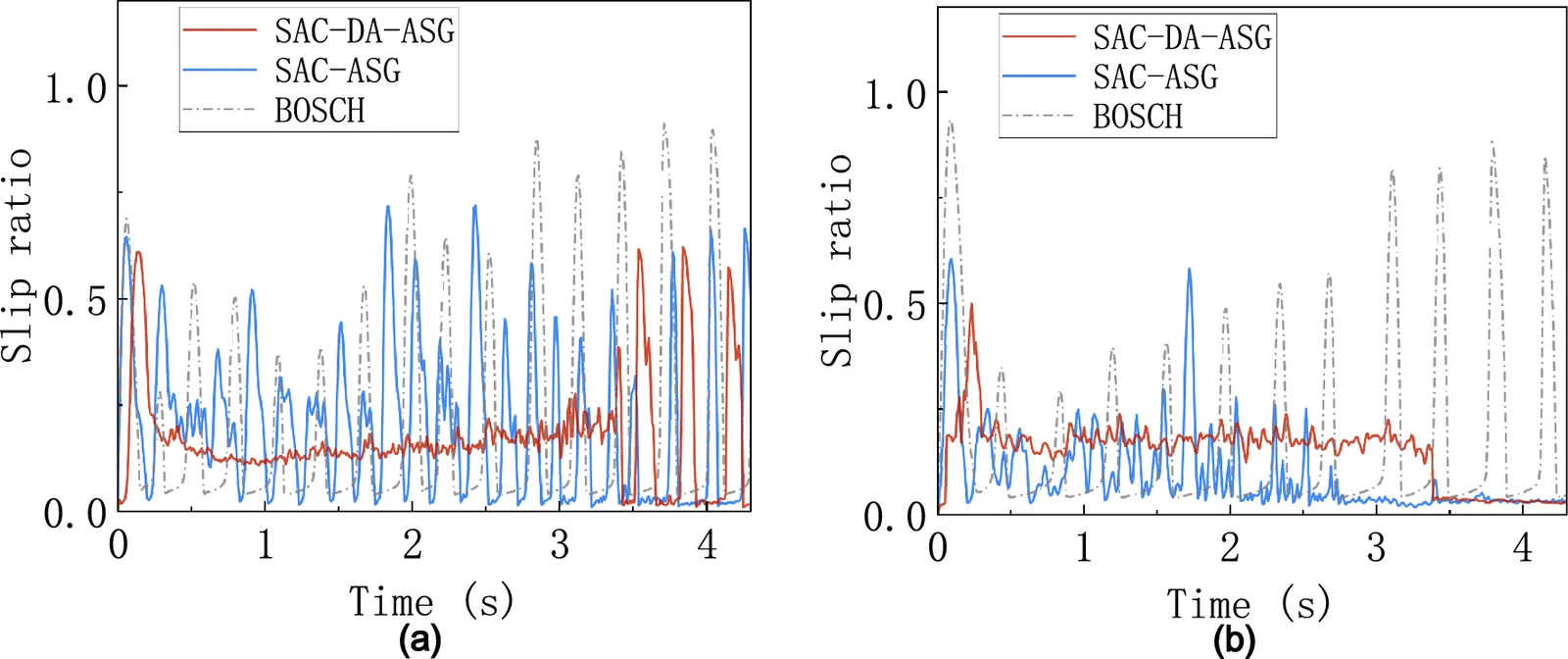
Wheel slip ratio responses under split- *μ* road conditions (left: *μ* =0.5, right: *μ* =0.8). (**a**) FL wheel slip ratio. (**b**)RR wheel slip ratio.

**Fig. 30:**
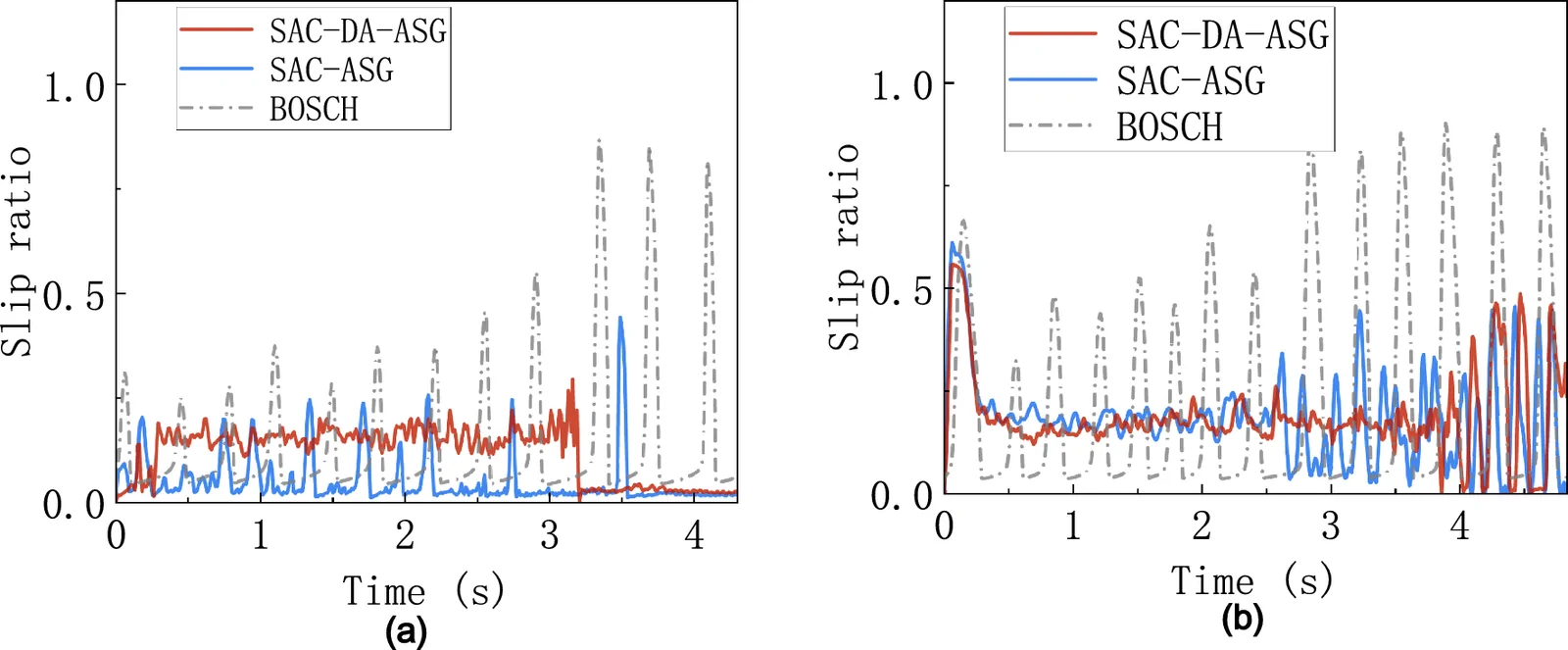
Wheel slip ratio responses under split- *μ* road conditions (left: *μ* =0.7, right: *μ* =0.4). (**a**) (FL wheel slip ratio. (**b**)RR wheel slip ratio.

Figure 29 and Fig. 30 illustrate the slip ratio response curves for the Front-Left (FL) and Rear-Right (RR) wheels under the three control strategies.

The results demonstrate that, across both split-µ scenarios, the proposed SAC-DA-ASG strategy exhibits superior regulation capabilities compared to both the SAC-ASG and the Bosch baseline. Specifically, the SAC-DA-ASG strategy maintains the slip ratio near the optimal value of 0.15 for a significantly longer duration while markedly reducing oscillations.

This performance indicates that the proposed strategy effectively decouples the cross-coupling interference between the left and right wheels. It successfully maximizes the braking potential on the high-adhesion side while precisely suppressing the tendency for wheel lockup on the low-adhesion side. These results validate that the SAC-DA-ASG strategy possesses both strong environmental adaptability and significant engineering practical value when confronting complex, non-uniform road conditions.

#### Resilience to time-varying actuator delays

In the dynamics of braking actuators, solenoid valve response delay is a critical factor determining ABS control performance. In practical vehicle engineering applications, the dynamic characteristics of braking hydraulic solenoid valves are not constant ideal models; influenced by multiple factors such as ambient temperature, the actual response time exhibits time-varying behavior. This is an unavoidable objective reality in real-world vehicle control.

By covering the typical time-varying delay range of 10 ms to 20 ms for braking solenoid valves, we can more accurately map vehicle performance under complex operating conditions, thereby providing a solid basis for the safety and reliability of algorithm deployment^31^. Therefore, building upon the preliminary validation conducted with a nominal response time of 10 ms, this section further performs experiments with braking solenoid valve delays of 15 ms and 20 ms to comprehensively evaluate the algorithm’s sensitivity to time-varying actuator delays.

Table 9 presents a comparative analysis of braking distances for the SAC-DA-ASG strategy across different road friction coefficients ($\mu =\mathrm{0.2,0.5,0.7}$) and varying solenoid valve delays (10 ms, 15 ms, 20 ms). The results indicate that the maximum variation in braking distance observed under different delay conditions is no more than 1.7%. This demonstrates that the proposed strategy exhibits extremely low sensitivity to actuator delay parameters, maintaining superior braking efficacy even under parameter perturbations.

**Table 9:** Comparison of braking distances for the SAC-DA-ASG strategy under different solenoid valve delay experiments.

Road friction coefficient	Solenoid valve delay/ms	Braking distance/m	Variation
*0.2*	10	216.18	-
	15	217.80	↑0.7%
	20	218.00	↑0.8%
*0.5*	10	84.42	-
	15	85.22	↑0.9%
	20	85.89	↑1.7%
*0.7*	10	57.41	-
	15	57.57	↑0.3%
	20	58.06	↑1.1%

**Fig. 31:**
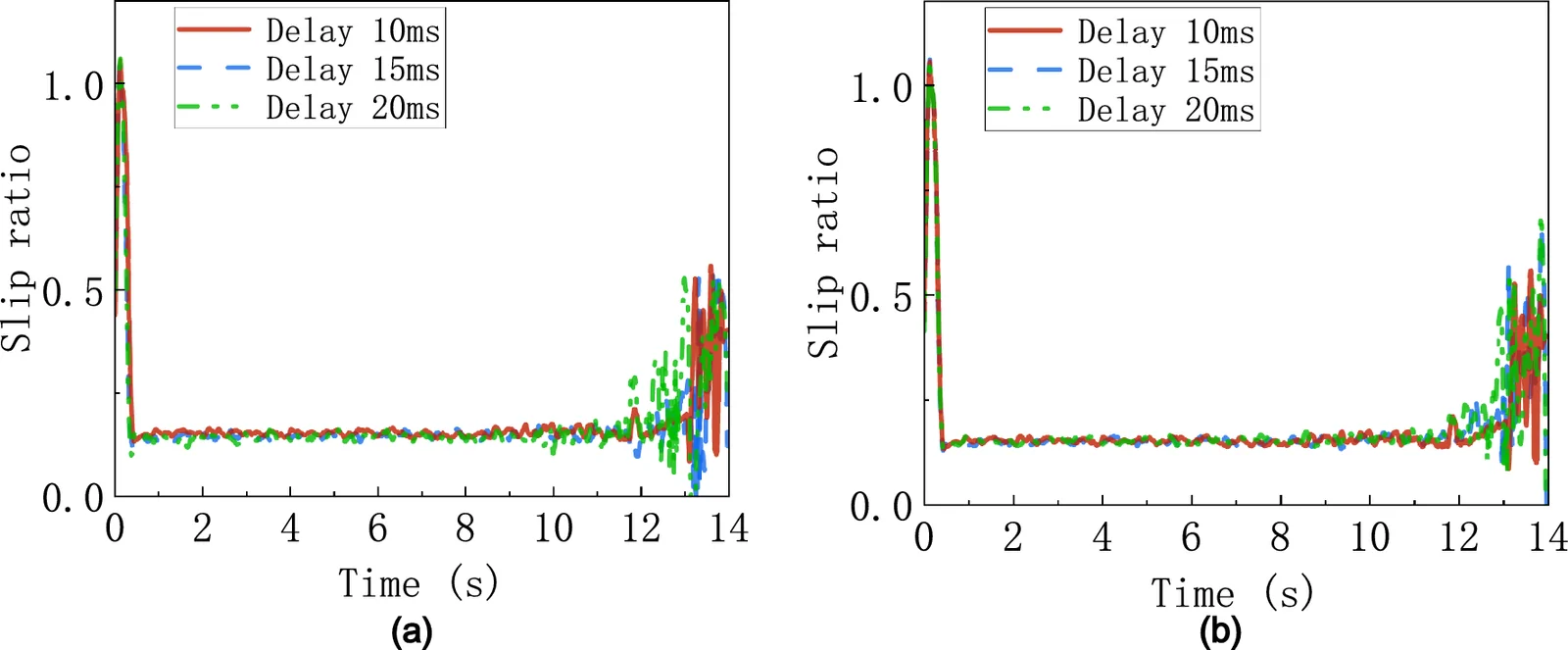
Slip ratio performance of the SAC-DA-ASG strategy under different solenoid valve delay experiments(*μ =0.2*). (**a**) FL wheel slip ratio. (**b**)RL wheel slip ratio.

**Fig. 32:**
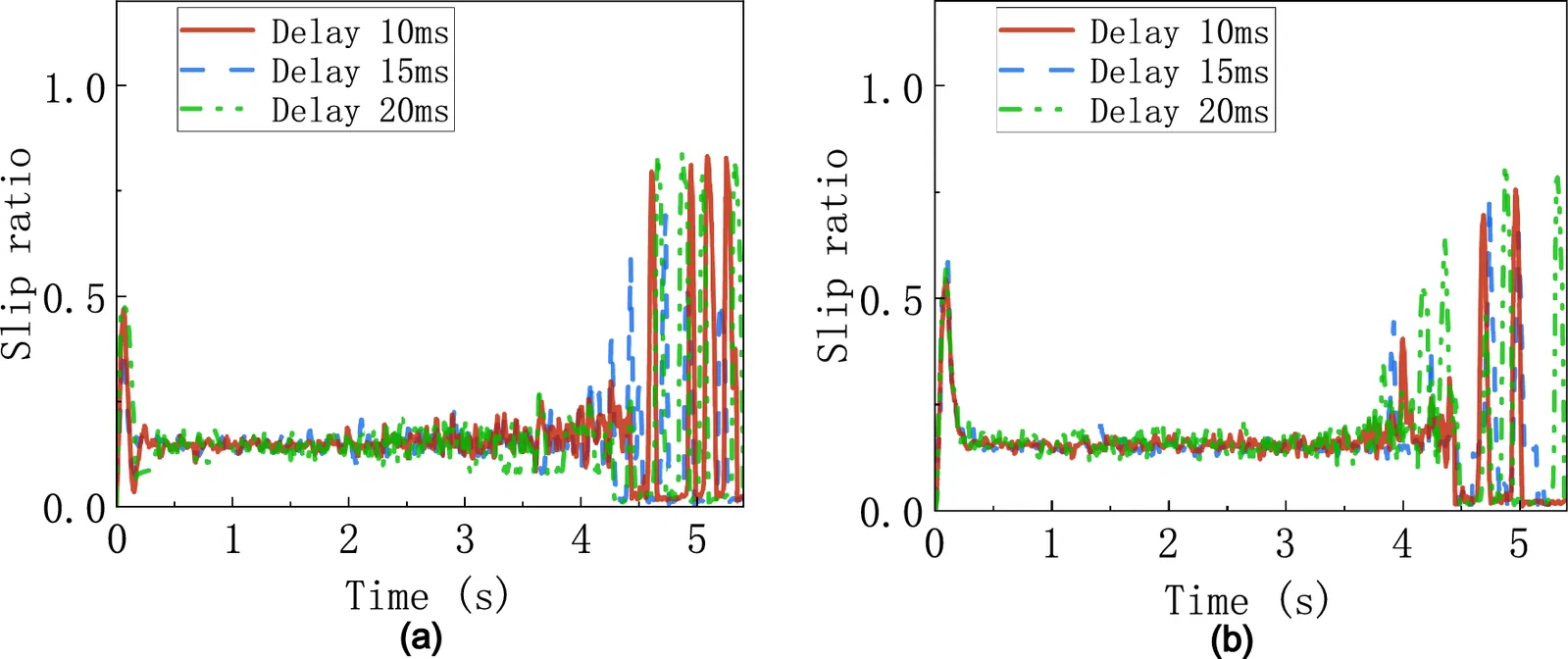
Slip ratio performance of the SAC-DA-ASG strategy under different solenoid valve delay experiments(*μ =0.5*).(**a**) FL wheel slip ratio. (**b**)RL wheel slip ratio.

**Fig. 33:**
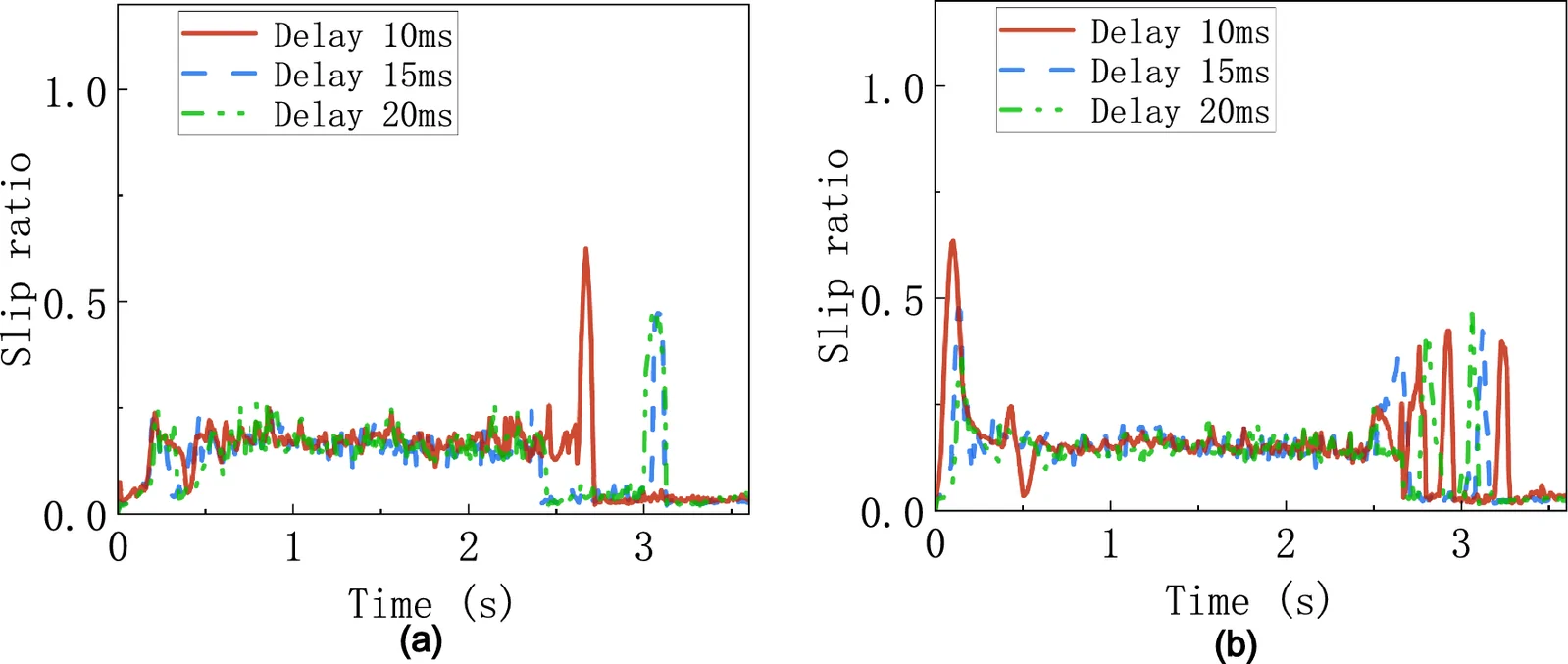
Slip ratio performance of the SAC-DA-ASG strategy under different solenoid valve delay experiments(*μ =0.7*).(**a**) FL wheel slip ratio. (**b**)RL wheel slip ratio.

Furthermore, the slip ratio regulation performance remains highly consistent. As illustrated in Fig. 31,32,33, throughout the entire braking process under various friction coefficients, no systematic deviations in slip ratio attributable to time-varying actuator delays were observed. These findings confirm that the proposed strategy possesses strong robustness against real-world time-varying actuator delays, providing a solid foundation of safety and reliability for its engineering deployment under complex practical operating conditions.

#### Stability under vehicle mass perturbations

In practical engineering applications, vehicle operating conditions are highly complex, with variations in vehicle mass being one of the most common sources of parameter uncertainty. Changes in passenger count or cargo load directly alter the vehicle’s inertial characteristics and tire vertical loads, thereby nonlinearly affecting the maximum adhesion limit and braking dynamic response. To verify the robustness and engineering practicality of the proposed SAC-DA-ASG strategy under parameter perturbations, this paper designed simulation experiments involving vehicle mass disturbances. Based on the nominal mass, test scenarios with 10% mass deviations were established. Tests were conducted under three different road friction coefficients (0.2, 0.5, and 0.7) to validate the algorithm’s robustness across various combinations of friction and load.

Table 10 lists the braking distance test results and their variation rates relative to the initial mass under different conditions. Data analysis reveals that under all tested road adhesion conditions, even with a ± 10% fluctuation in vehicle mass, the SAC-DA-ASG strategy maintained good control stability, with the maximum braking distance deviation controlled within ± 1.2%. This indicates that the strategy exhibits low sensitivity to vehicle mass parameters.

**Table 10:** Comparison of braking distances for the SAC-DA-ASG strategy under vehicle mass disturbance experiments.

Road friction coefficient	Mass variation condition	Braking distance/m	Variation
*0.2*	Mass-10%	214.82	↓0.6%
	Initial Mass	216.18	-
	Mass+10%	218.19	↑0.9%
*0.5*	Mass-10%	83.41	↓1.2%
	Initial Mass	84.42	-
	Mass+10%	84.93	↑0.6%
*0.7*	Mass-10%	56.81	↓1.0%
	Initial Mass	57.41	-
	Mass+10%	57.77	↑0.6%

**Fig. 34:**
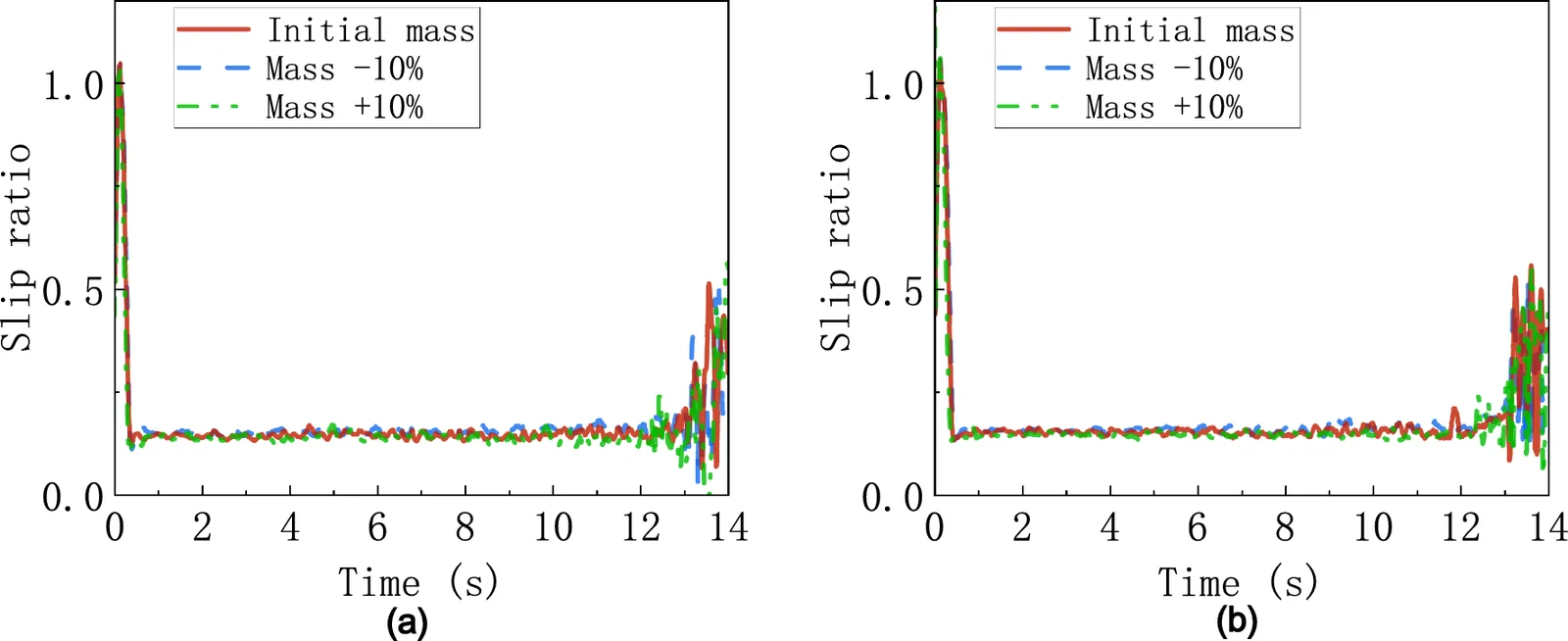
Slip ratio performance of the SAC-DA-ASG strategy under vehicle mass disturbance experiments (*μ =0.2*). (**a**) FL wheel slip ratio. (**b**) RL wheel slip ratio.

**Fig. 35:**
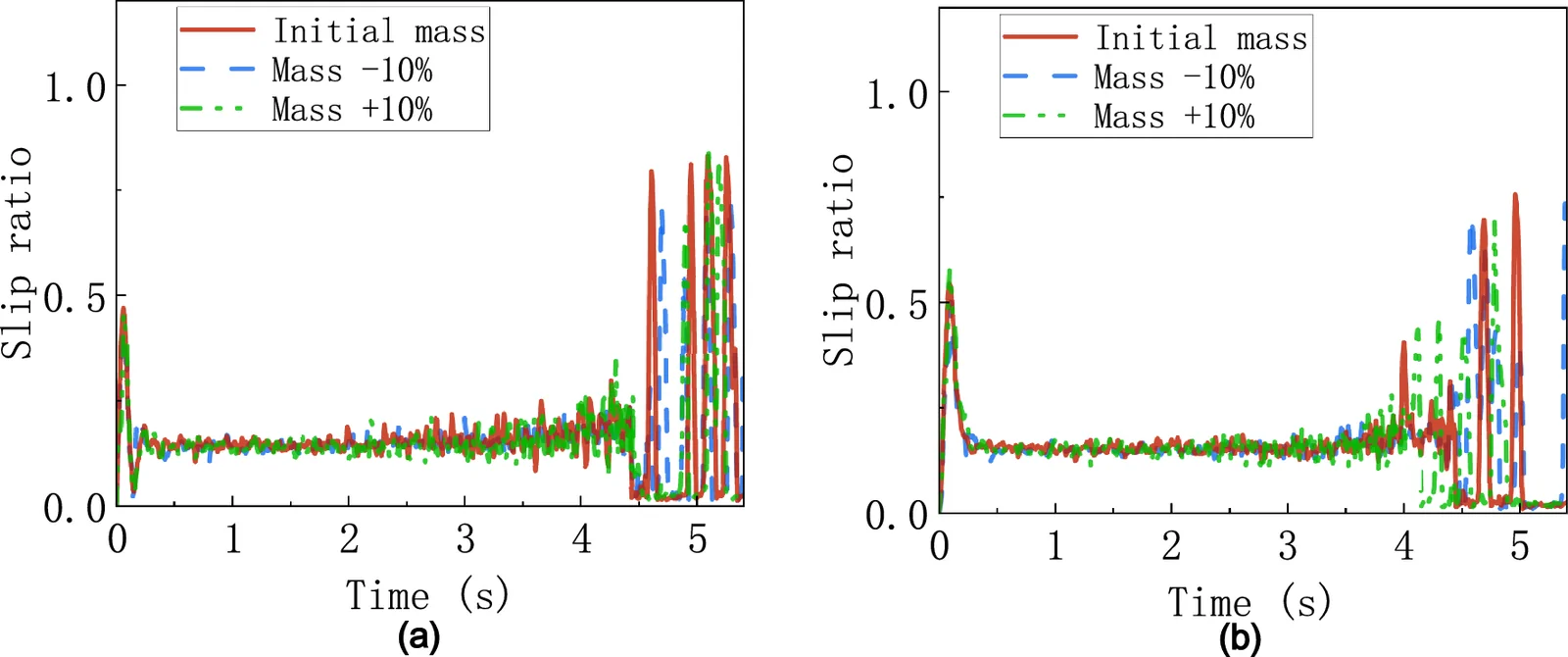
Slip ratio performance of the SAC-DA-ASG strategy under vehicle mass disturbance experiments (*μ =0.5*). (**a**) FL wheel slip ratio. (**b**) RL wheel slip ratio.

**Fig. 36:**
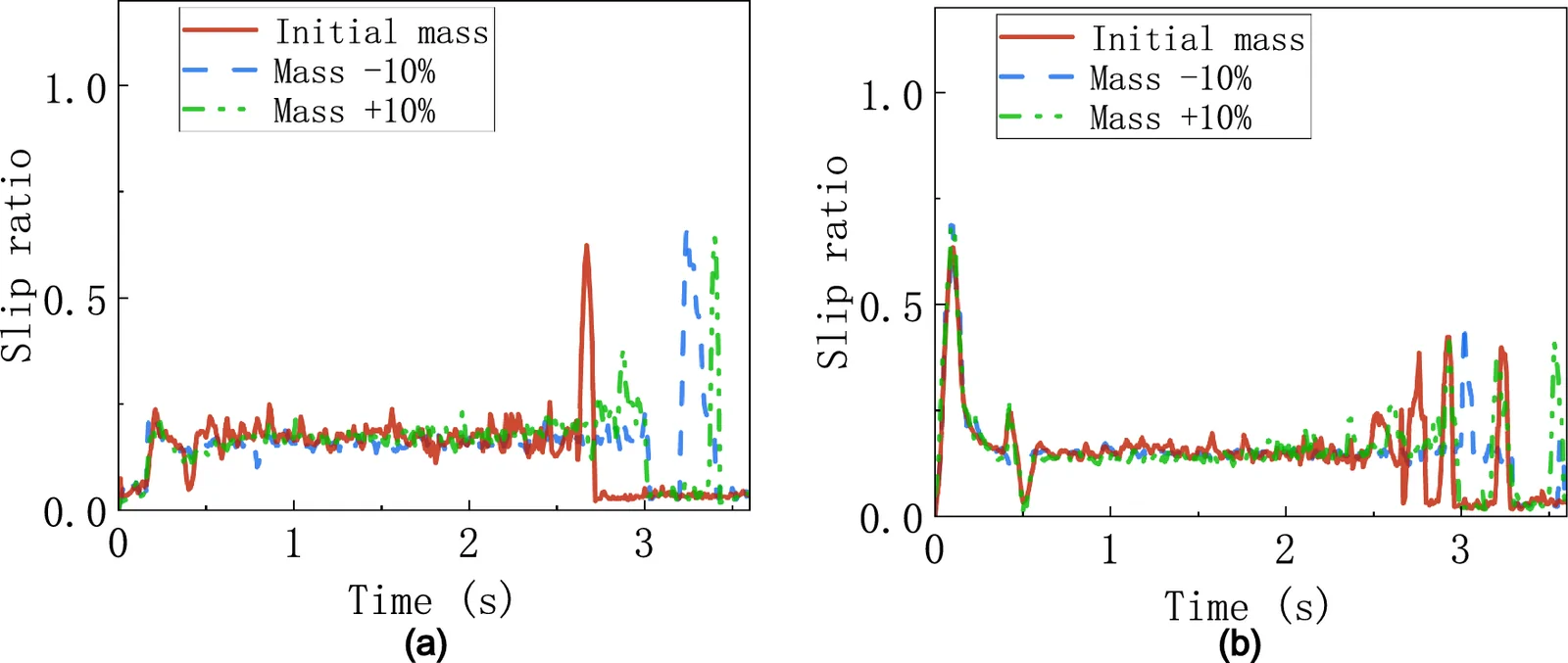
Slip ratio performance of the SAC-DA-ASG strategy under vehicle mass disturbance experiments (*μ =0.7*). (**a**) FL wheel slip ratio. (**b**) RL wheel slip ratio.

Furthermore, the proposed algorithm demonstrated consistent performance in slip ratio regulation under mass variation conditions. As shown in Fig. 34,35,36, throughout the entire braking process under different friction coefficients, no systematic deviations in slip ratio attributable to vehicle mass disturbances were observed. These results prove that the proposed strategy possesses strong robustness against key vehicle mass parameters.

#### Local stability analysis via numerical linearization

In addition to the time-domain simulations presented above, this subsection evaluates the local stability of the proposed controller near the target equilibrium point from a linearized control perspective. As discussed in Sect. 2.4.1, global boundedness is guaranteed by physical constraints. Local stability, however, concerns the ability to attenuate small perturbations around the optimal slip ratio $\lambda^{ * } = 0.15$.

The closed-loop system is formulated as a discrete-time nonlinear system:

38 $${\mathbf{x}}_{k + 1} = {\mathbf{F}}\left( {{\mathbf{x}}_{k} ,{\uppi}_{\theta } \left( {{\mathbf{x}}_{k} } \right),{\mathbf{d}}_{k} } \right)$$

where ${\mathbf{x}}_{k}$ is the state vector (including vehicle speed, wheel angular velocities, wheel cylinder pressures, etc.), ${\uppi}_{\theta }$ is the trained policy, and ${\mathbf{d}}_{k}$ represents external time-varying factors (road adhesion, actuator delay, mass variation). Around the equilibrium point ${\mathbf{x}}^{ * }$ (corresponding to $\lambda^{ * } = 0.15$ and the associated steady-state vehicle speed and braking torques), a first-order Taylor expansion of Eq. (38) yields the linearized system matrix:

39 $${\mathbf{A}}_{cl} = \frac{{\partial {\mathbf{F}}\left( {{\mathbf{x}},{\uppi}_{\theta } \left( {\mathbf{x}} \right),{\mathbf{d}}} \right)}}{{\partial {\mathbf{x}}}}\left| {_{{{\text{x = x}}^{ * } }} } \right.$$

The Jacobian matrix is numerically approximated using the finite difference method, and its spectral radius (the maximum modulus eigenvalue) is computed:

40 $$\rho_{\max } {\mathbf{ = }}\mathop {\max }\limits_{i} \left| {\lambda_{i} \left( {{\mathbf{A}}_{cl} } \right)} \right|$$

If $\rho_{\max } < 1$, the closed-loop system is locally asymptotically stable; otherwise, instability may occur.

To thoroughly assess local stability under various operating conditions, we select representative combinations that cover the dominant influencing factors for numerical linearization. The results are summarized in Table 11.

**Table 11:** Local closed-loop stability indicators under representative conditions.

Road riction	Delay (ms)	Mass condition	​$\rho _{{\max }}$	RMS slip error	Peak slip	Time within [0.10,0.20] (%)
0.2	10	Nominal	0.872	0.030	0.34	89.3
0.2	20	Nominal	0.914	0.034	0.37	87.8
0.2	10	−10%	0.881	0.032	0.35	88.6
0.2	10	+10%	0.886	0.033	0.36	88.1
0.5	10	Nominal	0.836	0.041	0.27	72.7
0.5	20	Nominal	0.882	0.046	0.29	70.4
0.5	10	−10%	0.844	0.042	0.28	72.1
0.5	10	+10%	0.852	0.043	0.28	71.6
0.7	10	Nominal	0.812	0.056	0.24	48.5
0.7	20	Nominal	0.861	0.061	0.26	46.7
0.7	10	−10%	0.821	0.057	0.25	48.0
0.7	10	+10%	0.829	0.058	0.25	47.3

For all tested conditions, the maximum spectral radius $\rho_{\max }$ is less than 1 (ranging from 0.812 to 0.914), indicating that the proposed SAC-DA-ASG controller is locally asymptotically stable around the optimal slip ratio. Even when the actuator delay increases from 10 to 20 ms, the spectral radius remains well below 1 with only a modest increase (maximum increment approximately 0.05), demonstrating the controller’s strong robustness to time-varying delays. Moreover, $\pm 10\mathrm{\%}$ mass variations have only a negligible influence on the spectral radius (variation within 0.02), further confirming the controller’s adaptability to vehicle load changes. In summary, the numerical linearization results are consistent with the time‑domain simulations presented in Section "Policy Sensitivity Analysis and Robustness Verification", jointly confirming the closed‑loop stability of the proposed control strategy under diverse operating conditions.

#### Discussion-robustness enhancement mechanisms for real-world uncertainties

The sensitivity analysis and multi-scenario validation results presented in Sections "Sensitivity Analysis of ASG Grouping Modes" to "Stability under Vehicle Mass Perturbations" demonstrate that the proposed SAC-DA-ASG strategy exhibits superior stability and consistency when subjected to multiple sources of uncertainty, including split- *μ = 0.2* conditions, vehicle mass load variations, and time-varying actuator delays.

This robustness is not coincidental; rather, it stems from the DA mechanism introduced during the algorithm design phase and the inherent generalization capabilities of DRL. Focusing on time-varying actuator delays—a critical challenge in practical vehicle applications—this section further elucidates the core mechanisms and engineering implementation pathways that enhance the strategy’s robustness.

First, parameter randomization is introduced during the reinforcement learning training phase, a key mechanism adopted in this study. Specifically, parameters such as actuator delay and road friction coefficient are not set as fixed values but are treated as random variables spanning their entire operational ranges. This approach enables the agent to explore and optimize more effectively, learning to reserve a safety margin within the action space. Consequently, even when encountering unseen delay fluctuations, the system maintains stability by leveraging the "worst-case" coping strategies acquired during training.

Second, beyond the DA method employed in this research, architectures with temporal memory capabilities, such as Long Short-Term Memory (LSTM) networks or Attention Mechanisms, can be integrated. These structures empower the policy network with the ability to implicitly perceive system dynamic lags, thereby effectively suppressing overshoot and oscillations triggered by sudden changes in delay.

Third, to further solidify safety for real-world deployment, future engineering implementations could adopt a hybrid architecture combining data-driven decision-making and model-driven compensation. In this framework, a feedforward compensation module is introduced to offset the measurable, deterministic portion of the delay before the agent generates its control strategy. This architecture retains the advantages of reinforcement learning in handling complex coupled relationships while utilizing classical control theory to guarantee sufficient phase margin under extreme time-varying delay conditions.

### Real-time HIL performance evaluation and statistical significance validation

#### Real-time HIL validation

**Fig. 37:**
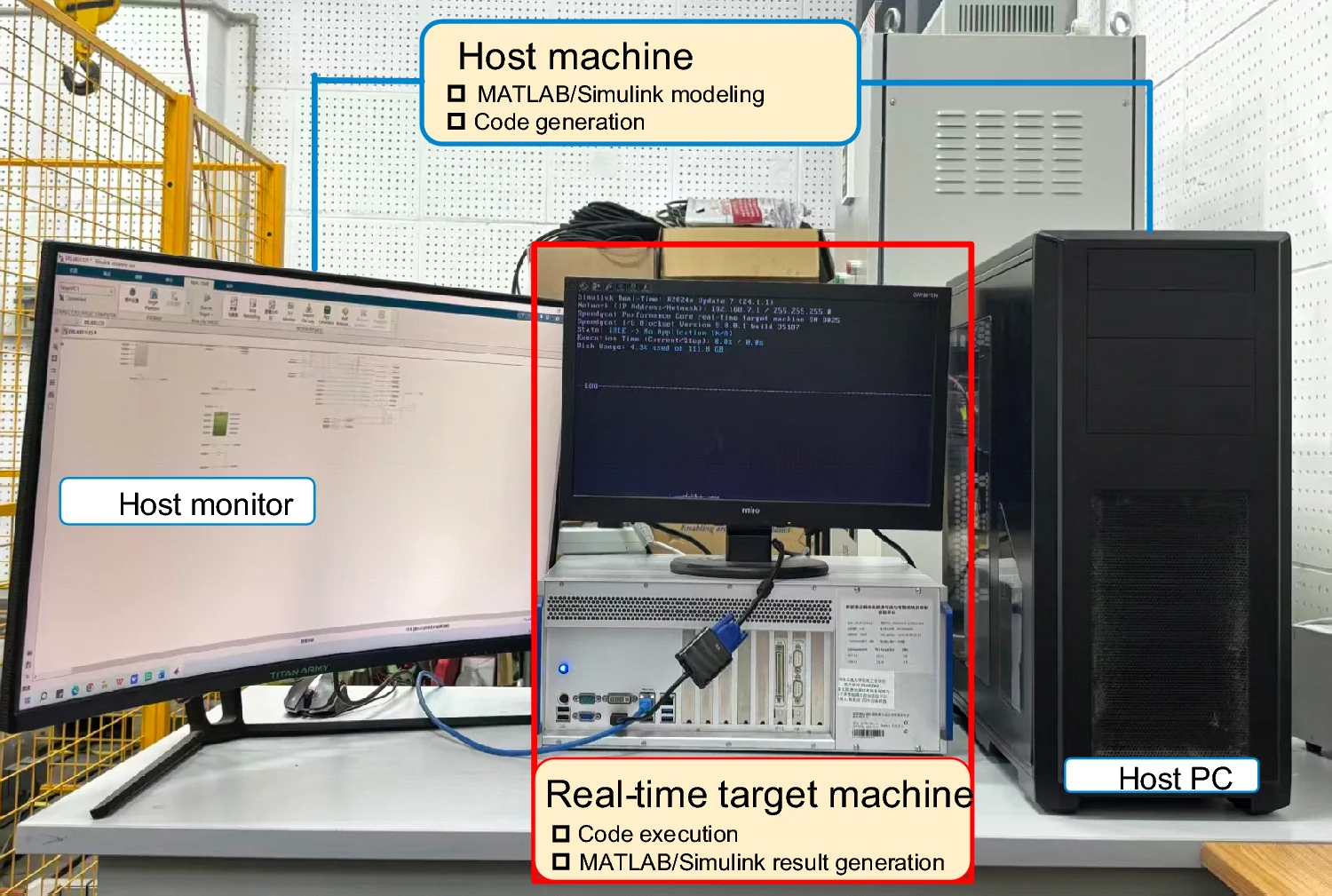
Real-time HIL setup for the validation of the proposed study.

To assess the real-time feasibility and deployment potential of the proposed SAC-DA-ASG controller, we conducted real-time hardware‑in‑the‑loop (HIL) validation. As shown in Fig. 37, the real-time platform comprises a host computer and a real-time target machine. The host plays a central role in both development and control, typically using MATLAB/Simulink to design control algorithms, build simulation models, and configure the real-time target. The host communicates with the target via Transmission Control Protocol/Internet Protocol (TCP/IP). The real-time target was a Speedgoat Performance unit equipped with a 2 GHz quad‑core CPU and an optional Simulink‑programmable FPGA, providing robust real-time performance. The descriptions of the real-time performance target machine are listed in Table 12.

**Table 12:** Details on the specifications of the real-time performance target machine.

Specifications	Speedgoat, Switzerland
Model	DH-PR-TTM-4.2
Processor	Intel Core i7 4.2 GHz, 4 cores
Memory	4 GB
Main drive	1 TB

**Fig. 38:**
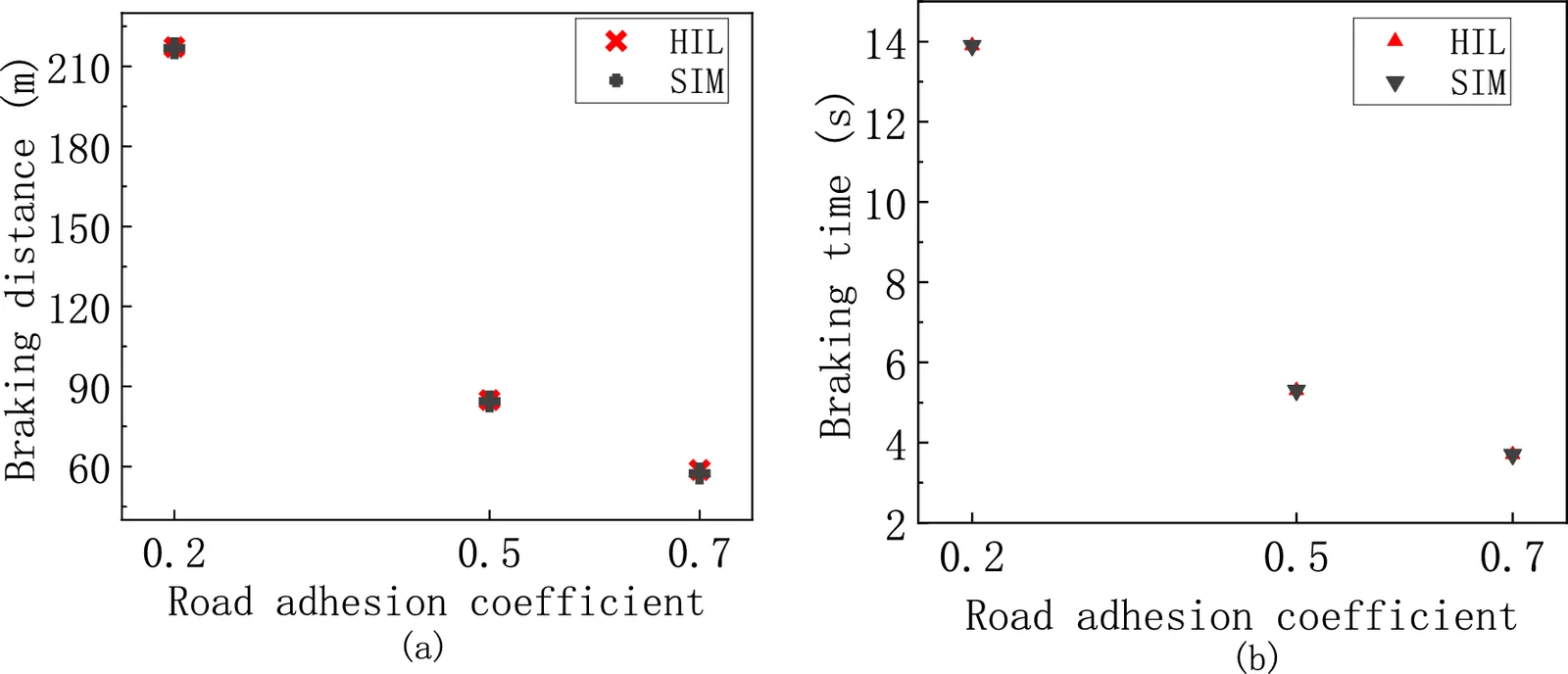
Comparison between HIL and SIM at three road adhesion conditions. (**a**) Braking distances. (**b**) Braking times.

**Fig. 39:**
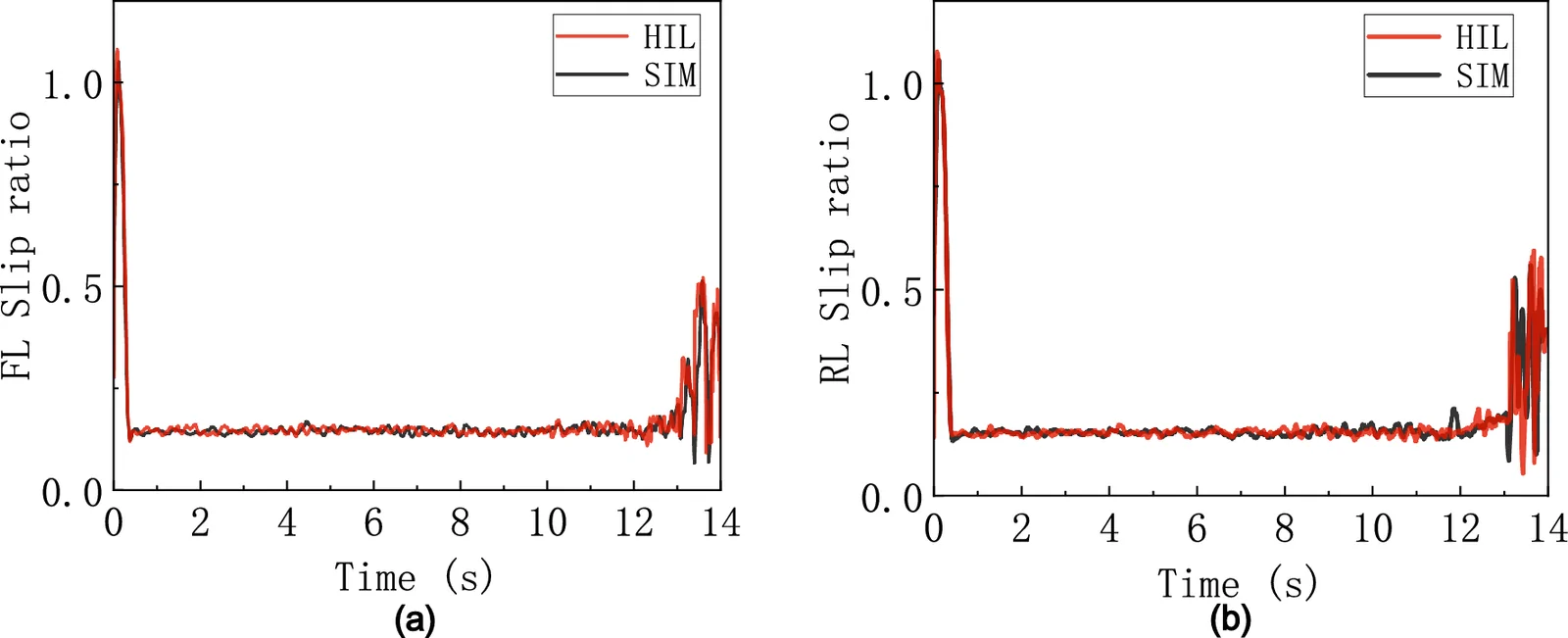
Comparison of wheel slip ratio between HIL and SIM at a road adhesion condition (0.2). (**a**) FL wheel slip ratio. (**b**) RL wheel slip ratio.

**Fig. 40:**
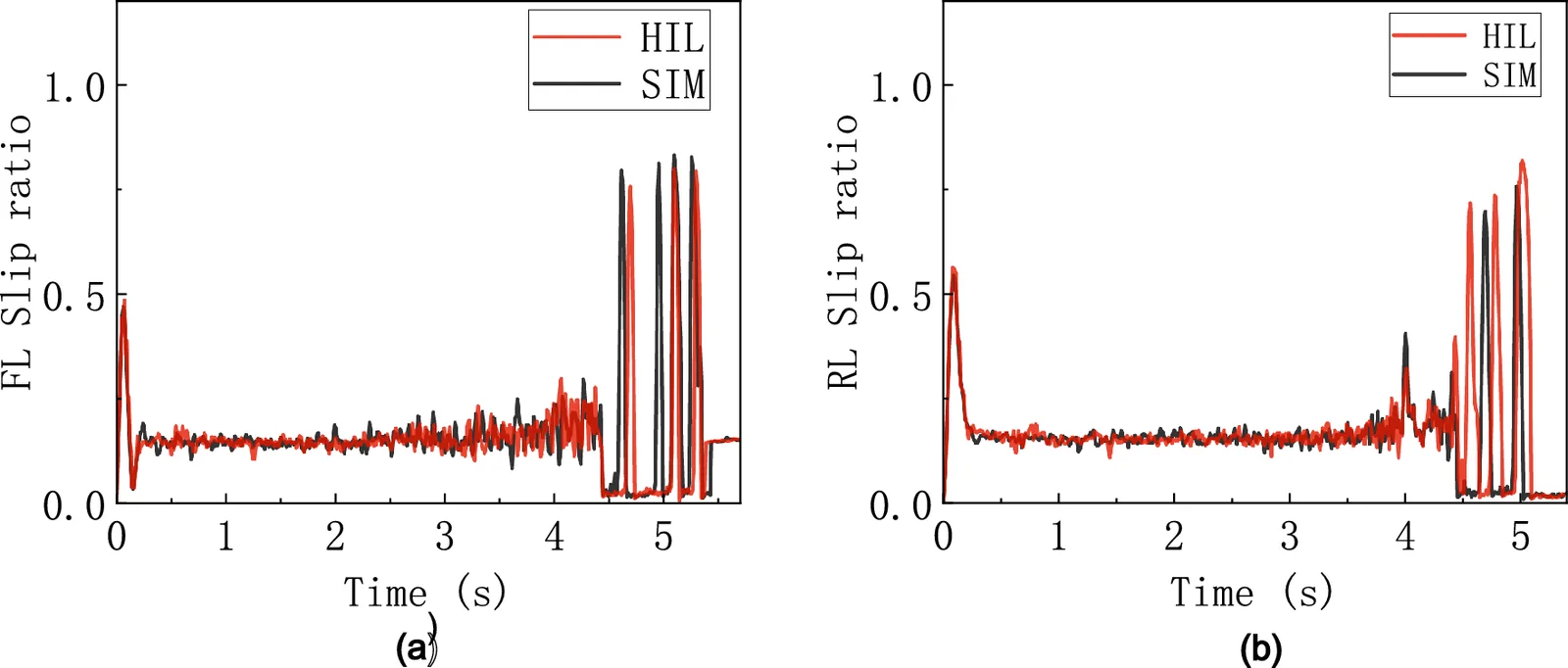
Comparison of wheel slip ratio between HIL and SIM at a road adhesion condition (0.5). (**a**) FL wheel slip ratio. (b) RL wheel slip ratio.

**Fig. 41:**
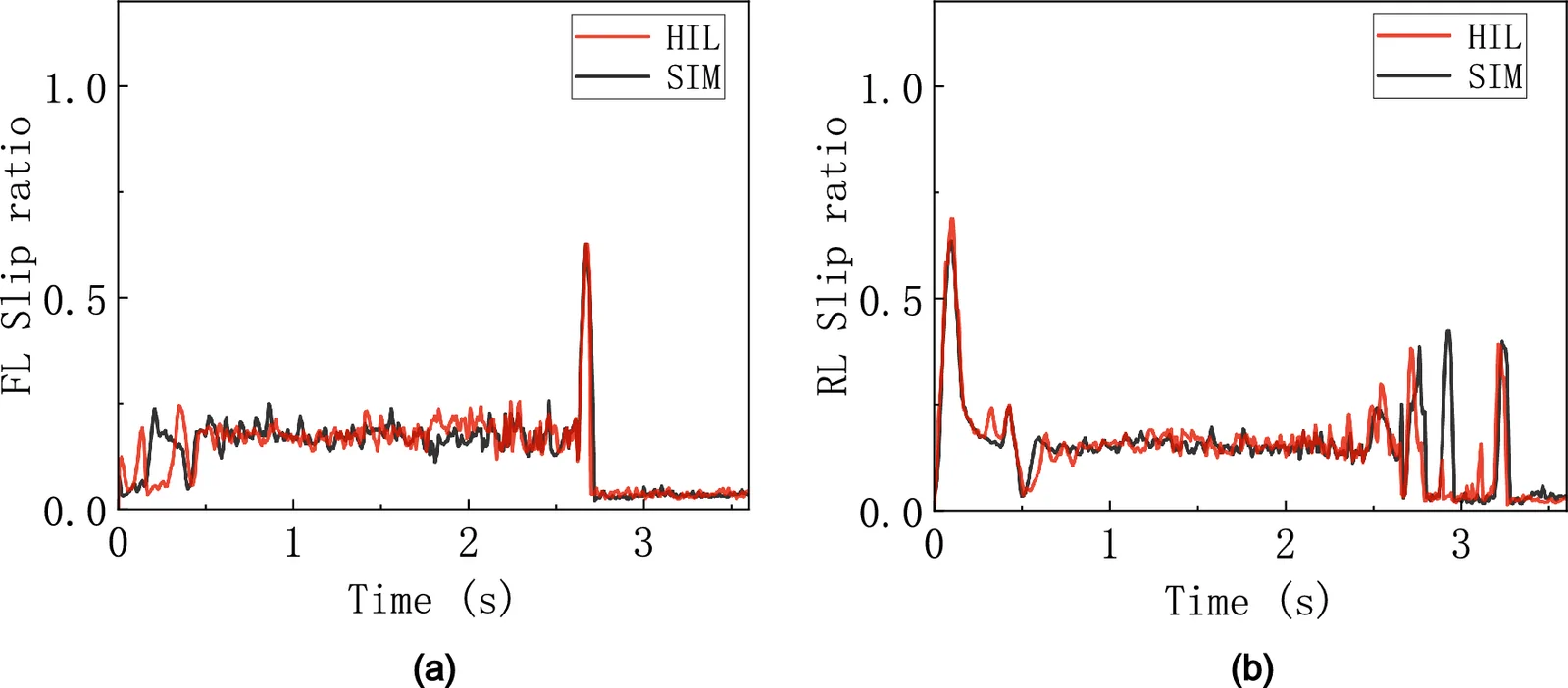
Comparison of wheel slip ratio between HIL and SIM at a road adhesion condition (0.7).

We benchmarked the proposed SAC-DA-ASG controller under three road adhesion conditions—low (0.2), medium (0.5), and high (0.7)—comparing simulation (SIM) with real-time HIL (HIL) results Figs. 37,38,39,40,41. Across all conditions, HIL and SIM deliver closely matched braking time and stopping distance, and exhibit comparable slip‑ratio regulation: No systematic discrepancies are observed between platforms over the full brake range. The experiments confirm that the controller maintains stable control performance and real-time responsiveness, supporting its suitability for in‑vehicle deployment.

#### Real-time performance evaluation

To evaluate the feasibility of deploying the proposed SAC-DA-ASG strategy on mass-production vehicle ECUs, we analyzed the performance of the deployed controller on the Speedgoat real-time platform to assess its resource utilization.

As shown in Table 13, the single complete calculation cycle of the entire control system is approximately 4.12 ms. This result is significantly superior to the 10 ms (100 Hz) requirement common to most real-time control systems, indicating that the strategy possesses extremely low computational latency. The system’s average CPU utilization is approximately 30%, with a memory footprint of 2 GB (including the agent model and runtime cache).

**Table 13:** Hardware resource usage.

**Indicator**	**Value**
Memory Usage	2GB (Includes agent and cache)
CPU Utilization	30%
Total Control Cycle	4.12ms

It is crucial to clarify that this 2 GB memory usage was measured under testing conditions as a total system value. It encompasses both the high-fidelity vehicle dynamics simulation model and the agent control strategy (including network weights and runtime cache). In actual mass-production deployment, the vehicle model will be replaced by the physical vehicle itself; therefore, the memory required for actual deployment corresponds only to the control strategy portion.

However, we must honestly acknowledge that the current memory footprint of the control strategy indeed exceeds the megabyte (MB)-level memory capacity of traditional vehicle ECUs. Nevertheless, this does not imply that the algorithm is inapplicable to mass-production vehicles. Its deployment feasibility is based on considerations from two key dimensions: Although the current memory usage exceeds the limits of traditional ECUs, it falls well within the tolerance range of high-performance vehicle domain controllers, which typically feature gigabyte (GB)-level memory.Through subsequent strategy lightweighting techniques (e.g., quantization, pruning), the memory footprint is expected to be further reduced. In conclusion, our SAC-DA-ASG method is suitable for deployment in vehicles equipped with high-computing-power chips and advanced domain controller architectures.

For the implementation plan targeting mass production deployment, the following steps are required:

1.Code Generation: First, the trained agent must be converted into standardized C + + code using automated code generation tools to ensure compliance with automotive software standards.

2.Integration & Deployment: Second, the agent’s inference tasks should be mapped onto the vehicle domain controller to achieve real-time responsiveness. This involves integrating the controller with vehicle sensors through the AUTOSAR (Automotive Open System Architecture) framework.

3.Safety Mechanisms: Finally, a runtime monitoring mechanism and a rule-based fallback controller must be introduced. If the system detects inference timeouts or abnormal outputs from the intelligent agent, it will immediately switch to traditional safety strategies. This ensures the reliable and safe deployment of the intelligent control strategy in real-world applications.

#### Statistical significance validation

**Fig. 42:**
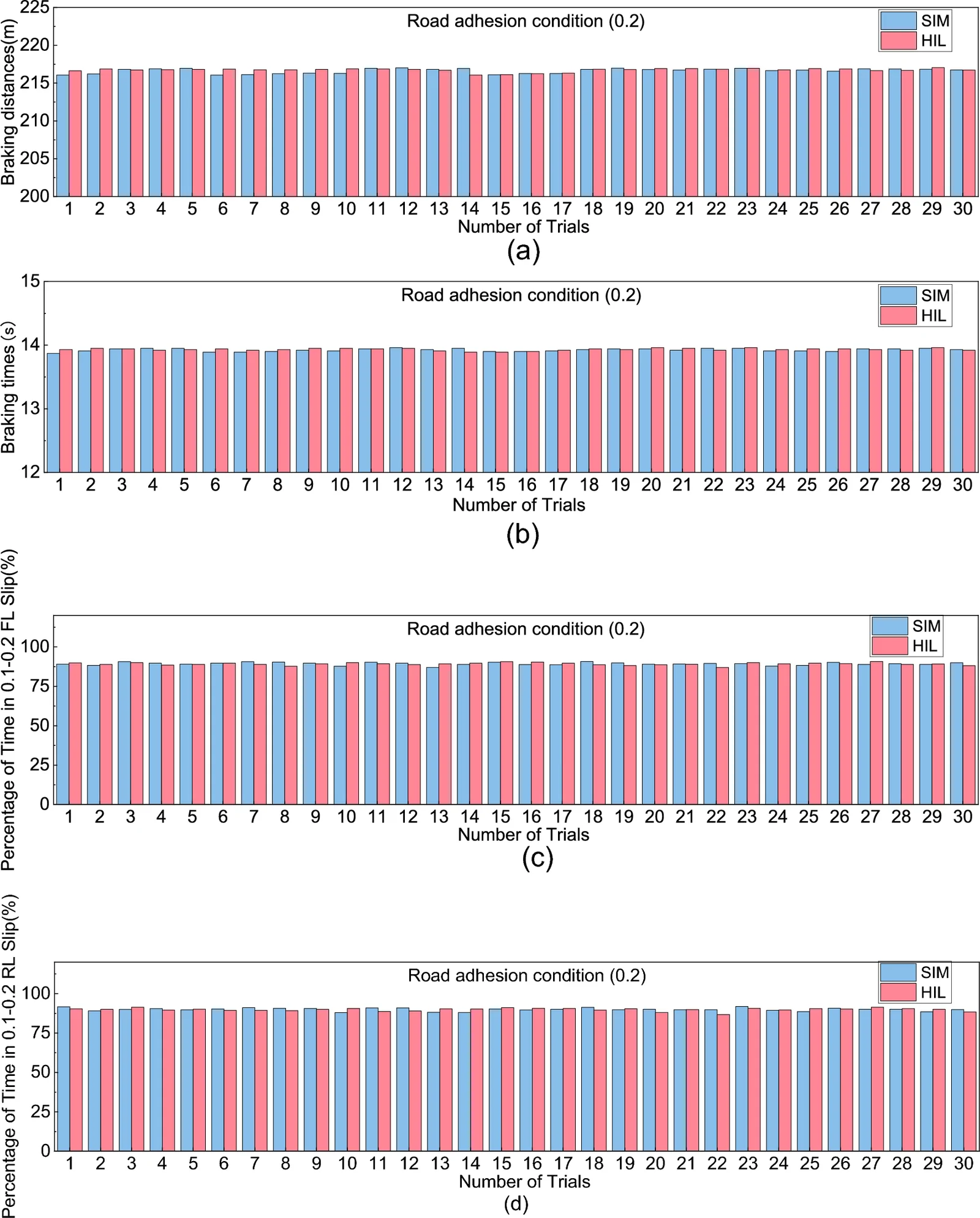
Comparison of 30 independent test results for both SIM and HIL under a road friction coefficient of *μ = 0.2*.

**Fig. 43:**
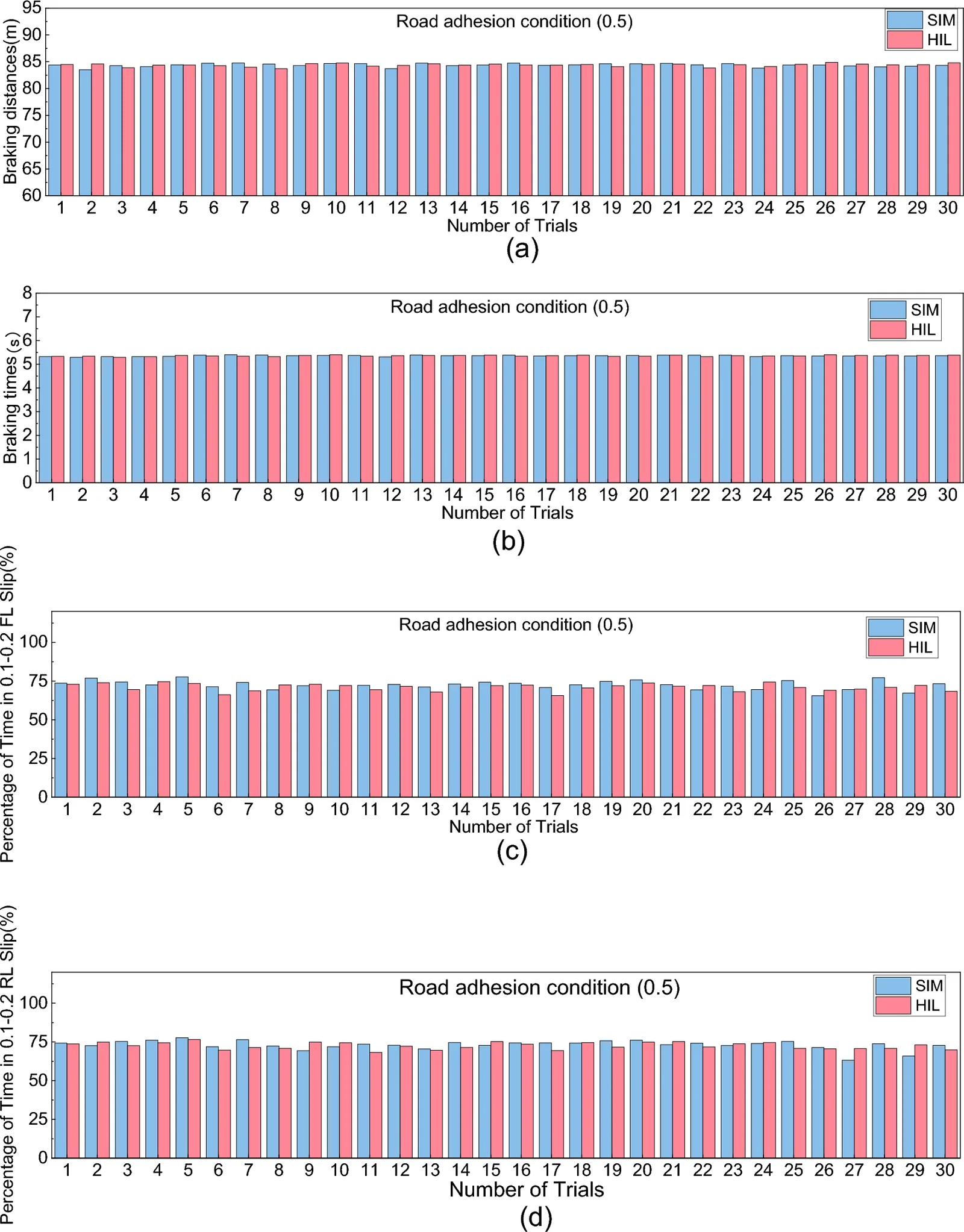
Comparison of 30 independent test results for both SIM and HIL under a road friction coefficient of *μ = 0.5*. (**a**) Braking Distance. (**b**) Braking Time. (**c**) Proportion of time the FL slip ratio remains within the optimal range. (**d**) Proportion of time the RL slip ratio remains within the optimal range.

**Fig. 44:**
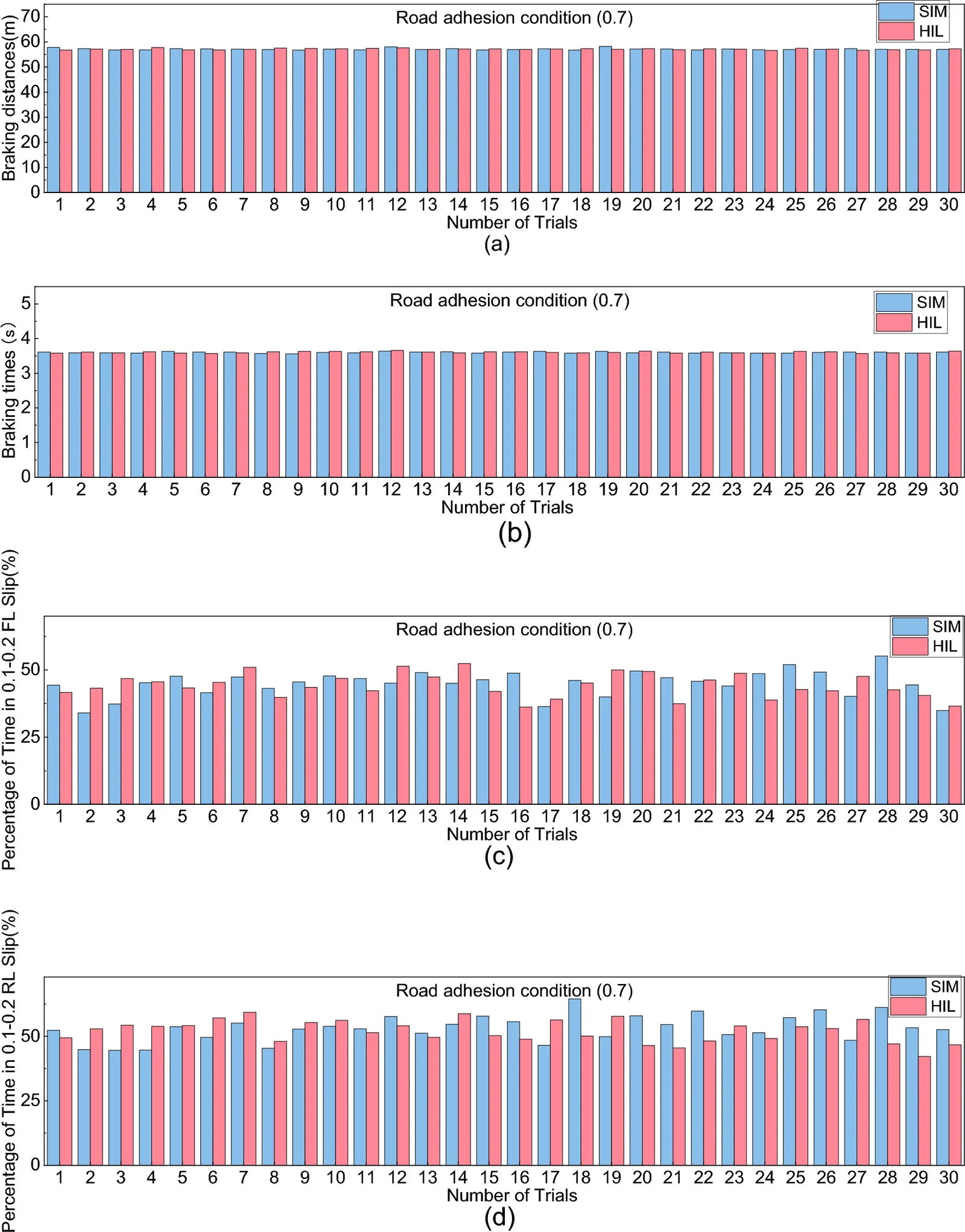
Comparison of 30 independent test results for both SIM and HIL under a road friction coefficient of *μ = 0.7*. (**a**) Braking Distance. (**b**) Braking Time. (**c**) Proportion of time the FL slip ratio remains within the optimal range. (**d**) Proportion of time the RL slip ratio remains within the optimal range.

To rigorously evaluate the stability and repeatability of the proposed SAC-DA-ASG strategy under HIL conditions, and to eliminate the contingency effects of single random trials, we conducted 30 independent SIM tests and 30 real-time HIL tests for each of three typical road friction conditions (0.2, 0.5, and 0.7). This resulted in a cumulative total of 180 valid braking experiments. Given that plotting all slip ratio response curves would lead to visual redundancy and hinder quantitative comparison, this study employs a statistical feature analysis method. Three key performance indicators were extracted for quantitative evaluation: 1. Braking Distance; 2. Braking Time; 3. Slip Ratio Stability Ratio: The proportion of the total braking duration during which the slip ratio remains stable within the range of *0.15± 0.05*. The corresponding results are illustrated in Figs. 42,43,44 Furthermore, the mean and. Furthermore, the mean and variance of these key performance indicators were calculated and are presented in Tables 14,15,16.

**Table 14:** Comparison of results from 30 SIM and 30 HIL tests (*μ = 0.2*).

**Comparison Category**	**SIM**	**HIL**
Mean Braking Distance (m)	216.65	216.76
Variance of Braking Distance (m^2^)	0.1186	0.0864
Mean Braking Time (s)	13.92	13.93
Variance of Braking Time (s^2^)	0.0006	0.0005
Mean FL Slip Ratio Optimal Zone Time Ratio (%)	89.25	89.24
Variance of FL Slip Ratio Optimal Zone Time Ratio (%^2^)	0.0096	0.0099
Mean RL Slip Ratio Optimal Zone Time Ratio (%)	89.96	89.86
Variance of RL Slip Ratio Optimal Zone Time Ratio (%^2^)	0.0128	0.0158

**Table 15:** Comparison of results from 30 SIM and 30 HIL tests (*μ = 0.5*).

**Comparison Category**	**SIM**	**HIL**
Mean Braking Distance (m)	84.45	84.43
Variance of Braking Distance (m^2^)	0.0765	0.0826
Mean Braking Time (s)	5.36	5.35
Variance of Braking Time (s^2^)	0.00049	0.00054
Mean FL Slip Ratio Optimal Zone Time Ratio (%)	72.49	71.19
Variance of FL Slip Ratio Optimal Zone Time Ratio (%^2^)	0.0862	0.0676
Mean RL Slip Ratio Optimal Zone Time Ratio (%)	72.96	72.46
Variance of RL Slip Ratio Optimal Zone Time Ratio (%^2^)	0.0984	0.0766

**Table 16:** Comparison of results from 30 SIM and 30 HIL tests (*μ = 0.7*).

**Comparison Category**	**SIM**	**HIL**
Mean Braking Distance (m)	57.06	57.14
Variance of Braking Distance (m^2^)	0.12	0.10
Mean Braking Time (s)	3.60	3.60
Variance of Braking Time (s^2^)	0.0004	0.0006
Mean FL Slip Ratio Optimal Zone Time Ratio (%)	44.68	44.16
Variance of FL Slip Ratio Optimal Zone Time Ratio (%^2^)	0.0293	0.0238
Mean RL Slip Ratio Optimal Zone Time Ratio (%)	53.29	51.76
Variance of RL Slip Ratio Optimal Zone Time Ratio (%^2^)	0.0246	0.0169

The analysis reveals that the mean values of the three key performance indicators obtained from SIM and HIL tests are highly consistent. Moreover, the variances for both test environments are at a low magnitude and show strong convergence. These findings provide robust evidence that the proposed control strategy possesses excellent stability and repeatability across different testing platforms and stochastic conditions.

## Conclusion

This study focuses on in-wheel motor electric vehicles and proposes an improved DRL-based anti-lock braking control strategy. The approach introduces DRL into the electro-hydraulic composite braking domain for in-wheel motor-driven electric vehicles. Building on the widely adopted DRL algorithm SAC, two key enhancements are integrated. First, a dimensionality reduction method via action space grouping (ASG) is introduced, which reconfigures discrete actions from multiple actuators into a one-dimensional grouped action, significantly reducing the dimensionality of the DRL action space. Second, an augmented state space is employed to enable delay awareness(DA), allowing the agent to account for response time discrepancies between actuators. These improvements form the basis of the SAC-DA-ASG framework. Experimental results demonstrate that the proposed method achieves superior training performance and efficiency compared to the baseline SAC. Furthermore, in comparison with the industry-standard Bosch control algorithm, SAC-DA-ASG maintains wheel slip ratios closer to the optimal value across various road adhesion conditions, with reduced fluctuation and lower peak slip ratios. A parallelization architecture for DRL training is also developed, leveraging asynchronous experience replay and distributed environment interaction to accelerate the training process. The main conclusions are summarized as follows:

1. Compared to other control strategies, the proposed SAC-DA-ASG strategy effectively reduces braking distance while maintaining the wheel slip ratio closer to the optimal value of 0.15 for a longer duration and with lower fluctuation. It also significantly reduces peak slip ratios, thereby improving overall braking stability. Furthermore, experiments conducted under split- road conditions , resilience to time-varying actuator delays, and vehicle mass perturbations demonstrate that the proposed SAC-DA-ASG strategy possesses robust performance.
2. Real‑time HIL and SIM deliver closely matched braking time and stopping distance and exhibit comparable slip‑ratio regulation, confirming stable control performance and real‑time responsiveness suitable for deployment. Statistical significance validation further confirms that the proposed strategy exhibits excellent stability and repeatability.
3. The proposed parallelization architecture for DRL training significantly accelerates the training process, achieving approximately a 5.14× improvement in convergence speed.

In summary, the proposed strategy demonstrates strong performance in coordinating MRBS and HBS for ABS control in in-wheel motor-driven electric vehicles with electro-hydraulic composite braking systems. It effectively leverages the advantages of both braking subsystems and significantly enhances the overall anti-lock braking performance of in-wheel motor electric vehicles.

Despite these results, this study has several limitations that also point to avenues for future work. First, although all model parameters and actuator characteristics in this paper are derived from the prototype vehicle platform shown in Fig. 3, brake lock-up constitutes an extreme operating condition. We currently lack access to a closed proving ground and the requisite safety infrastructure to conduct such on-vehicle tests; therefore, validation in this study relies on simulation and real-time hardware-in-the-loop testing.

Second, the present reward design focuses primarily on slip control and stability. Future designs will incorporate explicit multi-objective terms and constraints to address energy recovery, comfort/NVH (noise, vibration, and harshness), thermal limits, and brake wear—potentially via constrained reinforcement learning, Pareto reinforcement learning, or inverse reinforcement learning.

Third, and critically, the current framework is developed exclusively for longitudinal braking dynamics. While effective in straight-line scenarios, its integration into a holistic vehicle chassis architecture requires further investigation. Future work will focus on developing a hierarchical coordination strategy to enable interaction with lateral stability systems (e.g., Electronic Stability Control, ESC) and traction control systems (TCS). Specifically, we aim to define how the longitudinal slip targets generated by our controller can serve as dynamic constraints for ESC yaw moment distribution during combined braking-cornering maneuvers, and how the adaptive reward mechanism can be reconfigured for TCS to prevent wheel spin during acceleration. Addressing these coupling issues is essential for transitioning from a standalone braking controller to a unified chassis domain controller capable of handling complex, multi-dimensional vehicle dynamics.

## Data Availability

The datasets generated and/or analyzed during the current study are available from the corresponding author on reasonable request.
